# Abstracts of the Fifth Brainstorming Research Assembly for Young Neuroscientists (BraYn), Italy, 28–30 September 2022

**DOI:** 10.3390/neurolint15010028

**Published:** 2023-03-14

**Authors:** Giovanni Ferrara

**Affiliations:** Experimental Neuroscience Lab, IRCCS Ospedale Policlinico San Martino, 16132 Genoa, Italy; giovanni.ferrara@hsanmartino.it

**Keywords:** neuroimaging, neuroinflammation, neurophysiology & neural plasticity, neuro-oncology, neurodegeneration, neuro-oncology, epilepsy, brain development & neurogenetics, clinical neuroscience

## Abstract

On behalf of the BraYn Association Ets, we are pleased to present the Abstracts of the Fifth Brainstorming Research Assembly for Young Neuroscientists, which was held in Rome, Italy from 28–30 September 2022. We congratulate all the presenters on their research work and contribution.

## 1. Mutations in the New Disease-Causing Gene ARF3 Have Disruptive Consequences on Golgi Integrity and Brain Development

Giulia Fasano ^1,†^, Valentina Muto ^1,†^, Francesca Clementina Radio ^1,†^, Martina Venditti ^1^, Niloufar Mosaddeghzadeh ^2^, Simona Coppola ^3^, Graziamaria Paradisi ^1,4^, Erika Zara ^1,5^, Farhad Bazgir ^2^, Alban Ziegler ^6,7^, Giovanni Chillemi ^4,8^, Lucia Bertuccini ^9^, Antonella Tinari ^10^, Annalisa Vetro ^11^, Francesca Pantaleoni ^1^, Simone Pizzi ^1^, Libenzio Adrian Conti ^12^, Stefania Petrini ^12^, Alessandro Bruselles ^13^, Ingrid Guarnetti Prandi ^4^, Cecilia Mancini ^1^, Balasubramanian Chandramouli ^14^, Magalie Barth ^7^, Céline Bris ^6,7^, Donatella Milani ^15^, Angelo Selicorni ^16^, Marina Macchiaiolo ^1^, Michaela V Gonfiantini ^1^, Andrea Bartuli ^1^, Riccardo Mariani ^17^, Cynthia J Curry ^18^, Renzo Guerrini ^11^, Anne Slavotinek ^18^, Maria Iascone ^19^, Bruno Dallapiccola ^1^, Mohammad Reza Ahmadian ^2^, Antonella Lauri ^1,^*^,‡^ and Marco Tartaglia ^1,^*^,‡^

^1^ Genetics and Rare Diseases Research Division, Ospedale Pediatrico Bambino Gesù, IRCCS, 00146 Rome, Italy

^2^ Institute of Biochemistry and Molecular Biology II, Medical Faculty and University Hospital Düsseldorf, Heinrich Heine University Düsseldorf, 40225 Düsseldorf, Germany

^3^ National Center for Rare Diseases, Istituto Superiore di Sanità, 00161 Rome, Italy

^4^ Department for Innovation in Biological Agro-food and Forest systems (DIBAF), University of Tuscia, 01100 Viterbo, Italy

^5^ Department of Biology and Biotechnology “Charles Darwin”, Università “Sapienza”, 00185 Rome, Italy

^6^ UFR Santé de l’Université d’Angers, INSERM U1083, CNRS UMR6015, MITOVASC, SFR ICAT, F-49000 Angers, France

^7^ Département de Génétique, CHU d’Angers, 49000 Angers, France

^8^ Institute of Biomembranes, Bioenergetics and Molecular Biotechnologies, Centro Nazionale delle Ricerche, 70126 Bari, Italy

^9^ Servizio Grandi Strumentazioni e Core Facilities, Istituto Superiore di Sanità, 00161 Rome, Italy

^10^ Centro di Riferimento per la Medicina di Genere, Istituto Superiore di Sanità, 00161 Rome, Italy

^11^ Pediatric Neurology, Neurogenetics and Neurobiology Unit and Laboratories, Meyer Children’s Hospital, University of Florence, 50139 Florence, Italy

^12^ Confocal Microscopy Core Facility, Ospedale Pediatrico Bambino Gesù, IRCCS, 00146 Rome, Italy

^13^ Department of Oncology and Molecular Medicine, Istituto Superiore di Sanità, 00161 Rome, Italy

^14^ Super Computing Applications and Innovation, CINECA, 40033 Casalecchio di Reno, Italy

^15^ Fondazione IRCCS Ca’ Granda Ospedale Maggiore Policlinico, 20122 Milan, Italy

^16^ Mariani Center for Fragile Children Pediatric Unit, Azienda Socio Sanitaria Territoriale Lariana, 22100 Como, Italy

^17^ Department of Laboratories Ospedale Pediatrico Bambino Gesù, IRCCS, 00146 Rome, Italy

^18^ Genetic Medicine, Department of Pediatrics, University of California San Francisco, San Francisco, CA 94143, USA

^19^ Medical Genetics, ASST Papa Giovanni XXIII, 24127 Bergamo, Italy

* Correspondence: antonella.lauri@opbg.net (A.L.); marco.tartaglia@opbg.net (M.T.)

† These authors contributed equally to this work.

‡ These authors contributed equally to this work.

Rare diseases affect more than 400 million people worldwide. Most of these conditions are characterized by highly disabling malformations of cortical development (MCD). Yet, despite the recent increase in disease-genes and variant, discovery, heterogenous MCD remain without treatment due to poor knowledge of the underlying mechanisms. Here, we employed an integrated functional genomics pipeline comprising human exome sequencing, in silico, in vitro, and in vivo cell/developmental biology analysis using in vitro systems and zebrafish to tackle a previously unidentified disease showing variable degrees of microcephaly, cortical atrophy, and thinning of the corpus callosum associated with skeletal anomalies. We identified de novo missense variants affecting ARF3, a far neglected member of small GTPases of the RAS superfamily involved in Golgi-trafficking, as causative of the disease, and provide first insights into ARF3 activity throughout vertebrate embryogenesis. In silico and biochemical investigations demonstrated that microcephaly-causing ARF3 mutations affect highly conserved residues regulating the catalytic activity of the protein participating in GTP binding. Experiments in fish embryos corroborated this finding and proved the disruptive consequences of aberrant ARF3 on trans-Golgi integrity. Comparable in vitro results substantiated the pathophysiological role the newly discovered ARF3 mutations, leading to various patterns of Golgi dysfunction, as an underlying mechanism of this new form of Golgipathy. Our zebrafish models further validated the occurrence of a severe microcephalic trait caused by the severe mutations. The data showed a fundamental perturbation of precursor cells proliferation in the developing forebrain as well as planar cell polarity (PCP)-dependent cell processes establishing the body plan axes, resembling a known effect caused by dominant mutations in ARF1. In conclusion, utilizing an integrated multi-level analysis (genomics, in silico, in vitro and in vivo), our work: (1) provides molecular classification for disease stratification, (2) offers a mechanistic knowledge of a previously unrecognized neurodevelopmental disorder, and (3) documents an obligate dependence on proper ARF3 function for Golgi homeostasis and early developmental processes.

## 2. Patients Derived Organoids Show Differences in DNA Damage Accumulations in Neural Progenitors Leading Microcephaly Syndrome

Gianmarco Pallavicini ^1^, Amanda Moccia ^2^, Roberta Parolisi ^1^, Giorgia Iegiani ^1^, Martina Lorenzati ^1^, Fiorella Balzac ^3^, Emilia Turco ^3^, Enrica Boda ^1^, Annalisa Buffo ^1^, Stephanie Bielas ^2^ and Ferdinando Di Cunto ^1^

^1^ Neuroscience Institute Cavalieri Ottolenghi, University of Turin, Department of Neuroscience “Rita Levi Montalcini”, Torino, Italy

^2^ The University of Michigan Medical School, Department of Human Genetics, Ann Arbor, MI, USA 

^3^ Department of Molecular Biotechnology and Health Sciences, Molecular Biotechnology Center, University of Turin, Torino, Italy

In primary hereditary microcephaly (MCPH), brain volume reduction is the main clinical phenotype, associated with conserved brain architecture and mild to moderate intellectual disability. Mutations in citron (CIT), leading to loss or inactivation of the citron kinase protein (CITK), cause primary microcephaly in humans and rodents. This disorder is associated with cytokinesis failure and apoptosis in neural progenitors. It has therefore been postulated that the apoptosis observed after CITK loss is a consequence of impaired cytokinesis. However, studies performed in many different models indicate that cytokinesis failure leads more frequently to cell cycle arrest than apoptosis, suggesting that another fundamental event must occur. Using CIT ko and kinase inactive mice models compared to forebrain organoids derived from CIT mutated patients iPSCs, we found that CITK inactivation induces DNA damage accumulation and chromosomal instability in human and mouse neural progenitors. Moreover, recruitment of RAD51 to DNA damage foci is compromised by CITK loss or inactivation, indicating that CITK is involved in homologous recombination. Despite the same molecular lesion in ko and kinase inactive mutations in neural progenitors, different amounts of damage in kinase inactive mutations generate a syndrome with less severity compared to ko. This suggests that there is a level of damage capable to induce a precise apoptosis in neural progenitors that can represent a common thread between unrelated microcephaly syndromes.

## 3. Rescuing Neural Cell Survival and Maturation in a Primary Autosomal Recessive Microcephaly-17 (MCPH17) Mouse Model: Effects of the Postnatal N-Acetyl Cysteine Treatment

Maryam Khastkhodaei Ardakani ^1^, Cecilia Astigiano ^1^, Francesco Ferrini ^2^, Chiara La Rosa ^3^, Roberta Schellino ^1^, Marina Boido ^1^, Serena Bovetti ^3^, Annalisa Buffo ^1^ and Enrica Boda ^1^

^1^  Dept. of Neuroscience and Neuroscience Institute Cavalieri Ottolenghi, University of Turin, Turin, Italy

^2^  Dept. of Veterinary Sciences, University of Turin, Turin, Italy

^3^  Dept. of Life Sciences and Systems Biology and Neuroscience Institute Cavalieri Ottolenghi, University of Turin, Turin, Italy

Microcephaly 17 (MCPH17) is a rare neurodevelopmental disorder caused by mutations in the CIT gene, which encodes for the Citron Kinase (CIT-K) protein involved in DNA repair and cytoskeletal dynamics. Patients show reduced brain volume, simplified gyrification, intellectual disability, motor deficits, epilepsy, and early mortality. Cit-k KO mice recapitulate the human MCPH17 phenotype and shows epilepsy, ataxia and early lethality, DNA damage and reactive oxygen species (ROS) accumulation, apoptosis and maturation defects in neuronal and glial progenitors, and microglia increase. With the aim to identify pharmacological treatments that can reduce the cellular damage accumulation and improve the functional and histopathological phenotype of Cit-k KO mice, we performed a chronic treatment during the first two postnatal weeks with the antioxidant drug N-acetylcysteine (NAC), which is already FDA-/EMA-approved and can pass the blood–brain barrier. NAC treatment reduces brain ROS levels and slightly increases Cit-k KO mouse life span. Nevertheless, treated mice show a significant improvement in motor performance and reduction in myoclonus. Major neuroanatomical defects and a reduction of cortical interneurons persisted in the treated Cit-k KO mice. Yet, cortical oligodendrocyte progenitors and astrocytes significantly increased in numbers, while microglia density and morphology were largely normalized by NAC treatment. Interestingly, the deposition of perineuronal nets around cortical interneurons was also significantly rescued by NAC treatment, suggesting the promotion of interneuron maturation. In the periphery, NAC promotes the maturation of the neuromuscular junctions, possibly underlying part of the rescue of Cit-k KO mouse motor phenotype. Patch-clamp recordings and in vivo calcium imaging analyses in the cerebral cortex are ongoing to unveil the functional bases of NAC effects. Our data suggest that NAC postnatal treatment may be beneficial for the treatment of MCPH17.

## 4. The pH-Sensing Receptor TDAG8 Modulates Inflammatory Signalling and Maturation of Oligodendrocytes

Mikolaj Opielka ^1^, Fionä Caratis ^2^, Martin Hausmann Hausmann ^3^, Gerhard Rogler ^3^, Bartosz Karaszewski ^4^ and Aleksandra Rutkowska ^2^

^1^  Medical University of Gdansk, Department of Adult Neurology, Gdansk, Poland

^2^  Department of Anatomy and Neurobiology, Medical University of Gdansk, Gdansk, Poland

^3^  University Hospital Zürich, Division of Gastroenterology and Hepatology, Zurich, Switzerland

^4^  Medical University of Gdansk & University Clinical Center, Department of Adult Neurology, Gdansk, Poland

Acidosis is one of the hallmarks of demyelinating central nervous system (CNS) lesions in multiple sclerosis (MS). The response to acidic pH is mediated by a family of G protein-coupled proton-sensing receptors, including OGR1, GPR4, and TDAG8. These receptors remain inactive at alkaline pH, while at acidic pH~6.5 they are maximally activated. Their recently discovered functions include the regulation of inflammation and immune responses, modulation of hypoxic/ischemic environment, and tumorigenesis. In particular, TDAG8, which is highly expressed in the immune cells, is a negative regulator of inflammation. Its immunomodulatory effects depend mainly on Gαs signaling and cyclic AMP accumulation. Moreover, genome-wide association studies identified the TDAG8 locus to be associated with several autoimmune diseases including MS. Notably, we found that TDAG8 is upregulated in demyelinating plaques and the peri-plaque regions and downregulated in the white matter of MS patients. In the animal model of MS, the experimental autoimmune encephalomyelitis, TDAG8-deficient mice develop an exacerbated course of the disease. Interestingly, we found that pH-sensing receptors are in disequilibrium in TDAG8 knock-out mice. In the absence of TDAG8, the CNS expression levels of OGR1 and GPR4 are upregulated, thus changing the balance towards pro-inflammatory signaling. We also demonstrated that TDAG8-mediated signaling is involved in MO3.13 oligodendrocyte migration and maturation. In acidic pH, oligodendrocytes upregulate TDAG8 and cease to mature and differentiate. Moreover, the treatment of MO3.13 oligodendrocytes with TDAG8 agonist, tetrahydropalmatine, decreased the levels of pro-inflammatory cytokines after challenge with bacterial lipopolysaccharide. Together, these findings elucidate the role of TDAG8 in oligodendrocyte biology and indicate it might play a role in the pathophysiology of MS.

## 5. DNA Methylation Profiling of Patients with Aicardi-Goutières Syndrome Carrying the Identical p.A177T RNASEH2B Mutation but Showing Heterogeneous Phenotypes

Jessica Garau ^1^, Amandine Charras ^2^, Simona Orcesi ^3^, Costanza Varesio ^3^, Francesca Dragoni ^4^, Stella Gagliardi ^5^, Orietta Pansarasa ^6^, Cristina Cereda ^7^ and Christian Hedrich ^2^

^1^  Neurogenetics Research Centre, IRCCS Mondino Foundation, Pavia, Italy

^2^  Department of Women’s and Children’s Health, Institute of Life Course and Medical Sciences, University of Liverpool, Liverpool, UK

^3^  Department of Brain and Behavioral Sciences, University of Pavia, Pavia, Italy

^4^  Department of Biology and Biotechnology, University of Pavia, Pavia, Italy

^5^  Molecular Biology and Transcriptomics Unit, IRCCS Mondino Foundation, Pavia, Italy

^6^  Cellular Model and Neuroepigenetics Unit, IRCCS Mondino Foundation, Pavia, Italy

^7^  Department of Women, Mothers and Neonatal Care, Children’s Hospital “V. Buzzi”, Milan, Italy

Aicardi-Goutières Syndrome (AGS) is a rare, genetically mediated pediatric inflammatory disease characterized by the overexpression of interferon-stimulated genes (ISGs) and cerebral abnormalities. Patients with mutations in some AGS-related genes (TREX1, RNASEH2A, RNASEH2B, SAMHD1) may exhibit variable clinical phenotypes although carrying the same mutation. Currently, the severity of the disease can only be assessed through clinical evaluation and no biomarkers are available to predict disease severity or progression. The RNASEH2B p.A177T mutation is the most common variant observed in our cohort of AGS patients. It is associated with variable clinical phenotypes that range from “severe”, devastating neuro-inflammatory disease to “mild” courses with late onset.

To identify molecular signatures that correlate with disease severity, we performed DNA methylation profiling in peripheral blood cells from AGS patients with “mild” or “severe” disease who carry the same RNASEH2B p.A177T mutation and heathy controls.

When compared to controls, AGS patients presented hypomethylation of ISGs and differential methylation patterns in genes involved in neutrophil and platelet activation. Patients with “mild” phenotypes exhibited DMPs in genes involved in DNA damage and repair, whereas patients with “severe” phenotypes had different methylation profiles in genes involved in cell fate commitment and organ development. We also found hypomethylated positions in two key ISGs (IFI44L, RSAD2) associated with gene overexpression in patients with “severe” when compared to “mild” AGS phenotypes (qRT-PCR). Based on this observation, a “reduced” interferon score, consisting of IFI44L and RSAD2, may aid in discriminating “mild” from “severe” phenotypes in AGS patients carrying the RNASEH2B p.A177T mutation.

Taken together, this project delivered predictive biomarker candidates that may allow the evaluation of disease severity and prediction of progression to guide therapeutic decisions in AGS.

## 6. Anti-NG2 Autoantibodies as Prognostic Biomarker in Persons with Multiple Sclerosis

Marta Bottero ^1^, Giada Pessina ^2^, Fabrizio Loiacono ^3^, Matilde Inglese ^4^, Diego Franciotta ^4^, Nicole Kerlero de Rosbo ^1^, Antonio Uccelli ^1^ and Giovanni Ferrara ^1^

^1^  IRCCS Policlinico San Martino, Experimental Neuroscience, Genoa, Italy

^2^  University of Genoa, Dinogmi, Genoa, Italy

^3^  IRCCS Policlinico San Martino, Immunology, Genoa, Italy

^4^  IRCCS Policlinico San Martino, Neurology, Genoa, Italy

Nerve glial antigen 2 (NG2) is a marker of oligodendrocyte progenitor cells (OPC) and pericytes and is also expressed by murine immune cells like dendritic cells, T cells, and macrophages. OPCs are precursors of oligodendrocytes while pericytes are essential components of the neurovascular unit. In multiple sclerosis (MS), an inflammatory, demyelinating disease of the central nervous system (CNS), remyelination occurs early in the disease but fails at later stages. While remyelination failure is not fully understood, OPCs are targets of the disease, affecting their recruitment and/or their differentiation. During neuroinflammation, NG2 is processed by metalloproteases, and its extracellular portion is deposited in the CNS parenchyma. We hypothesized that NG2 could be a target of the immune system, because the soluble peptides of NG2 in the CNS could trigger a response. Accordingly, the aim of this project is to understand if anti-NG2 antibodies (αNG2) are present in the cerebrospinal fluid (CSF) of MS persons and to understand their role. We analyzed CSF from 114 MS persons and from 108 persons with other neurological diseases (OND), as controls. We found that αNG2 were present in 32% of the CSF of MS persons (MS+) who also showed a higher disease progression index, suggesting a possible role for αNG2 as prognostic biomarker. Immunofluorescence experiments confirmed that MS+ αNG2 stained OPCs and we identified the laminin G-like domain as containing an epitope recognized by MS+ αNG2. We found that MS+ CSF induced the activation of Caspase-8 upon complement activation and flow cytometry experiments showed that OPC death was increased in OPCs exposed to MS+ CSF as compared to control CSF. In conclusion, we suggest that αNG2 recognizing a specific epitopic region are elevated in a group of MS persons, and that those antibodies might play a role in OPC death or impair their differentiation and should be further studied as a possible adjunct tool for MS prognosis.

## 7. Effect of Maternal Butyrate Supplement on Autistic-like Behavior and Synaptic Plasticity Deficits in Mice Offspring

Claudia Cristiano ^1^, Eriola Hoxha ^2^, Pellegrino Lippiello ^1^, Roberto Russo ^1^, Filippo Tempia ^2^ and Maria Concetta Miniaci ^1^

^1^  University of Naples Federico II, Department of Pharmacy, Naples, Italy

^2^  University of Turin, Department of Neuroscience, Turin, Italy

Several studies have demonstrated a relationship between the alteration of maternal gut microbiota and an increased risk of neurodevelopmental disorders in offspring, including autism spectrum disorders (ASD). Among the microbiota-derived metabolites, butyrate (BUT) is a short-chain fatty acid (SCFA) produced in the colon by bacterial fermentation of dietary fibers which, in addition to its local effects, has neuroactive properties influencing neurological and behavioral processes. Indeed, BUT attenuated social deficits in an ASD mouse model and its levels are low in ASD subjects. However, the idea of compensating such metabolic dysfunction at a very early stage of disease via maternal treatment has not been sufficiently explored and much less is known about the cellular mechanisms on the brain physiology and behavior in ASD.

For our study, we used an inbred BTBR T+Itpr3tf/J (BTBR) mouse strain and we treated dams with BUT from mating to weaning. We analyzed behavioral and synaptic plasticity deficits in the offspring during juvenile and adult life, focusing on the cerebellum.

Our results show that the BUT treatment of BTBR dams prevents the social deficit and partly reduces the repetitive behavior in the offspring and prevents the hypertrophy of the cerebellar molecular and granular layers in the BTBR offspring compared to untreated mice. This effect was accompanied by a rescue of Purkinje cells (PC) firing and long-term synaptic plasticity deficits involving the parallel fiber-PC synapse.

In conclusion, our results show for the first time how the early treatment with a gut microbiota metabolite such as BUT prevents the development of ASD in mice offspring, supporting the hypothesis that the gut–brain axis plays an important role in the pathogenesis of ASD.

## 8. Mechanoreception in Glioma: An Insight into the Role of Piezo1 in GBM Progression and Cancer Stem Cells

Maria Velasco-Estevez ^1^, Alvaro Otero-Sobrino ^1^, Miguel Ángel Navarro-Aguadero ^1^, Pedro Aguilar-Garrido ^1^, Maria Hernandez-Sanchez ^2^ and Miguel Gallardo ^1^

^1^  Centro Nacional de Investigaciones Oncologicas (CNIO), Clinical Research Unit, Madrid, Spain

^2^  Universidad Complutense de Madrid (UCM), Department de Bioquimica y Biologia Molcular, Facultad de Farmacia, Madrid, Spain

Glioblastoma multiforme (GBM) is the most aggressive brain tumor in adults, affecting 2–3 per 100,000 adults/year. Current treatments include surgery, radiotherapy, and chemotherapy. However, prognosis is not optimistic, with survival rate of 14–18 months, and with only 10% of patients living up to five years after diagnosis. This is partly due to the existence of glioma ‘cancer stem cells’ (CSC), a subtype of tumor-initiating cells with stem cell-like properties and resistant to conventional treatments, being the cause of most relapses. Historically, researchers have focused on the biochemical and genetic aspects of cancer. However, in the last decade, the importance of mechanoreception has been evident. It is known that the microenvironment of tumors is very stiff, and that tumor cells overexpress mechanoreceptor proteins able to respond to these changes. Amongst them, we can find Piezo1, a calcium channel described in 2010, implicated in multiple cellular processes, such as migration or apoptosis, and which seems to be altered in various cancers. In this work, we explore the role of Piezo1 in GBM, as we hypothesize that Piezo1 contributes to the progression and malignancy of GBM by the formation of CSC. Here, we have observed that the chemical activation of Piezo1 in IPSC led to the upregulation of stem marker Oct3/4. Likewise, in a colony formation assay using human stem cells grown in methylcellulose, we observed that the activation of Piezo1 prevented differentiation, while inhibition had the opposite effect, suggesting that Piezo1 has an effect on stem-like phenotype maintenance. Furthermore, we have modified the U251 cell line to have Piezo1-KO and Piezo1-overexpressing cells (via CRISPR-Cas9 and CRISPR-SAM, respectively) and analyzed their viability, colony formation capacity, migration, and cell cycle arrest. Finally, we are developing a Tg.Piezo1/GFAP-cre mouse model to explore the effects of Piezo1 overexpression in glial cells in vivo in terms of neuroinflammation and tumor development.

## 9. Histone-Deacetylase 8 Drives the Immune Response and the Growth of Glioma

Alessandro Mormino ^1^, Germana Cocozza ^2^, Giulia Fontemaggi ^3^, Sergio Valente ^4^, Vincenzo Esposito ^5^, Antonio Santoro ^6^, Giovanni Bernardini ^7^, Angela Santoni ^8^, Francesco Fazi ^9^, Antonello Mai ^4^, Cristina Limatola ^10^ and Stefano Garofalo ^11^

^1^  Sapienza, University of Rome, Department of Physiology and pharmacology, Rome, Italy

^2^  IRCCS Neuromed, Neurofisiologia-inflammaging, Pozzilli, Italy

^3^  “Regina Elena” National Cancer Institute–IFO, Oncogenomic and Epigenetic Unit, Rome, Italy

^4^  Sapienza, University of Rome, Department of Drug Chemistry and Technologies, Rome, Italy

^5^  IRCCS Neuromed, Neurochirurgia sperimentale, Pozzilli, Italy

^6^  Sapienza, university of Rome, Department of Neurology and Psychiatry, Rome, Italy

^7^  Sapienza University of Rome, Department of Molecular Medicine, Rome, Italy

^8^  IRCCS Neuromed, Inflammaging, Pozzilli, Italy

^9^  Sapienza, University of Rome, Department of Anatomical, Histological, Forensic & Orthopaedic Sciences, Section of Histology and Medical Embryology, Rome, Italy 

^10^  Sapienza, University of Rome, Department of Physiology and Pharmacology, Laboratory Affiliated to Istituto Pasteur Italia, Rome, Italy 

^11^  Sapienza, University of Rome, Department of Physiology of Pharmacology, Rome, Italy 

Many epigenetic modifications occur in glioma, in particular the histone-deacetylase class proteins play a pivotal role in glioma development, driving the proliferation rate and the invasiveness of tumor cells, and modulating the tumor microenvironment. In this study, we evaluated the role of the histone deacetylase HDAC8 in the regulation of the immune response in glioma and tumor growth. We found that the inhibition of HDAC8 by the specific inhibitor PCI-34051 reduces tumor volume in glioma mouse models. We reported that HDAC8 modulates the viability and the migration of human and murine glioma cells. Interestingly, HDAC8 inhibition increases the acetylation of alpha-tubulin, suggesting that this epigenetic modification controls glioma migration. Furthermore, we identify HDAC8 as a key molecule that supports a poorly immunogenic tumor microenvironment, modulating microglial phenotype and regulating the gene transcription of NKG2D ligands that trigger the natural killer cell-mediated cytotoxicity of tumor cells. Altogether, these results identify HDAC8 as a key actor in glioma growth and the tumor microenvironment and pave the way to a better knowledge of the molecular mechanisms of immune escape in glioma.

## 10. Investigating the Feasibility of Assessing Magnetization Transfer Properties of Distinct White-Matter Connections

Pietro Bontempi ^1^, Ilana Leppert ^2^, Simona Schiavi ^3^, Jennifer Campbell ^2^, Mark Nelson ^2^, Bruce Pike ^4^, Christine Tardif ^2^ and Alessandro Daducci ^1^

^1^  University of Verona, Department of Computer Science, Verona, Italy

^2^  McGill University, McConnel Brain Imaging Centre, Montreal, Canada

^3^  University of Genoa, Department of Neuroscience, Rehabilitation, Ophthalmology, Genetics, Maternal and Child Health, Genova, Italy

^4^  University of Calgary, Hotchkiss Brain Institute and Departments of Radiology and Clinical Neuroscience, Calgary, Canada

Magnetization transfer ratio (MTR) maps can be associated to the myelin content of the tissue: the higher the MTR, the higher the myelin content. However, in white matter regions where multiple fiber populations, i.e., bundles, can cross the same voxel, the MTR value is voxel- rather than bundle-specific. We propose a method that allows for the assessment of bundle-specific MTR by combining a co-encoded diffusion and MT weighted sequence with Convex Optimization Modeling for Microstructure Informed Tractography (COMMIT), a framework allowing for the estimation of bundle-specific tissue properties. Four healthy subjects (HS) were imaged with a T1w sequence and a novel MT-prepared diffusion-weighted (DW) sequence (MTon). An identical DW sequence, without MT-preparation, was also acquired (MToff). T1 images were segmented in 85 grey matter regions with FreeSurfer and registered to the DW data. A probabilistic tractogram was reconstructed from MToff data and the COMMIT model was then fitted to Mtoff and Mton data separately. Two connectomes, for the Mtoff and Mton data, were calculated by grouping streamlines connecting the same region pair. An MTR weighted connectome was subsequently calculated with element-wise operation on the two connectomes (MTR = (MToff − MTon)/MToff), thus allowing to calculate a bundle specific MTR value. The proposed method was compared to tractometry which, for each streamline in a specific bundle, averages the MTR values along the streamlines path. In all the four HS, in some representative bundles that belong to the left motor network, the MTR values estimated with COMMIT are higher for the bundles connecting the left precentral gyri (L-PrCG) with the medulla (which is a heavily myelinated bundle) than those that connect the L-PrCG with the left subcortical nuclei. In contrast, the tractometry approach appears flat. By applying COMMIT to an innovative dual-encoded MT-dMRI weighted sequence, it is possible to measure bundle-specific MTR.

## 11. Development of a Frontotemporal Dementia Computer-Aided Diagnostic Tool Using a Dense Convolutional Neural Network on 3D Brain Scans and Explainable Artificial Intelligence Methods

Andrea Termine ^1^, Carlo Fabrizio ^1^, Carlo Caltagirone ^2^ and Laura Petrosini ^3^

^1^  IRCCS Santa Lucia Foundation, Data Science Unit, Rome, Italy

^2^  IRCCS Santa Lucia Foundation, Department of Clinical and Behavioral Neurology, Rome, Italy

^3^  IRCCS Santa Lucia Foundation, Experimental and Behavioral Neurophysiology Laboratory, Rome, Italy

Despite artificial intelligence (AI) being a leading technology in biomedical research, real-life implementation of AI-based computer-aided diagnosis (CAD) tools into the clinical setting is still facing obstacles. In particular, CAD tools lack standardization practices, leading to poorly reproducible results. This heterogeneity in development is frequently associated with unexplainable results, as deep learning (DL) is often considered a “black box” AI technology. Here, we present the development of an easily reproducible and fully explainable CAD tool using the Clinica and MONAI frameworks and the explainable AI methods (XAI). In particular, a deep learning (DL) convolutional neural network was trained to detect frontotemporal dementia (FTD) on 3D neuroimages from the NIFD database to ensure reproducibility. The DL pipeline includes the preprocessing and the augmenting steps of the 3D images, as well as hold-out cross-validation. The DL convolutional neural network (CNN) achieved a performance comparable to other FTD classification approaches, yielding 0.80 accuracy (95% confidence intervals: 0.64, 0.91), 1 sensitivity, 0.6 specificity, an F1-score of 0.83, and an AUC of 0.86, while maintaining full replicability. XAI methods were applied to understand AI diagnostic behavior and to identify regions of the images where the CNN misbehaves. Specifically, attention maps highlighted that the CNN decision was driven by hallmarking brain areas for FTD, which helped us to understand how to improve FTD detection. AI-based CAD tools should be developed with the goal of standardizing pipelines, as varying pre-processing and training methods, along with the absence of model behavior explanations, negatively impact regulators’ attitudes towards CAD. The adoption of common best practices for neuroimaging data analysis is a step toward the fast evaluation of the efficacy and safety of CAD and may further accelerate the adoption of AI products in the healthcare system.

## 12. Optimization of AAV9 Gene Therapy for Spinal Muscular Atrophy with Respiratory Distress Type 1 Using In Vivo Disease Model

Elisa Pagliari ^1^, Alessia Anastasia ^2^, Manuela Garbellini ^2^, Floriana Bellandi ^1^, Paola Rinchetti ^1^, Valentina Melzi ^2^, Michela Taiana ^1^, Giacomo Pietro Comi ^1^, Stefania Corti ^1^ and Monica Nizzardo ^1^

^1^  Università degli Studi di Milano, Department of Pathophysiology and Transplantation, Milan, Italy

^2^  Fondazione IRCCS Ca’ Granda Ospedale Maggiore Policlinico, Neurology Unit, Milan, Italy

Spinal muscular atrophy with respiratory distress type 1 (SMARD1) is a rare autosomal recessive motoneuron disease with infantile onset. It is caused by mutations in the immunoglobulin mu-binding protein 2 (IGHMBP2) gene, leading to a deficient amount of the encoded protein. The main clinical symptoms are distal muscular atrophy and diaphragmatic palsy. In this work, we compared the efficiency of two AAV9-IGHMBP2 vectors, different for promoters, by administering them intracerebroventricularly in a presymptomatic SMARD1 mouse model at postnatal day 1 (p1). The selected best construct was then tested in already symptomatic mice at p7 by systemic subcutaneous injection, to define the therapeutic window and the best route of administration. Expression analysis at p20 on mice treated during the pre- and symptomatic phase of the disease demonstrated a significant increase in the IGHMBP2 protein expression level and resulted in an extended survival time, higher body weight, and improvement in motor behaviors. In particular, p1 treated mice showed an increased innervation of the neuromuscular junctions, recovery of muscles fiber diameter, and an increased number of motoneurons in the spinal cords associated with reduced gliosis. To support the translatability of the therapy, we confirmed the lack of a significant alteration in the toxicity biomarkers after the treatments, thus demonstrating the efficacy of gene therapy for SMARD1 in an in vivo model with a lack of relevant toxic effects. In addition, the preliminary results of the same analysis performed on a delayed mice cohort, treated systemically with the selected best construct, showed a similar outcome. The results obtained so far have contributed to paving the way for the first Phase I/IIa gene therapy clinical study for SMARD1 started in December 2021 at Nationwide Children’s Hospital, Columbus, Ohio, in parallel, defining the therapeutic window and the choicest administration route to optimize gene therapy strategy.

## 13. Combined RNA Interference and Gene Replacement Therapy Targeting MFN2 for the Treatment of Charcot-Marie-Tooth Type 2A

Elena Abati ^1^, Silvia Bono ^1^, Marc-David Ruepp ^2^, Sabrina Salani ^1^, Linda Ottoboni ^1^, Valentina Melzi ^1^, Serena Pagliarani ^3^, Roberta De Gioia ^1^, Alessia Anastasia ^1^, Michela Taiana ^1^, Simona Lodato ^4^, Paolo Kunderfranco ^4^, Nereo Bresolin ^3^, Giacomo Comi ^3^, Stefania Corti ^3^, Monica Nizzardo ^3^ and Federica Rizzo ^1^

^1^  Fondazione IRCCS Ospedale Maggiore Policlinico Ca Granda, Dino Ferrari Centre, Neuroscience Section, Milano, Italy

^2^  King’s College London, UK Dementia Research Institute Centre, Institute of Psychiatry, Psychology and Neuroscience, London, UK

^3^  Università degli Studi di Milano, Department of Pathophysiology and Transplantation, Milan, Italy

^4^  Humanitas University, Department of Biomedical Sciences, Rozzano, Italy

Introduction: Mitofusin-2 (MFN2) is an outer mitochondrial membrane protein essential for mitochondrial networking in most cells. Autosomal dominant mutations in the MFN2 gene cause Charcot-Marie-Tooth type 2A disease (CMT2A), a severe and disabling sensory-motor neuropathy impacting the entire nervous system. Here, we propose a novel potential therapeutic approach combining RNA interference (RNAi) and gene therapy, whereby mutant and wild-type MFN2 mRNA are inhibited by RNA interference (RNAi), while the wild-type protein is restored by overexpressing cDNA encoding functional MFN2 modified to be resistant to RNAi.

Methods: After obtaining induced pluripotent stem cells (iPSCs) from somatic cells of CMT2A patients, we targeted the MFN2 mutant allele with specific short hairpin RNAs (shRNAs) and simultaneously introduced a mutagenized MFN2 gene resistant to shRNA activity and encoding the native protein. We then differentiated iPSCs into spinal motor neurons (MNs) and analyzed the sub-cellular parameters previously found to be altered in a CMT2A in vitro model to assess the impact of our therapy. We then evaluated this strategy in vivo in the MitoCharc1 CMT2A transgenic mouse model after the cerebrospinal fluid (CSF) delivery of the constructs into newborn mice using adeno-associated virus 9 (AAV9).

Results: This approach significantly rescues the CMT2A MN phenotype in vitro, stabilizing the altered axonal mitochondrial distribution and correcting abnormal mitophagic processes. This strategy also allows proper MFN2 molecular correction in CMT2A MitoCharc1 mice. Conclusions: Overall, our results led to a significant level of rescue of disease phenotype in CMT2A MNs, suggesting that RNAi/gene therapy combined approach might represent a promising therapeutic strategy for the broad spectrum of human diseases associated with MFN2 mutations.

## 14. Exploiting Three-Dimensional In Vitro Models to Identify Early Neuronal Vulnerability and Test Therapeutic Strategies in Amyotrophic Lateral Sclerosis

Delia Gagliardi ^1,2^, Andrea D’Angelo ^2^, Paola Rinchetti ^1^, Chiara Cordiglieri ^3^, Giacomo Pietro Comi ^1,4^, Monica Nizzardo ^2^ and Stefania Corti ^1,2^

^1^  Dino Ferrari Centre, Department of Pathophysiology and Transplantation (DEPT), University of Milan, 20122 Milan, Italy

^2^  Neurology Unit, Fondazione IRCCS Ca’ Granda Ospedale Maggiore Policlinico, 20122 Milan, Italy

^3^  National Institute of Molecular Genetics ‘Romeo e Enrica Invernizzi’, INGM, Milan, Italy

^4^  Neuromuscular and Rare Diseases Unit, Department of Neuroscience, Fondazione IRCCS Ca’ Granda Ospedale Maggiore Policlinico, 20122 Milan, Italy

Amyotrophic lateral sclerosis (ALS) is a fatal neurodegenerative disorder affecting motor neurons. Development of ALS therapeutics is hampered by incomplete knowledge of pathogenic mechanisms and lack of reliable disease models. C9ORF72, whose hexanucleotide repeat expansion (HRE) represents the main genetic cause of ALS, has been postulated to play a role in neurodevelopment. To investigate whether early developmental vulnerability in ALS could result in late onset neurodegeneration, we will exploit 3D patient-specific in vitro models of central nervous system (CNS).

We generated induced pluripotent stem cell (iPSC)-derived brain (BrOs) and spinal cord (ScOs) organoids of C9ORF72-ALS patients and isogenic controls, using a free-floating 3D-culture method based on aggregation in embryo bodies, embedment in matrigel, and agitation in a spinning bioreactor. Organoids were characterized by immunohistochemistry, Western blot, and transcriptomics analysis. Further, to assess the presence of a neural activity, we performed calcium imaging. Finally, we treated BrOs with an antisense oligonucleotide targeting C9ORF72-HRE.

BrOs and ScOs expressed pluripotency markers and mature neuronal markers in early and late stages, respectively. ALS organoids presented a higher rate of cell death and a lower degree of maturity compared to isogenic controls. C9ORF72-ALS organoids recapitulated disease hallmarks, like TDP-43 cytoplasmic mislocalization, and displayed a disruption of key cellular processes, e.g., DNA damage response and axonal elongation. Functional studies showed an increased calcium influx in C9ORF72-ALS BrOs and an increased susceptibility to glutamate stimulation in C9-ALS ScOs, compared to isogenic controls, suggesting neuronal overexcitability in ALS.

Patient-specific iPSC-derived 3D CNS models reproduce at different time points the maturation of neural and glial cells, resembling physiologic human neurodevelopment. BrOs and ScOs are valuable tools for disease modeling since they improve the characterization of C9ORF72-ALS pathology, dissecting specific disease hallmarks, and providing the opportunity to test therapeutic strategies.

## 15. New Insights into the Effects of SARS-CoV-2 Infection on Nervous System: Alteration of Dopamine Metabolism in iPSCs-Derived Dopaminergic Neurons

Giulia Lunghi ^1^, Emma Veronica Carsana ^1^, Silvia Breviario ^1^, Gioia Cappelletti ^2^, Claudio Fenizia ^2^ and Massimo Aureli ^1^

^1^  University of Milano, Department of Medical Biotechnology and Translational Medicine, Segrate (MI), Italy

^2^  University of Milano, Department of Biomedical and Clinical Sciences “L. Sacco”, Milano, Italy

Increasing evidence related to the onset of neurological symptoms is emerging from a high proportion of patients affected by COVID-19 pathology, suggesting the possible neuroinvasiveness of SARS-CoV-2. Recent studies show that an increasing number of patients, even with mild COVID-19, experience symptoms even weeks or months after the infection. These symptoms comprise a wide range of neurological conditions, such as memory and cognitive dysfunction, brain fog, headaches, insomnia, balance and speech issues, anxiety, and depression.

These premises suggest that SARS-CoV-2 infection is not restricted to the respiratory system, but also reaches the central nervous system. Particularly, in light of the COVID-19-related symptomatology, it has been hypothesized that SARS-CoV-2 might affect dopaminergic neurons. However, no scientific evidence has been produced so far.

To investigate this aspect, human iPSCs were differentiated into dopaminergic neurons and infected with three different SARS-CoV-2 variants (EU, Delta, and Omicron). The infection with EU and Delta variants, but not with Omicron, was responsible for a reduced intracellular content and extracellular release of dopamine. Moreover, neurons infected with EU and Delta SARS-CoV-2 were characterized by a reduced protein levels of Tyrosine hydroxylase together with a reduced mRNA expression of DOPA-decarboxylase and dopamine transporter, and an increase in VMAT2 transporter. In addition, the infected neurons displayed the onset of neurodegeneration, demonstrated by the reduction in MAP2 and TAU content. Finally, we found an intense activation of antiviral intracellular innate response and an increase in neuronal stress markers.

Taken together, these preliminary observations let us speculate that neurons are affected by SARS-CoV-2 infection, with particular consequences on dopamine production and metabolism, explaining some of the neurological symptoms developed upon SARS-CoV-2 infection.

## 16. Aberrant Protein Palmitoylation: A Novel Therapeutic Target in Alzheimer’s Disease

Francesca Natale ^1^, Matteo Spinelli ^1^, Walter Gulisano ^2^, Marco Rinaudo ^1^, Francesco La Greca ^3^, Daniela Puzzo ^2^, Salvatore Fusco ^1^ and Claudio Grassi ^1^

^1^  Università Cattolica del Sacro Cuore, Fondazione Policlinico Universitario A. Gemelli IRCCS, Department of Neuroscience, Rome, Italy

^2^  University of Catania, Department of Biomedical and Biotechnological Sciences, Catania, Italy

^3^  Università Cattolica del Sacro Cuore, Department of Neuroscience, Rome, Italy

Metabolic alterations may play a critical role in Alzheimer’s disease (AD) pathogenesis and progression. Our previous findings highlighted how brain insulin resistance (BIR) leads to memory impairment by impinging on protein palmitoylation, a posttranslational modification regulating neuronal protein localization and synaptic function. To begin, we analyzed 3xTg-AD mice brains and found high levels of AKT, GSK3β, and IRS-1 proteins phosphorylation, which is recognized as a molecular hallmark of BIR. Then, we analyzed the levels of palmitoylation of different proteins involved in synaptic function and plasticity in the hippocampus of 9-month-old 3xTg-AD mice through an acyl-biotin exchange assay. We found hyper-palmitoylation of several proteins compared to wild-type controls. Subsequently, we tested the effect of chronic intranasal administration of the palmitoylation inhibitor 2-bromopalmitate on both male and female 3xTg-AD mice by performing behavioral (novel object recognition and object displacement tests), electrophysiological (long-term potentiation, LTP), and molecular analyses (ELISA, immunofluorescence, Western blot). 2-bromopalmitate delayed the onset of memory deficits and significantly enhanced cognitive performances in six-, nine- and 12-month-old mice. Accordingly, electrophysiological analyses on hippocampal brain slices from 2-bromopalmitate-treated 3xTg-AD mice revealed greater LTP at CA3-CA1 synapses (LTP slope: 137.55 ± 10.82% vs. 225.7 ± 9.71%). In addition, 2-bromopalmitate reduced Aβ deposition in the hippocampus of both males and females (–45%/–60%, respectively). Taken together, our data suggest that aberrant palmitoylation plays a critical role in the onset and progression of AD. This study also represents the first preclinical study on the effects of 2-bromopalmitate on AD-related cognitive decline.

## 17. The Emerging Role of microRNAs in Experimental and Clinical Multiple Sclerosis: Implications for Inflammation-Driven Synaptic Dysfunctions and Disease Course

Francesca De Vito ^1^, Alessandra Musella ^2,3^, Francesca Romana Rizzo ^4^, Sara Balletta ^4^, Deigo Fresegna ^4^, Antonietta Gentile ^3^, Livia Guadalupi ^3,4^, Mario Stampanoni Bassi ^1^, Luana Glilio ^1^, Valerio Licursi ^5^, Alessandro Moscatelli ^6^, Colleen Patricia Ryan ^6^, Fabio Buttari ^1^, Silvia Caioli ^1^, Valentina Vanni ^3^, Krizia Sanna ^4^, Antonio Bruno ^1^, Ettore Dolcetti ^1^, Annamaria Finardi ^7^, Roberto Furlan ^7^, Diego Centonze ^1^ and Georgia Mandolesi ^2,3^

^1^  Unit of Neurology, IRCCS INM-Neuromed, Pozzilli (Is), Italy

^2^  Department of Human Sciences and Quality of Life Promotion, San Raffaele University of Rome, Roma, Italy

^3^  Synaptic Immunopathology Lab, IRCCS San Raffaele Pisana, Rome, Italy

^4^  Department of Systems Medicine, Tor Vergata University, Rome, Italy

^5^  Department of Biology and Biotechnologies “C. Darwin,” Laboratory of Functional Genomics and Proteomics of Model Systems, University of Rome “Sapienza”, Rome, Italy

^6^  Unit of Neuroimmunology, IRCCS, Fondazione Santa Lucia, 00143 Rome, Italy

^7^  Neuroimmunology Unit, Institute of Experimental Neurology (INSpe), Division of Neuroscience, San Raffaele Scientific Institute, Milan, Italy

MicroRNAs (miRs) are post-transcriptional regulators of gene expression which have recently come up as pleiotropic determinants in the crosstalk between central nervous system and immune system.

We investigated their role in the course of multiple sclerosis (MS) especially linked to the inflammatory synaptopathy, a crucial hallmark of the disease. Specifically, we screened, by qPCR and the Bio-plex system, 24 selected miRs and 27 inflammation-related proteins circulating cerebrospinal fluid (CSF) in a large cohort of MS patients, and we correlated them with clinical, cognitive, and transcranial magnetic stimulation parameters assessed at the diagnosis (T0) and after follow-up periods (Tf). Multiple statistical and bioinformatics analyses were also combined with preclinical studies on MOG35-55 EAE model and transgenic mice.

We identified let-7b-5p and miR-142-3p as two main miRs with opposite functions in neuroinflammation and MS prognosis. Let-7b-5p was a potential protective factor for MS course, with anti-inflammatory and neuroprotective properties from the earliest stages of the disease. Moreover, CSF let-7b-5p levels were reduced in progressive MS and negatively correlated with disease severity at T0 and Tf. On the contrary, miR-142-3p emerged as an adverse biomarker of the synaptopathy-driven disease progression and a promising tool for identifying personalized therapies. Indeed, we demonstrated in MS and in EAE that miR-142-3p was an essential effector of interleukin-1beta-induced synaptic alterations and low miR-142-3p levels associated with a more effective response to dimethyl fumarate, an established MS treatment.

Our results lay the basis for an important advance in MS diagnosis and prognosis related to synaptopathy-driven detrimental outcomes, with possible implications in the therapeutic decision-making strategy.

## 18. New Insight for Riboflavin Transporter Deficiency (RTD) Syndrome: Gene Therapy as a New Therapeutic Strategy for RTD Patients

Cecilia Mei ^1^, Valentina Magliocca ^2^, Monica Nizzardo ^1^, Xin Chen ^3^, Steven Gray ^3^, Marco Tartaglia ^2^, Enrico Silvio Bertini ^2^, Stefania Paola Corti ^1^ and Claudia Compagnucci ^2^

^1^  Università degli studi di Milano, Department of Pathophysiology and Transplantation, Milan, Italy

^2^  Ospedale pediatrico Bambino Gesù, Genetics and Rare Diseases Research Division, Rome, Italy

^3^  UTSW Medical Center, Department of Pediatrics, Dallas, TX, USA

RTD is a rare childhood-onset disorder caused by mutations in SLC52A3 and SLC52A2 genes, encoding the riboflavin (RF) transporters RFT2 and RFT3, respectively. Since RF is a precursor of flavin mononucleotide and flavin adenine dinucleotide, the reduction of its intracellular availability compromises several vital processes. Even if empirical studies reported clinical improvement with the administration of large dose of RF, it cannot be considered as an effective cure because some patients do not benefit from RF supplementation. The main goal of this project is to explore a new therapeutic approach using gene therapy with adeno-associated viral vector serotype 9 (AAV9) carrying human codon-optimized SLC52A2 cDNA (AAV9-SLC52A2) to rescue the RTD neural phenotype.

Induced pluripotent stem cells (iPSCs) derived from the skin fibroblasts of healthy subjects and RTD patients with mutation in SLC52A2 gene were successfully differentiated into motor neurons (MNs). In order to establish the best experimental conditions to obtain the maximal rate of infection, we pretreated the MNs with several concentrations of sialidase, in combination with multiple infections of the AAV9-SLC52A2 vector. After fixing and staining the MNs for β III-tubulin, we confirmed the successful infection of the MNs by immunofluorescence analyses and we found the best efficiency of infection for the MNs. In addition, we examined the neurites length of infected and uninfected RTD MNs against the normal control MNs and we observed an increase in neurite length. Collectively, our results indicate that the AAV9-SLC52A2 vector generates promising rescue in derived MNs from RTD patients, which warrants further in vitro as well as in vivo studies to develop the gene therapy as a potential treatment.

## 19. Central Effects of Botulinum Toxin Type A in Motor Nervous System of the Rat

Petra Šoštarić, Magdalena Matić and Ivica Matak

School of Medicine, University of Zagreb, Department of Pharmacology, Zagreb, Croatia

Botulinum toxin type A (BoNT-A) is a potent neurotoxin with anticholinergic effect. It is a standard therapy in various movement disorders, presumably due to action on local neuromuscular terminals. However, observations in clinics and recent experimental data point to the possible central effects. The aim was to examine the contribution of the transcytosis-dependent central toxin action on the long--term muscular function recovery in rats, as well as tetanus neurotoxin (TeNT) evoked spastic paralysis after peripheral application. Rats were bilaterally injected with BoNT-A into the gastrocnemius muscle (2 U/kg) or sciatic nerve (5 U/kg). To stop the toxin central transcytosis, BoNT-A-neutralizing antitoxin was intrathecally (i.t.) administered after 24 h. After recovery from flaccid paralysis, TeNT was intramuscularly (i.m.) injected to animals on day 62. In different motor tests (gait ability score, digit abduction score, rota-rod, beam walking and swimming performance), i.t. antitoxin significantly accelerated the flaccid paralysis and motor performance recovery. TeNT-evoked increase in muscle tone was reduced by BoNT-A dependently on its central effect. However, the H-reflex, when corrected for reduced muscle size or reduced compound muscle action potential (CMAP), was not affected by the toxin treatment, suggestive of the lack of the toxin’s direct effect on monosynaptic reflex. The toxin enzymatic activity examined by cleaved synaptosomal-associated protein 25 (cSNAP-25) immunohistochemistry, was still present in neuromuscular junctions and spinal cord. cSNAP-25, presence in second order spinal cord cholinergic neurons, depended on the toxin’s central transcytosis. Conclusion: Long term motor effects of BoNT-A both on normal motor performance (day 1–62), as well as the spastic paralysis (days 62–78), are influenced by the toxin’s ongoing central action mediated by retrograde transport and transcytosis. These data suggest that clinically relevant beneficial effects of BoNT-A result from the toxin’s combined peripheral and central effects.

## 20. Driving CARs on a Highway to Cure Pediatric CNS Malignant Tumors

Marta Ibáñez Navarro ^1^, Adrian Fernández Martín ^1^, Carmen Cano Torras ^1^, Laura Clares Villa ^2^, Cristina Ferreras ^2^, Antonio Pérez Martínez ^2^ and Lucía Fernández Casanova ^1^

^1^  Spanish National Cancer Research Centre, Hematological Malignances H120- Clinical Research Department, Madrid, Spain

^2^  Hospital La Paz Institute for Health Research- IdiPAZ, Translational Research in Paediatric Oncology, Haematopoietic Transplantation and Cell Therapy, Madrid, Spain

Pediatric central nervous system (CNS) malignant tumors are the most common solid tumors and the leading cause of cancer-related mortality in children. Although current treatments have resulted in prolonged free survival rates (60–70%), in those patients with relapse or metastases, the prognosis is very poor. In addition, the effects of surgery, chemo- and radiotherapy in the developing brain of children may involve irreversible neurological effects, underlying the urgent need to find more specific and less toxic treatments. In this regard, T cells expressing a chimeric antigen receptor (CAR T) are a promising therapeutic strategy to treat brain tumors. The interaction between NKG2D receptor, expressed in Natural Killer (NK) cells and their ligands (NKG2DL), overexpressed in tumor cells, are essential for NK cell anti-tumor immunosurveillance. Additionally, NK cells can exert antibody-dependent-cell-cytotoxicity (ADCC) on antibody coated tumor cells through CD16 receptor. However, the use of NK cells in the clinical setting presents some limitations derived from their poor in vivo expansion and lack of memory, among others. In an aim to overcome these limitations, we have engineered T cells with three different CAR constructs: NKG2D, CD16 and NKG2D-CD16 and tested their ability to target CNS pediatric tumor cells in vitro, either alone or in combination with Dinutuximab, a therapeutic IgG1 targeting GD2. We found that NKG2D CAR T cells were highly cytotoxic against different CNS tumors cell lines in 2D and 3D cultures. CD16 CAR and NKG2D-CD16 CAR T cells showed an antibody dose-dependent anti-tumor activity when combined with Dinutuximab. Importantly, the cytotoxicity of NKG2D-CD16 CAR T cells outperformed that exerted by CD16 CAR T cells, suggesting a potential synergistic effect. In conclusion, our preliminary results show that NKG2D, CD16, and NKG2D-CD16 CAR T cells target CNS tumor cells in vitro and could be a novel therapeutic approach to treat these tumors.

## 21. Human iPSC-Based Cellular Systems to Model Autosomal Dominant Leukodystrophy

Ingrid Battistella ^1^, Pietro Cortelli ^2^, Stefano Ratti ^3^, Lucia Manzoli ^3^, Pietro Guaraldi ^2^, Mariia Zadorozhna ^4^, Elisa Giorgio ^5^ and Luciano Conti ^1^

^1^  Laboratory of Stem Cell Biology, University of Trento, Department CIBIO, Trento, Italy

^2^  IRCCS Istituto delle Scienze Neurologiche di Bologna, Bellaria Hospital, Bologna, Italy

^3^  University of Bologna, Department of Biomedical and Neuromotor Sciences, Bologna, Italy

^4^  Laboratory of Medical Genetics, Department of Molecular Medicine, University of Pavia, Pavia, Italy

^5^  Medical Genetics Unit, IRCCS Fondazione Mondino, Pavia, Italy

Autosomal dominant leukodystrophy (ADLD) is a slow, progressive, genetic, and fatal neurological disorder. The genetic cause of ADLD is Lamin B1 (LMNB1) overexpression due to coding duplications or noncoding deletions at the LMNB1 locus. Lamin B1 is a component of the inner nuclear membrane of cells and although LMNB1 is ubiquitously expressed, it appears that neurons and glial cells are particularly sensible to LMNB1 dosage. Currently, only symptomatic and palliative treatments are available for this fatal disease. Since its discovery, human induced pluripotent stem cell (hiPSC) technology has supported the generation of novel and pathological-relevant in vitro models for central nervous system human diseases, for which no appropriate model systems were available. In this work, we describe the reprogramming of peripheral blood mononuclear cell and fibroblast lines derived from ADLD patients carrying different genetic mutations into hiPSCs by the Sendai virus-based method. These hiPSC lines were characterized to assess their pluripotency state by means of qRT-PCR and immunofluorescence assay. Moreover, embryoid bodies formation assay was used to evaluate their functional pluripotency. In parallel, we set up a procedure for the controlled differentiation of hiPSCs into oligodendrocytes, neurons, and astrocytes. These mature cells were characterized to assess the expression of stage-specific markers by means of qRT-PCR and immunofluorescence assays. In conclusion, patient-derived ADLD hiPSC lines coupled to the differentiation protocols that we report represent valuable tools for studies aiming to investigate ADLD-specific alterations at molecular and cellular levels and develop potential target specific drugs.

## 22. Glial Fibrillary Acid Protein Correlates with the Phenotype in Adult Patients with Tuberous Sclerosis Complex

Valeria Manzini ^1^, Biagio Orlando ^2^, Alessandra Morano ^2^, Emanuele Cerulli Irelli ^2^, Enrico Michele Salamone ^2^, Maria Sole Borioni ^2^, Anna Teresa Giallonardo ^2^, Elisa Moliterni ^3^, Sandra Giustini ^4^, Ivan Arisi ^5^, Chiara D’Amelio ^1^, Mara D’Onofrio ^1^, Caterina Veroni ^6^, Paola Piscopo ^6^ and Carlo Di Bonaventura ^2^

^1^  European Brain Reseaerch Institute (EBRI) “Rita Levi-Montalcini”, Genomics Facility, Rome, Italy

^2^  Sapienza University, Human Neurosciences Department, Rome, Italy

^3^  Sapienza University, Department of Odontostomatological and Maxillo-facial Sciences, Rome, Italy

^4^  Sapienza University, Department of Clinical, Internal, Anesthetic and Cardiovascular Sciences, Rome, Italy

^5^  European Brain Reseaerch Institute (EBRI) “Rita Levi-Montalcini”, Bioinformatics Facility, Rome, Italy

^6^  Istituto Superiore di Sanità, Department of Neurosciences, Rome, Italy

Tuberous sclerosis complex (TSC) is a rare dominant autosomal, neurocutaneous syndrome related to the hyperactivation of the mammalian Target of Rapamicine (mTOR) pathway, caused by mutation in one of the two genes, TSC1 or TSC2 (encoding for Hamartin and Tuberin). The mTOR pathway contributes to tau dysregulation, a process linked to the neurodegeneration and neuroinflammation and involved in the broad spectrum of clinical TSC phenotype. Among TSC neurological manifestations, epilepsy, intellectual disability, and psychiatric/behavioral disorders are of paramount importance. The aim of the study was to investigate correlations between biomarkers of neurodegeneration and neuroinflammation and TSC anatomo-clinical features. We hypothesized that molecular biomarkers reflecting inflammatory and neurodegenerative processes (Neurofibrillary Light Chain (NfL), Glial Fibrillary Acid Protein (GFAP), Aβ40, Aβ42, t-Tau and p-181 tau) could be differentially represented in peripheral blood. Clinical and radiological features were collected by reviewing clinical charts and brain MRI scans. We investigated plasma samples derived from 31 TSC patients versus 38 healthy controls using the Single Molecule Assay (Simoa™) technique and identified TSC1 and TSC2 mutation carriers and non-mutation identified (NMI). GFAP levels were increased in TSC patients, both in TSC1 and TSC2 mutation carriers. On the contrary, NMI showed a decreasing trend of GFA, which reached the significance when it was compared with TSC2 one. Plasma levels of NfL, Aβ40, Aβ42, and both total and p-181 Tau forms showed no-statistically significant differences. Interestingly, t-Tau levels became significant when we differentially analyzed the mutation carriers. Higher levels of GFAP were strongly associated with neurological symptoms and epileptic spasms. Our study documented a significant increase of GFAP levels in TSC adult patients, which appeared to be correlated with the severity of the neurological phenotype.

## 23. Prenatal Exposure to Poly I:C Induces Tissue-Specific Expression of Several ERVs and Related Genes, and Immune Effectors in Cortex, Hippocampus, and Blood Samples from C57BL/6 Mice

Martina Giudice ^1^, Chiara Cipriani ^2^, Erica D’Avorio ^2^, Anna Maria Tartaglione ^3^, Vita Petrone ^2^, Marialaura Fanelli ^2^, Christian Maracchioni ^2^, Rossella Chirico ^2^, Antonella Minutolo ^2^, Gemma Calamandrei ^3^, Claudia Matteucci ^2^, Laura Ricceri ^2^, Paola Sinibaldi Vallebona ^2^ and Emanuela Balestrieri ^2^

^1^  Dipartimento di Medicina Sperimentale, Università di Roma Tor Vergata, Roma, Italy 

^2^  Department of Experimental Medicine, University of Rome Tor Vergata, Roma, Italy 

^3^  Centre for Behavioural Sciences and Mental Health, Istituto Superiore di Sanità, Roma, Italy

Autism spectrum disorder (ASD) is a neurodevelopmental disorder resulting from complex interactions among genetic, environmental, and epigenetic factors. Human endogenous retroviruses (HERVs) are relics of ancestral germline infections by exogenous retroviruses, stably integrated into the host cellular DNA, which comprise about 8% of genome in human and over 10% in mice. HERVs deregulation has been associated with many complex human diseases, such as neurological and psychiatric disorders. Our previous study on two preclinical mouse models of ASD showed an altered expression of several ERVs and cytokines, in embryos, blood, and brain samples at different post-natal ages, supporting the potential involvement of ERVs and immune response in the pathophysiology of ASD. The aim of this work was to study the effect of prenatal exposure to viral mimetic analogous to a double-stranded RNA (Poly I:C) on the expression of several ERVs families, ERV-related genes, immune effectors, and marker of damage to the central nervous system (CNS), in different tissues of adult mice. C57BL/6J pregnant female mice were treated with a single injection of Poly I:C or saline solution at gestational day 12.5. Behavioral evaluation of the offspring and tissue collection (cortex, hippocampus, and blood samples) were performed on post-natal day 60. The analysis of the expression of several ERVs and related genes, proinflammatory and regulatory cytokines, toll-like receptors, and markers of CNS damage by quantitative real-time PCR analysis, showed that Poly I:C exposure results in tissue-specific deregulation of ERVs and inflammatory and regulatory cytokines, in parallel to the appearance of an autism-like phenotype in offspring. This supports the hypothesis of an interaction between HERVs activity and altered inflammatory response and their involvement in the biological mechanism underlying ASD.

## 24. The Effect of a Ketogenic Diet on the Host Microbiota, the Immune System and the CNS

Andrina Rutsch ^1^, Johan Björn Kantsjö ^1^, David Ferreira Francisco ^2^, Marco Kreuzer ^2^, Pamela Nicholson ^3^ and Francesca Ronchi ^1^

^1^  Charité Universtitätsmedizin, Institut für Mikrobiologie und Infektionsimmunologie, Berlin, Germany 

^2^  University of Bern, Bioinformatics and Computational Biology, Bern, Switzerland

^3^  NGS platform Bern, Institute of Genetics, Bern, Switzerland

The intestinal microbiota plays a fundamental role in host protection, metabolism, and in the function of host organs, including the central nervous system (CNS). Changes in the microbiota composition have been reported in several neurological disorders. The CNS and the intestine interact through a bidirectional network called the gut–brain axis, in which microbial metabolites are importantly involved, as well the immune system and nervous structures, e.g., the enteric nervous system and the vagus nerve. The microbiota is highly influenced by the dietary habits of the host. The ketogenic diet (KD) ameliorates conditions in metabolic and neurological diseases. However, the mechanisms behind this are not well known. Our aim is to understand the effects of KD on the function of the gut microbiota and subsequently the CNS under healthy and neurological disease conditions. We performed 16S rRNA sequencing and metatranscriptomics on the intestinal content of mice fed KD or a control diet, colonized with an undefined, (specific-pathogen free, SPF) or with a defined, microbiota (sDMDMm). KD increased the Firmicutes/Bacteroidetes ratio and induced important metabolic changes in Clostridia. To investigate the effect of KD on the immune- and nervous system, we analyzed intestinal- and brain immune cells by flow cytometry and the brain cells by spatial transcriptomics of mice fed KD or control diet in germ-free (GF) or SPF condition. In the brain, several genes were affected upon KD and most of them in a microbiota-dependent way. In general, KD impacts genes involved in neuronal development and regeneration in several brain regions. Additionally, KD induced a decrease in γδT cells in the brain of SPF mice compared to control groups. We are currently investigating if bacterial metabolites are responsible for the effects we observed in a KD-microbiota-dependent way. Overall, we are studying new mechanisms of action of diet and microbiota on immune cells and on brain function.

## 25. Gene Expression Profiling in Trigeminal Ganglia from Cntnap2-/- and Shank3b-/- Mouse Models of Autism Spectrum Disorder

Alessandra Ciancone Chama, Yuri Bozzi and Luigi Balasco

University of Trento, CIMEC, Rovereto, Italy

Tactile sensory deficits are commonly found in individuals diagnosed with autism spectrum disorder (ASD). For this reason, considerable research is currently dedicated to understanding the underlying basis of these disturbances, utilizing ASD animal models such as Shank3b-/- and Cntnap2-/- mice. Notwithstanding the existing body of work, a focus on the whisker system, which constitutes the dominant somatosensory pathway in mice, is lacking. The present study seeks to fill this knowledge gap by characterizing gene expression profiles in the trigeminal ganglia (TG) of Shank3b-/- and Cntnap2-/- mice. TG receive direct innervation from the whiskers, and therefore represent a crucial area for somatosensory input processing. mRNA expression of ASD markers within the TG of Shank3b-/- and Cntnap2-/- adult and juvenile mice relative to age-matched controls was analyzed using qRT-PCR. Results show a differential expression of key molecular markers in the TG, such as markers for inhibitory and excitatory neurotransmission, as well as neuroinflammatory molecules. Both knockout mice lines exhibit a dysregulation in Gad1 and Gfap gene expression throughout development. Therefore, it can be concluded that ASD Shank3b and Cntnap2 mutations influence the somatosensory whisker system, resulting in altered gene expression even at the level of first order sensory neurons. Such results suggest early dysregulation in synaptic signaling and neuroinflammation pathways, both of which have been strongly implicated in the neuropathology of ASD. These findings are crucial for promoting the development of novel peripherally targeted treatments for tactile sensory deficits exhibited in such neurodevelopmental disorders.

## 26. The Role of bdnf in Epilepsy: Evidence from a Pharmacological Zebrafish Model of Disease

Carmine Merola ^1^, Alessandro Piccinini ^1^, Giulia Caioni ^2^, Elisabetta Benedetti ^2^, Monia Perugini ^1^, Michele Amorena ^1^, Annamaria Iannetta ^1^, Clarissa Ciffolillo ^1^ and Claudio D’Addario ^1^

^1^  University of Teramo, Faculty of Bioscience and Technology for Food, Agriculture and Environment, Teramo, Italy

^2^  University of L’Aquila, Department of Life, Health and Environmental Sciences, L’Aquila, Italy

Brain-derived neurotrophic factor (BDNF) is a key molecule in neuron survival, growth, and differentiation during the development of the central nervous system (CNS), and recent studies have also documented its ability to modify CNS structure and function in adulthood. It is thus not surprising that BDNF has been linked to several neurological diseases, including epilepsy, and it has been reported that seizure activity increases BDNF expression and protein and that modulation of BDNF signal transduction inhibits the development of the epileptic state. However, the exact role of BDNF in the etiology of epilepsy disease still needs to be clarified. In order to acquire such information and comply with 3Rs principles on animal experimentation, here, we report new data using zebrafish at early-life stages, before independent feeding, as a valuable animal model to monitor seizure-like behaviors as well as molecular mechanisms associated to altered phenotypic outcomes. Zebrafish larvae have been exposed to pentylenetetrazole (PTZ), developing changes in locomotor behavior within a few minutes after the treatment (approximatively after 5–10 min), thus complying with an epileptic like-behavior. Moreover, zebrafish larvae exposed to PTZ had an increase of approximately five-fold in bdnf gene expression compared to the negative controls, and preliminary data also showed a consistent decreased trend in DNA methylation at gene promoter. Our results confirm the effectiveness of the PTZ zebrafish model of epilepsy and provide new evidence on the role of bndf gene regulation in epileptogenesis. 

## 27. Effects of MAGL Inhibitor on Striatal Neuroinflammation and Synaptic Dysfunction in Experimental Multiple Sclerosis

Livia Guadalupi ^1^, Valentina Vanni ^2^, Francesca Romana Rizzo ^1^, Alessandra Musella ^3^, Krizia Sanna ^1^, Diego Fresegna ^2^, Antonietta Gentile ^2^, Francesca De Vito ^4^, Sara Balletta ^1^, Silvia Caioli ^4^, Ludovic Collin ^5^, Anto Pavlovic ^5^, Sven Schippling ^5^, Diego Centonze ^4^ and Georgia Mandolesi ^3^

^1^  University of Tor Vergata, Department of Systems Medicine, Roma, Italy 

^2^  IRCCS San Raffaele Roma, Synaptic Immunopathology Lab, Rome, Italy 

^3^  University of Rome San Raffaele, Department of Human Sciences and Quality of Life Promotion, Rome, Italy 

^4^  IRCCS Istituto Neurologico Mediterraneo (INM) Neuromed, IRCCS Istituto Neurologico Mediterraneo (INM) Neuromed, Pozzilli, Italy 

^5^  F. Hoffmann-La Roche Ltd. Roche, Innovation Center Basel, Basel, Switzerland

Multiple sclerosis (MS) is an inflammatory neurodegenerative disorder in which the neuronal compartment is affected since the early stages of the disease. Data from MS patients and the MS mouse model, experimental autoimmune encephalomyelitis (EAE), have underscored a harmful but potentially reversible inflammatory synaptopathy in several brain area and a significant alteration of the endocannabinoid system (ECS). Studies from the EAE model have shed a light on the biological effects of endocannabinoids (eCBs) -anandamide (AEA) and 2-arachidonoylglycerol (2AG)- and their receptors (CB1R, CB2R and TRPV). Of note, recent evidence showed that the inhibition of monoacylglycerol lipase (MAGL), the key hydrolytic enzyme responsible for 2-AG inactivation, can exert a beneficial effect on EAE disease, but the mechanism is still unclear. Here, we took advantage of a reversible MAGL inhibitor (MAGLi) to investigate for the first time its effects on motor disability, neuroinflammation and synaptopathy in EAE mice. Our data clearly indicate beneficial effects of MAGLi treatment in both ex vivo and in vivo conditions in EAE mice. We observed that MAGLi treatment is able reduce the clinical disability of the mice. Electrophysiological recordings revealed a recovery of the spontaneous glutamatergic current frequency in the striatum of EAE mice in association with an effective enzymatic MAGL inhibition and increased 2AG levels. Moreover, we observed by immunofluorescence analysis a significant reduction of striatal microgliosis. In particular, we characterized the effects of MAGLi on microglia activation phenotype by a detailed morphological study, using the Sholl and Skeleton analysis.

Overall, we demonstrated that an up-regulation of the endocannabinoid tone induced by MAGL inhibition is potentially involved in the recovery of both inflammatory status and glutamatergic alterations mediated by CB1 receptor occupancy in EAE mice.

## 28. Approaching Behavior and Its Synaptic and Transcriptomic Signatures in Medial Prefrontal Cortex Pyramidal Neurons: The Involvement of Excitatory Neurotransmission and Immune System

Anna Panuccio ^1^, Juliette Gimenez ^2^, Andrea Termine ^3^, Carlo Fabrizio ^3^, Giuseppe Sciamanna ^4^, Francesca Balsamo ^5^, Noemi Passarello ^6^, Marta Tiberi ^7^, Alessandro Matteocci ^7^, Silvia Caioli ^8^, Luana Saba ^9^, Marco De Bardi ^10^, Francesco Della Valle ^11^, Valerio Orlando ^11^, Valerio Chiurchiù ^12^ and Daniela Laricchiuta ^13^

^1^  IRCCS Santa Lucia Foundation, Laboratory of Experimental and Behavioral Neurophysiology; University Sapienza of Rome, PhD program in Behavioral Neuroscience, Department of Psychology, Rome, Italy 

^2^  IRCCS Santa Lucia Foundation, Molecular Neurobiology Unit, Experimental Neurology, Rome, Italy 

^3^  IRCCS Santa Lucia Foundation, Data Science Unit, Rome, Italy 

^4^  UniCamillus-Saint Camillus International University of Health Sciences; IRCCS Santa Lucia Foundation, Laboratory of Neurophysiology and Plasticity, Rome, Italy 

^5^  IRCCS Santa Lucia Foundation, Laboratory of Experimental and Behavioral Neurophysiology; Guglielmo Marconi University, Department of Human Sciences, Rome, Italy 

^6^  Federico II University of Naples, Department of Humanities, Naples, Italy 

^7^  IRCCS Santa Lucia Foundation, Laboratory of Resolution of Neuroinflammation, Rome, Italy 

^8^  IRCCS Neuromed, Unit of Neurology, Pozzilli, Italy 

^9^  University of Campus Biomedico, Department of Sciences and Technologies for Humans and Environment, Rome, Italy 

^10^  IRCCS Santa Lucia Foundation, Neuroimmunology Unit, Rome, Italy 

^11^  King Abdullah University of Science and Technology (KAUST), Biological Environmental Science and Engineering Division, KAUST Environmental Epigenetics Program, Thuwal, Saudi Arabia

^12^  Intitute of Translational Pharmacology, CNR; IRCCS Santa Lucia Foundation, Laboratory of Resolution of Neuroinflammation, Rome, Italy 

^13^  IRCCS Santa Lucia Foundation, Laboratory of Experimental and Behavioral Neurophysiology, Rome, Italy

Approaching (AP) and avoiding (AV) tendencies are basic behavioral aptitudes in responding to rewarding and aversive stimuli, and their balancing (BA) tendency is critical for successful adaptation to the environment. The AP tendency is associated to novelty seeking and it has important evolutionary value in the identification of new sources of reward. However, the AP tendency exposes individuals to potential risks, increasing the predisposition to externalizing behaviors, such as attention deficit and hyperactivity disorder, addiction, and eating disorders. In this framework, even if the medial prefrontal cortex (mPFC) is a crucial hub that supports AP behavior by sustaining attention towards relevant and novel stimuli, its specific synaptic and transcriptomic signatures have not yet been identified. In this research, we used an experimental model of individual differences, permitting the selection of a subpopulation of mice that spontaneously responded with AP or BA behaviors toward conflicting emotional stimuli, and expressed yellow fluorescent protein (YFP) in pyramidal neurons of mPFC. Patch-clamp electrophysiological recordings showed that mPFC pyramidal neurons of AP mice had a significantly higher frequency of spontaneous excitatory post-synaptic currents when compared with BA mice. YFP-expressing pyramidal neurons from mPFC of AP and BA mice have been sorted to purify cell-specific RNA for a transcriptome-wide analyses. The omic results showed differential gene expression between AP and BA mice in the pathways associated to the regulation of immune system. Namely, AP mice were characterized by a gene overexpression for immune system response pathways and a significant change in cell number and activation of specific peripheral and central immune cells, such as CD3+ T lymphocytes and microglia. Overall, our findings suggest that, in the mPFC, both the increased excitatory neurotransmission and the altered immune response are crucial underpinnings of AP tendency.

## 29. Antioxidant and Anti-Inflammatory Role of Grapefruit IntegroPectin

Chiara Valenza ^1^, Miriana Scordino ^1^, Giulia Urone ^1^, Antonino Scurria ^2^, Mario Pagliaro ^2^, Rosaria Ciriminna ^2^, Francesco Meneguzzo ^3^, Giuseppa Mudò ^1^, Angela Bonura ^4^ and Valentina Di Liberto ^1^

^1^  Università degli Studi di Palermo, Biomedicina, Neuroscienze e Diagnostica Avanzata, Palermo, Italy 

^2^  Consiglio Nazionale delle Ricerche, Istituto per lo Studio dei Materiali Nanostrutturati, Palermo, Italy 

^3^  Consiglio Nazionale delle Ricerche, Istituto per la Bioeconomia, Sesto Fiorentino (FI), Sesto Fiorentino, Italy 

^4^  Consiglio Nazionale delle Ricerche, Istituto di Farmacologia Traslazionale, Palermo, Italy

Oxidative stress, one of the major mechanisms involved in neurodegenerative diseases, alters numerous cellular processes, such as mitochondrial functionality, DNA repair, and cell signaling, with the propagation of cellular injury leading to neurodegeneration. Recently, a new pectin, called IntegroPectin, particularly rich in citrus absorbed flavonoids and terpenes, was extracted from citrus processing waste via hydrodynamic cavitation in water only. This sustainable extraction method is exceptional in highly preserving the rhamnogalacturonan RG-I region, which is essential in the structure and function of the pectin. Tested on neuronal and microglial cells, grapefruit IntegroPectin proved to be effective in protecting different cells from apoptosis after exposure to oxidizing agents, reducing the amount of intracellular reactive oxygen species (ROS) and activating intracellular signaling cascades involved in cell protection. Preliminary results also suggest that IntegroPectin may modulate inflammatory phenomena. These data, alongside the absence of toxicity of this new pectic biomolecule, suggest a potential therapeutic role of grapefruit IntegroPectin. Though preliminary, these results support experimentation on preclinical models of neurodegenerative diseases, widely known as complex pathologies marked by extensive phenomena of oxidative stress and inflammation.

## 30. Possible Role of the Sympathetic Nervous System in the Definition of Glioblastoma Immune Microenvironment

Erika Ricci ^1^, Maria Cristina Mariani ^2^, Eleonora Cornacchia ^1^, Paolo Malatesta ^3,4^, Irene Appolloni ^4^, Davide Ceresa ^3^, Nicole Kerlero de Rosbo ^1^, Antonio Uccelli ^1,2^ and Tiziana Vigo ^1^

^1^  Laboratory of Neurosciences, IRCCS Polyclinic Hospital San Martino, Genoa, Italy 

^2^  Department of Neurosciences, Ophthalmology, Genetics, Maternal and Child Health, University of Genoa, Genoa, Italy 

^3^  Cellular Oncology Unit, IRCCS Polyclinic Hospital San Martino, Genoa, Italy 

^4^  Experimental Biology Unit, Department of Experimental Medicine (DIMES), University of Genoa, Genoa, Italy

Glioblastoma (GBM) is a brain tumor associated with neuroinflammation. Both low-grade and high-grade tumors are surrounded by activated microglia and contain infiltrating immune cells. However, the immune microenvironment changes during progression from low-grade to high-grade GBM tumors. In fact, T lymphocytes predominate in low-grade tumors, while immunosuppressive macrophages are the main immune population infiltrating high-grade tumors. Brain inflammation alters the function of the sympathetic nervous system (SNS), which regulates the generation of immune cells in the bone marrow (BM) and thymus. It is hypothesized that intrinsic changes in tumor gene expression, together with changes in immune cell generation due to altered SNS transmission in the BM and thymus, may contribute to shaping the immune microenvironment during tumor progression. To define the tumor-intrinsic changes associated with its progression, we performed single-cell RNA sequencing (sc-RNAseq) analysis of murine GBM cells isolated from low- or high-grade tumors. Among the 11 clusters of GBM cells identified, those characterized by high HLA expression were overrepresented in high-grade tumors, suggesting that the reduced frequency of tumor-infiltrating lymphocytes in these tumors was due to mechanisms extrinsic to the cells. We then evaluated the generation of immune cells in the BM and thymus of mice with low- or high-grade tumors by multiparametric analysis with flow cytometry. We observed an increased generation of B lymphocytes and T lymphocytes in mice with low-grade tumors, but not in those with high-grade tumors, in which the SNS neurotransmitter norepinephrine (NE) and the frequency of hematopoietic stem cells in the BM were altered. Our data suggest that mechanisms extrinsic to the tumor, possibly mediated by the SNS, alter lymphocyte generation in the BM and thymus and may help define a different immune microenvironment in low- and high-grade GBM.

## 31. Understanding the Role of Microglial Extracellular Vesicles in Neuroinflammation Spreading: An In Vitro Study

Francesca De Chirico ^1^, Francesca Massenzio ^1^, Sabrina Petralla ^2^, Eleonora Poeta ^1^, Giorgia Babini ^1^, Giampaolo Zuccheri ^1^ and Barbara Monti ^1^

^1^  University of Bologna, Dept. of Pharmacy and Biotechnology, Bologna, Italy 

^2^  University of Heidelberg, Institute of Pharmacy and Molecular Biotechnology, Heidelberg, Germany

Neuroinflammation is a crucial mechanism that commonly underlies the majority of the neurodegenerative diseases including Alzheimer’s, Parkinson’s, Huntington’s disease, and amyotrophic lateral sclerosis. Microglia, the immune cells of the brain, play a critical role in the inflammatory condition following the onset of neuropathology. In fact, it was shown that microglia display the M2 anti-inflammatory phenotype at the early stages of the disease, switching to the M1 classically activated subtype as the disease progresses. The shift of microglial phenotype from M2 to M1 phenotype could be related to a change in the protein and/or microRNAs content in extracellular vesicles (EVs) involved in intercellular communication. This suggests that the spreading of neuroinflammation could be mediated by the release of vesicles in the extracellular environment and, therefore, by the effect that the content of these vesicles has on surveying microglia and other cell types.

To evaluate whether activation could be transmitted among microglial cells, activation was pharmacologically induced in a microglial murine cell line (N9) by using LPS towards a M1 phenotype or ATP towards M2. Then, non-activated microglia were treated with the media conditioned by differentially activated microglia or the isolated vesicles.

Furthermore, we investigated the expression profiles of microRNAs, identified as regulators of microglial activation, particularly miRNA-155, miRNA-124, miRNA-34a, and miRNA-125b, which are known to be dysregulated in different pathological states. We focused on miRNA-34a contribution in neuroinflammation spreading and we tried to downregulate its expression using cleaving sequences of anti-mir34a DNAzyme delivered by DNA nanostructures.

Given that evidence, the role of EVs miRNAs released by microglia deserves to be deeply investigated both as potential therapeutic targets and as biomarkers for neurodegenerative diseases.

## 32. Systemic Inflammation Upregulates the Expression of Oxysterol 7α,25OHC-Synthesising Enzymes in the Blood-Brain Barrier

Fionä Caratis and Aleksandra Rutkowska

Medical University of Gdansk, Department of Anatomy and Neurobiology, Gdansk, Poland

The Epstein-Barr virus-induced gene 2 (EBI2), alongside its most potent ligand the oxysterol 7α,25OHC, is involved in several neuroinflammatory and neurodegenerative disorders and plays a key role in modulating innate immunity. Oxysterol 7α,25OHC is synthesized from cholesterol with the enzymes CH25H and CYP7B1 and degraded with HSD3B7. EBI2 activated by 7α,25OHC coordinates immune cell positioning in the secondary lymphoid tissues, enabling proper humoral and cellular immune responses. This coordinated lymphocyte positioning is possible with a tightly regulated concentration gradient of 7α,25OHC in the secondary lymphoid tissue formed by CH25H, CYP7B1 and HSD3B7 expressing cells. Lipopolysaccharide (LPS) modulates the expression of EBI2, 7α,25OHC, CH25H, CYP7B1 and HSD3B7 enzymes in vitro and in vivo. Notably, the concentration of 7α,25OHC and CH25H increases in the brain in the early phases of the experimental autoimmune encephalomyelitis, a murine model of multiple sclerosis, leading to an enhancement of EBI2-expressing lymphocytes infiltration in the central nervous system. Here, we induced systemic inflammation in mice with a single peripheral high-dose injection of LPS and analyzed the expression of pro-inflammatory cytokines, blood–brain barrier (BBB)-forming proteins, as well as the expression of EBI2 and 7α,25OHC-related enzymes in the brain. LPS injection increased the expression of pro-inflammatory cytokines, IL-6 and IL-1β, indicating neuroinflammation after peripheral immune challenge. Tight junction protein, occludin, adhesion protein, N-cadherin, as well as EBI2 were downregulated in the brain, while the 7α,25OHC-related enzymes were upregulated in the BBB after LPS challenge. Taken together, the data indicate the blood–brain barrier as a source of oxysterol-synthesizing enzymes after systemic inflammation, further implicating the EBI2/oxysterol signaling in neuroinflammatory diseases.

## 33. Specialized Pro-Resolving Mediator RvD1 Reduces Neuroinflammation in a Transgenic Rat Model of Parkinson’s Disease

Marta Tiberi ^1^, Alessandro Matteocci ^1^, Mariangela Massaro ^2^, Federico Fazio ^1^, Stefano Sacchetti ^4,5^, Davide Decandia ^4,5^, Vladimiro Batocchi ^1^, Debora Cutuli ^4,5^, Nicola Biago Mercuri ^2,3^ and Valerio Chiurchiù ^1,6^

^1^  Laboratory of Resolution of Neuroinflammation, IRCCS Santa Lucia Foundation, Rome, Italy

^2^  Department of Experimental Neuroscience, IRCCS Santa Lucia Foundation, Rome, Italy

^3^  Neurology Unit, Tor Vergata Hospital, Rome, Italy

^4^  Laboratory of Experimental Neurophysiology and Behaviour, IRCCS Santa Lucia Foundation, Rome, Italy

^5^  Department of Psychology, Sapienza University of Rome, Italy

^6^  Institute of Translational Pharmacology, National Research Council, Rome, Italy 

The neuroinflammatory processes in Parkinson’s disease (PD) are usually associated with activation of the immune system caused by a growing aggregation of α-synuclein (α-Syn) in central nervous system. The active immune response in the brain of PD patients leads to the infiltration of lymphocytes, production of cytokines, and microgliosis, and these features could be a consequence of failure to resolve inflammation, a process mediated by a superfamily of endogenous lipids termed specialized pro-resolvin mediators (SPMs). A previous study from our group has shown that precocious treatment with resolvin D1 (RvD1) prevents the onset of PD by attenuating immune response in a rat model of PD. Herein, we explored the long-term effect of RvD1 in α-Syn rats by treating them with intraperitoneal injections twice a week, starting at early stage of the disease (2 months old) until the symptomatic phase (12 months old). Hence, we assessed motor deficit evaluated through Rotarod test and the infiltration of the main CD45+ leukocyte cell populations, i.e., CD3+ T-cells, CD45RA+ B-cells, CD161+ NK-cells and CD45/CD11bhigh macrophages, within substantia nigra and striatum by flow cytometry. We found that α-Syn rats showed a higher degree of nigral and striatal infiltration of all cell subsets compared to age-matched wild-type rats and that RvD1 treatment not only ameliorated motor deficits but also reduced their infiltration in both anatomical regions. Furthermore, although the percentage of CD45lowCD11b+ microglial cells remained unchanged between the different experimental groups, we observed that microglia of α-Syn rats shifted from a pro-inflammatory M1-like to a pro-resolving/anti-inflammatory M2-like immunophenotype upon RvD1 treatment, in terms of modulation of their respective M1 (CD68, CD86, MHC-II) and M2 (CD206, TREM2) markers. These results suggest that RvD1 is able to delay disease progression by blunting neuroinflammation and inducing a microglia-driven pro-resolving response.

## 34. Evaluation of D-Loop Methylation Level and mtDNA Copy Number in Aicardi-Goutières Patients

Francesca Dragoni ^1^, Jessica Garau ^2^, Maria Garofalo ^3^, Matteo Bordoni ^4^, Eveljn Scarian ^4^, Rosalinda Di Gerlando ^3^, Costanza Varesio ^5^, Simona Orcesi ^5^, Orietta Pansarasa ^4^, Stella Gagliardi ^3^

^1^  University of Pavia, Department of Biology and Biotechnology “L. Spallanzani”, Pavia, Italy 

^2^  IRCCS Mondino Foundation, Neurogenetics Research Centre, Pavia, Italy 

^3^  IRCCS Mondino Foundation, Molecular Biology and Transcriptomics, Pavia, Italy 

^4^  IRCCS Mondino Foundation, Cellular Model and Neuroepigenetics, Pavia, Italy 

^5^  IRCCS Mondino Foundation, Child Neurology and Psychiatry Unit, Pavia, Italy

Aicardi-Goutières syndrome (AGS) is a pediatric rare disorder that affects the brain, the immune system and the skin. Mutations in nine AGS genes lead to an accumulation of endogenous nucleic acids (NAs) which are recognized as foreign NAs of viral origin by the organism triggering an abnormal Interferon-alpha (IFN-α) mediated immune response. Mitochondrial dysfunction may lead to the release of mtDNA and trigger immunological pathways with the production of IFN-α. Alterations in methylation levels of the mitochondrial displacement loop (D-loop) region, which governs mtDNA replication, were recently discovered in other neurological disorders, i.e., Alzheimer’s disease and amyotrophic lateral sclerosis. Up to now, nothing has been known about methylation levels in the D-loop area in AGS patients.

The purpose of this study was to look at D-loop methylation levels and mtDNA copy number in AGS patients and healthy controls’ peripheral blood. In peripheral blood cells from 25 AGS patients and 22 age- and sex-matched controls, pyrosequencing analysis of D-loop methylation levels and quantitative measurement of mtDNA copy number were performed.

D-loop methylation levels were considerably greater in AGS patients compared to controls, with the RNASEH2B A177T mutation driving the difference. In addition, the number of copies of mtDNA was much higher in AGS patients, with the RNASEH2B mutated patients accounting for the majority of the variance. In total samples, controls, and AGS patients, there was a positive correlation between mtDNA copy number and D-Loop methylation levels. Furthermore, a strong positive correlation was found between D-Loop methylation and the age of subjects in the controls and between the number of copies of mtDNA in AGS patients and the age of samples, too.

These data imply that D-loop methylation and mitochondrial replication are closely related, and that changes of methylation pattern could be used as a compensatory strategy for mitochondrial dysfunction.

## 35. The Role of Glial Cells in the Adaptive and Maladaptive Response to Acute Stress: Evidence from a Preclinical Model

Roberta Facchinetti ^1^, Marta Valenza ^1^, Tiziana Bonifacino ^^2^^, Marco Milanese ^^2^^, Laura Musazzi ^3^, Luca Steardo ^1^ and Caterina Scuderi ^1^

^1^  Sapienza Università di Roma, Dipartimento di Fisiologia e Farmacologia “V.Erspamer”, Roma, Italy 

^2^  Università di Genova, Dipartimento di farmacia, DIFAR, Genova, Italy 

^3^  Università degli Studi di Milano-Bicocca, Dipartimento di Medicina e Chirurgia, Milano, Italy

Each individual reacts differently to stress. When the response is physiological, it promotes adaptive plasticity; when it is excessive or unregulated, it induces maladaptive harmful effects. Considering the innumerable homeostatic functions in which glial cells are implicated, their involvement in the response to chronic stress has been already established. Little evidence is instead available regarding a possible role of these cells in the response to an acute stress. To fill this gap, and to explore the presence of glial factors determining the adaptive/maladaptive trajectory, Sprague-Dawley rats were exposed to a footshock stress (FS). Rat baseline sucrose intake was monitored for 5 weeks before stress and after FS. Animals with a decrease in sucrose intake <10% after FS respect to baseline consumption were considered resilient (RES) and animals with a variation >25% compared to baseline consumption were considered vulnerable (VUL). Through PCR, immunofluorescence, and Western blot analyses, we investigated the morphofunctional alterations affecting astrocytes, microglia, and neurons 24 and 48 h after FS in the prefrontal cortex of non-stressed, RES and VUL animals. Results obtained showed that, in VUL animals, FS exposure triggers a proinflammatory response guided by glial cells reactivity, reducing the expression of neurotrophic factors and impairing neuronal integrity. RES animals showed a better ability to metabolize glutamate. This study establishes, for the first time, the involvement of glial cells in the response to acute stress. More interestingly, our data reveal that astrocytes and microglia respond differently to acute stress in VUL and RES animals, potentially designating these cells as a target for personalized medicine.

## 36. Cortical Rewiring Following Peripheral Injection of Botulinum Neurotoxin Type A

Alexia Tiberi ^1^, Verediana Massa ^1^, Marco Pirazzini ^2^, Ornella Rossetto ^2^ and Laura Restani ^1^

^1^  National Research Council (CNR), Neuroscience Institute, Pisa, Italy 

^2^  University of Padua, Department of Biomedical Sciences, Padua, Italy

Botulinum neurotoxin type A1 (BoNT/A1) is a bacterial metalloprotease that can cleave SNAP-25 (Synaptosomal-Associated Protein, 25kDa), thus inhibiting synaptic vesicle fusion and ultimately blocking neuronal activity. BoNT/A1 action is potent, specific, long-lasting, yet reversible. These features underlie its wide use in human therapy for treating neurological conditions characterized by neuronal hyperactivity. Interestingly, evidence suggests that a fraction of BoNT/A1 can undergo long-distance axonal transport, possibly mediating a direct effect on central circuits. Recently, fMRI studies on human patients with dystonia have indeed demonstrated wide and long-lasting changes in the cortical activity after BoNT/A1 injection, at timescales that are not compatible with the peripheral blockade at the level of the neuromuscular junction (NMJ).

Here, we assessed whether and how BoNT/A1 peripheral injections can influence motor cortical areas, affecting the morpho-functional physiology of pyramidal cortical neurons connected with BoNT/A1-affected central nuclei. To visualize dendritic spines, three-month-old Thy1-GFP mice, expressing GFP in layer V pyramidal neurons, were injected with BoNT/A1 (5 U/kg) in the whisker pad. Ex vivo dendritic spine analysis revealed a striking decrease in spine density in cortical motor areas 30 days after BoNT/A1 injection, while whisker paralysis lasted only around 10 days. Moreover, we observed an increase in stubby spines, known to be an immature spine type that could either be new or in the process of being eliminated. To understand the mechanism underlying spine loss, we then measured spine dynamics longitudinally in awake mice using two-photon microscopy. Imaging of apical dendrites in the motor cortex before and after BoNT/A1 injection revealed a decrease in spine density already 15 days after the peripheral insult, confirming our ex vivo data. Moreover, we observed an increase in spine elimination at day 15 after BoNT/A1 injection. Overall, our data reveal profound morphological changes in cortical neurons after intramuscular BoNT/A1 injection, which persist longer than the peripheral effect at the NMJ. Our hypothesis is that cortical spine remodeling plays a key role in the therapeutic action of BoNT/A1 in neuropathologies and strongly contributes to the long-lasting benefits observed in patients.

## 37. Assessing the Contribution of Altered Cholinergic Signaling in ASD Social Deficits

Alice Tartacca ^1,†^, Rocco Pizzarelli ^1,†^, Domenico Pimpinella ^1^, Hannah Monyer ^1,2^, Marilena Griguoli ^1,3^

^1^  European Brain Research Institute (EBRI), Fondazione Rita Levi-Montalcini, Rome, Italy

^2^  Department of Clinical Neurobiology of the Medical Faculty of Heidelberg University and German Cancer Research Center (DKFZ), Heidelberg, Germany

^3^ National Research Council-Institute of Molecular Biology and Pathology (CNR-IBPM), Rome, Italy.

† Equally contributing authors.

Cholinergic (ChAT) neurons in the brain control several cognitive functions such as attention, learning and memory. These neurons are mainly localized in subcortical regions including the medial septum/diagonal band of Broca complex (MSDB). Our laboratory has previously shown that inhibition of MSDB ChAT neurons affects social memory, i.e., the ability to discriminate between novel and familiar subjects. Alterations in ChAT neurons have been observed in autism spectrum disorders (ASD), neurodevelopmental conditions characterized by social isolation, stereotyped movements and communication challenges. Some forms of ASD are associated with mutations in genes encoding for synaptic proteins including the neuroligin 3 (NLG3). NLG3 is a postsynaptic adhesion molecule that binds its presynaptic partner neurexin and stabilizes both excitatory and inhibitory synapses. Mice lacking NLG3 (NLG3 knockout, NLG3KO) show deficits in social interaction and social memory similar to those observed in autistic patients, thus NLG3KO mice are considered a valid ASD animal model. Here, we propose to study whether cholinergic dysfunction accounts for social memory deficits observed in NLG3KO mice. Preliminary data showed that NLG3KO mice had a reduced number of MSDB ChAT neurons as compared to control littermates. Furthermore, patch clamp recordings from ChAT neurons revealed an altered synaptic transmission. Ultimately, conditional suppression of Nlg3 expression in MSDB ChAT neurons, using a microRNA-based viral strategy (mi-RNA-Nlg3), induced an impairment in social memory, similarly to what observed in NLG3KO mice. Patch clamp recordings from MSDB ChAT neurons will clarify whether social memory deficits are associated to synaptic dysfunction in mice carrying mi-RNA-Nlg3. Moreover, the rescue of Nlg3 expression in MSDB ChAT neurons of NLG3KO mice will corroborate the evidence that cholinergic dysfunction may cause social memory deficits in ASD.

## 38. Rearrangements of Peritumoral Tissue That Take Place During Glioma Progression

Elisa De Santis ^1^, Cristina Spalletti ^1^, Marta Scalera ^1^, Sabrein Haddad ^2^, Daniele Cangi ^1^, Marco Mainardi ^1^, Silvia Landi ^1^, Matteo Caleo ^3^ and Eleonora Vannini ^1^

^1^  Consiglio Nazionale delle Ricerche, Istituto Neuroscienze, Pisa, Italy

^2^  Medical University Innsbruck, Dep. of Physiology and Medical Physics, Innsbruck, Austria

^3^  Università di Padova, Dipartimento di scienze biomediche, Padova, Italy

Glioblastoma (GB) is the most malignant and aggressive form of brain tumor. Despite a strong effort in finding new and effective therapeutic strategies, GB remains associated with high morbidity and mortality and the median survival is 12–15 months after diagnosis. For a long time, cancer research has mainly focused on understanding the biology of glioma cells and investigating the aberrant pathways that guide tumor onset and progression; however, it has been recently found that the interaction between GB cells and the tumor microenvironment (TME) is crucial in driving tumor growth. Specifically, recent findings highlighted the importance of clarifying the role of peritumoral tissue in GB progression and the need of a more detailed picture on the interactions between tumoral and neural tissue. In this context, our project aims to investigate the plastic rearrangement of cortical areas that takes place along with GBM progression. Using Thy1-ChR2 glioma-bearing mice and optogenetics, we checked the responsivity of motor cortex at three different time points (i.e., baseline, before glioma cells injection, and 14 and 21 days after tumor implantation). We found that glioma-bearing mice showed a strong remapping of cortical motor areas and an increased threshold required for eliciting a forelimb movement. Immunohistochemical analyses revealed a downregulation of PNNs and of specific inhibitory markers in the peritumoral cortex. These findings demonstrate that the peritumoral tissue undergo through a strong biochemical reorganization along with glioma progression. Increasing our knowledge about these changes will help us to understand the mechanisms underlying GBM progression, which might be useful to develop new and finally effective therapeutic approaches to counteract this terrible disease.

## 39. An Innovative Technique to Anticipate the Diagnosis of Glioblastoma: Analysis of Extracellular Vesicles in Liquid Biopsies

Mariassunta De Luca, Cristina Limatola and Myriam Catalano

Sapienza University of Rome, Department of Physiology and Pharmacology, Rome, Italy

Glioblastoma (GBM) is the most common and malignant primary brain tumor in humans, it has a high level of invasiveness and chemoresistance. Its diagnosis requires neurological, radiological, and histological examinations when the tumor has already reached a critical mass. Current pharmacological treatment is based on temozolomide (TMZ), an alkylating agent, associated with surgical removal. In the last decades, the analysis of liquid biopsies (plasma, urine, CSF) obtained a relevant role in the diagnosis of different diseases, including tumors. They represent a non-invasive technique, permit to collect serial samples, and monitor dynamic changes in patients.

All the cells of the body, including brain cells, communicate each other releasing cytokines, growth factors, as well as extracellular vesicles (EVs), both in physiological and pathological processes. Recently, studies suggested that GBM cells release more EVs than healthy cells and EVs increase with the progression of GBM. EVs are composed of bilayer membranes and contains specific lipids, proteins and nucleic acids, such as mRNA, miRNA, and ctDNA, that can modulate the functions of recipient cell. EVs can be distinguished on biogenesis, content, and size. Specifically, medium/large (m/lEVs) and small (sEVs) indicate, respectively, EVs with a diameter above or below 200 nm.

In this study, we used an in vivo glioma model on adult male C57BL6/N mice and we inoculated murine glioma cells (GL261 cell line) in the right striatum. We collected brain tissue and plasma from glioma-bearing mice and control animals at different time points. We separated EVs (both sEVs and m/lEVs) using differential centrifugation. We analyzed the expression of some miRNAs (miR21, miR124, miR222) in both sEVs and m/lEVs in all samples.

The miRNAs in the EVs isolated from the brain could be correlated with the EVs isolated from plasma and we could use EVs and their contents as potential prognostic markers in GBM patients to anticipate the diagnosis.

## 40. Investigate Age-Dependent Myelin Alterations in Structural Network Properties

Sara Bosticardo ^1^, Simona Schiavi ^2^, Sabine Schaedelin ^3^, Muhamed Barakovic ^4^, Po-Jui Lu ^4^, Matthias Weigel ^4^, Daducci Alessandro ^1^ and Cristina Granziera ^4^

^1^  University of Verona, Diffusion Imaging and Connectivity Estimation (DICE) Lab, Department of Computer Science, Verona, Italy

^2^  University of Genoa, Department of Neuroscience, Rehabilitation, Ophthalmology, Genetics, Maternal and Child Health (DINOGMI), Genova, Italy

^3^  University Hospital Basel, Clinical Trial Unit, Department of Clinical Research, Basel, Switzerland

^4^  University Hospital Basel, Translational Imaging in Neurology (ThINK) Basel, Department of Biomedical Engineering, Basel, Switzerland

Normal brain aging is characterized by different structural alterations: myelin sheaths become less compact, the number of axons decreases, and axons become less myelinated. There is also evidence that demyelination contributes to the loss of brain plasticity that occurs during aging. Here, we combined diffusion MRI with myelin volume fraction (MVF) mapping to evaluate the sensitivity of MVF as a marker for age-dependent myelin alterations. We computed whole brain probabilistic tractographies of 85 healthy subjects (46f, 18–69y) and built the connectomes with a grey matter parcellation of 85 regions of interest. To compute myelin-weighted connectomes we used the state-of-the-art tractometry method and new framework called myelin streamline decomposition (MySD), which allow to recover the actual myelin value for each reconstructed fiber. We calculated 3 network metrics: Global Efficiency (information exchange), Modularity (network segregation), and Mean strength (nodal strength). We tested the possible relation between age, age2, and network alterations using sex and white matter volume as confounder factors. We employed the same model to predict these changes using leave-one-out cross-validation. Global efficiency (MySD: Age2 p = 0.03, R2 = 0.61; Tractometry: Age2 p = 0.04, R2 = 0.26) and mean strength (MySD: Age2 p = 0.03, R2 = 0.61; Tractometry: Age2 p = 0.04, R2 = 0.26) appeared to be related with aging: myelination increases up to 35/40 years, then starts to decrease. Although both methods were sensitive to these changes, data calculated using MySD showed higher adjusted R2. Furthermore, results concerning age prediction showed a considerably lower error for MySD (Global Efficiency: 0.44 MySD, 0.89 Tractometry; Mean Strength: 0.25 MySD, 0.77 Tractometry).

In conclusion, MVF weighted connectomes computed using MySD, appeared to be more sensitive as well as more specific in capturing age-dependent myelin alterations compared to those computed using tractometry only.

## 41. Neuroprotective Effects of Montelukast Treatment in a Rat Model of Huntington-like Neurotoxicity: A PET Covariance Study

Margherita Tassan Mazzocco ^1^, Valentina Murtaj ^1^, Daniel Martins ^2^, Federico Turkheimer ^2^, Sara Belloli ^3^, Rosa Maria Mores ^1^

^1^  Medicine and Surgery Department, University of Milano-Bicocca, Milano, Italy 

^2^  Department of Neuroimaging, Institute of Psychiatry, Psychology and Neuroscience, King’s College London, London, UK 

^3^  Institute of Molecular Bioimaging and Physiology (IBFM), CNR, Milano, Italy

A single intra-striatal administration of quinolinic acid (QA) in rats is sufficient to induce a lesion whose features resemble Huntington’s disease. Our aim was to evaluate the effects of the leukotriene receptor antagonist Montelukast (MLK), which previously exhibited neuroprotection in preclinical models of neurodegeneration, on QA-induced neurotoxicity and brain functional connectivity using neuroimaging techniques.

A group of 16 rats underwent positron emission tomography (PET) with 2-Deoxy-2-[18F]-fluoroglucose (FDG) to assess baseline glucose metabolism. Right and left striata of all animals were then injected with QA and vehicle (VEH), respectively. Starting from the day before QA administration, rats were treated with MLK or VEH for 14 days. At 4 months after lesion, FDG PET was repeated. Pairwise correlation coefficients between regional FDG uptake values were calculated to assess brain connectivity. A graph-based analysis was applied to explore the effects of QA and MLK on network measures of connectivity, including node degree and betweenness centrality.

In VEH-treated rats, QA significantly reduced FDG uptake in the lesioned hemisphere, compared to baseline. In animals treated with MLK, FDG was maintained at pre-lesion levels. In particular, QA effect was significantly reduced in ipsi-lesional cortical regions and in the lesioned striatum. Connectivity data showed that in contra-lesional regions, such as prefrontal cortex, orbitofrontal cortex, and midbrain, MLK treatment preserved the changes in connectivity that are present in VEH-treated rats after QA, but was not able to maintain inter-hemisphere associations in the striatum. Finally, MLK counteracted the reduction in node degree and betweenness centrality observed in the lesioned hippocampus of vehicles.

In conclusion, MLK exhibited a neuroprotective effect by preserving regional brain function and connectivity from QA insult, in cortical and subcortical regions, but did not fully prevent striatal damage.

## 42. GABA Signaling and Metabolism (dys)Regulation in Spinal Muscular Atrophy

Giovanna Menduti ^1^, Giada Beltrando ^2^, Alessandro Vercelli ^2^ and Marina Boido ^2^

^1^  Neuroscience Institute Cavalieri Ottolenghi (NICO), Department of Neuroscience “Rita Levi Montalcini”, University of Turin, Regione Gonzole 10, 10043 Orbassano (TO) Turin, Italy 

^2^  Neuroscience Institute Cavalieri Ottolenghi (NICO), Department of Neuroscience, Regione Gonzole 10, 10043 Orbassano (TO) Turin, Italy

Spinal muscular atrophy (SMA) is a neurodegenerative disease, due to the lack of survival motor neuron (SMN) protein: SMA is characterized by MN degeneration (MND) and muscle atrophy. Moreover, selective degeneration of motor cortex (CRTX) pyramidal neurons was shown in SMA mice compared to controls. Even if SMA genetic causes are well known, many aspects of its pathogenesis remain unclear and the available therapeutic options show many limits, disregarding SMN-independent targets. Intriguingly, neuroprotective effects of GABA-targeting drugs (used for neurological/psychiatric disorders) were reported in SMA, although the mechanisms involved are still elusive. Therefore, we investigated the perturbations in the GABA metabolism and GABAergic-interneuron (IN) functionality in SMAΔ7 mice (a severe SMA murine model) CRTX in the late disease stage (postnatal day 12). By immunofluorescence, we observed a significant reduction of GABAergic signal (–52%) and lower density of GABA+-cells (–20%) in SMAΔ7 motor/somatosensory CRTX compared to WT controls, along with an impaired distribution and significant reduction of GAD67 (enzyme responsible of GABA synthesis) signal –42%) and GAD67+-cells (–20%). Moreover, parvalbumin Ins (the predominant GABAergic IN subtype) were found to be significantly reduced in number –30%), signal –32%), area, and branching in SMAΔ7 cortical areas. Immunoblotting and immunocytochemical analysis in SMAΔ7 further confirmed, respectively, a significant reduction in GAD67 protein levels in the sensorimotor CRTX (−43%) and lower GABA signaling in primary cortical neuron cultures. Overall, the results show a dysregulation of GABAergic synthesis and signaling in SMA mice CRTX, suggesting impaired inhibitory neurotransmission that may contribute to the SMA onset of MND, as a shared key role in other neural diseases. Further studies aimed at fully understanding and pharmacologically rescuing GABA signaling dysregulation will pave the way for new SMA treatments.

## 43. Towards Unveiling the Nexus between Axonal Granules and Polysomes in Neurological Disorders

Fabio Lauria ^1^, Federica Maniscalco ^1^, Marta Marchioretto ^1^, Emma Busarello ^1^, Federica Cella ^2^, Toma Tebaldi ^3^, Alessandra Pisciottani ^4^, Laura Croci ^4^, Cecilia Perrucci ^1^, Lorenzo Lunelli ^5^, Maria D’Antoni ^1^, Daniele Peroni ^3^, Manuela Basso ^3^, Alessandro Quattrone ^3^, Velia Siciliano ^2^, Gian Giacomo Consalez ^4^ and Gabriella Viero ^1^

^1^  Institute of Biophysics, National Research Council, Trento, Italy 

^2^  Synthetic and Systems Biology for Biomedicine, Istituto Italiano di Tecnologia, Napoli, Italy 

^3^  Centre for Integrative Biology, University of Trento, Trento, Italy 

^4^  Unit of Developmental Neurogenetics, Vita-Salute San Raffaele University, Milano, Italy 

^5^  Laboratory of Sequence and Structure Analysis for Health, Fondazione Bruno Kessler, Trento, Italy

Amyotrophic lateral sclerosis (ALS) and front temporal dementia (FTD) are neurodegenerative disorders characterized by progressive loss of motor neurons and cognitive impairment. RNAbinding proteins have been identified as major contributors to the development of neurological diseases and are known to modulate RNA synthesis, localization, and translation. However, the cellular mechanisms linking RNA dysregulation to neuropathogeneses remain largely unknown. ALS has been associated to mutations affecting the DNA/RNA-binding protein TDP43. We explored the hypothesis that TDP43 overexpression or mutation causes an imbalance between axonal granule- and polysome-associated RNAs. We developed a tag-free polysomal profiling to identify mRNAs associated to subcellular regions (cell body or axon) and sub-compartments (RNA granules or polysomes) of mouse cortical neurons. Through high-throughput sequencing and dedicated computational pipelines, we investigated translational changes induced by the overexpression of TDP43-WT or TDP43-A315T mutant and revealed a loss of balance between free and polysome-engaged RNA in the axon. These results, supported by additional data from axonal puromycilation assay and multiple in vivo validation assays, suggest that the imbalance between granule- and polysome-associated mRNAs is caused by robust degradation of specific RNAs. The massive depletion of TDP43 target RNAs leads to a translational burden on non TDP43 targets due to the increased availability of ribosomes and the hyper-translation of the remaining mRNAs. We investigated this hypothesis in a cell line model of ALS by designing ad hoc constructs, revealing the presence of a translational burden effect triggered by reduced levels of TDP43 mRNA targets. Our results point to an as yet unexplored translation-centered mechanisms linking TDP43 and ALS pathogenesis, paving the way toward a better understanding of axonal protein synthesis possibly underlying many neurodegenerative diseases.

## 44. Epigenetic and Transcriptional Dysregulation in NSCs and OPCs Proliferation Defects of AGC1 Deficiency

Eleonora Poeta ^1^, Giorgia Babini ^1^, Francesca De Chirico ^1^, Francesca Massenzio ^1^, Francesco Massimo Lasorsa ^2^ and Barbara Monti ^1^

^1^  University of Bologna, Dept. Pharmacy and BioTechnology, Bologna, Italy 

^2^  University of Bari, Department of Biosciences, Biotechnologies and Biopharmaceutics, Bari, Italy

AGC1 deficiency is an ultra-rare demyelinating disease caused by mutations in the SLC25A12 gene encoding for the mitochondrial aspartate-glutamate carrier isoform 1 (AGC1). The main common pathological features are secondary hypomyelination and altered myelin formation in CNS, most likely due to a reduction of N-acetyl aspartate (NAA) synthesis, but brain cells proliferation deficit is also present. Here, we studied whether transcriptional and epigenetic changes correlate with dysregulation of NSCs and OPCs biological mechanisms, and whether this could be counteracted by aminoacid and vitamin supplementations. We observed an altered expression of transcription factors involved in brain precursors proliferation/differentiation, as well as lower global histone acetylation and different histone acetyl transferases (HATs) and histone deacetylates (HDACs) isoforms expression and activities, in both our in vitro AGC1 deficiency models of siAGC1 Oli-neu cells (where a reduction up to 40% of carrier activity was induced by a shRNA) and SVZ-derived AGC1+/− mice neurospheres (vs. controls, respectively). Additionally, treatments with specific HATs and HDACs activity inhibitors were performed to clarify the molecular roles of these enzymes.

These data let us to suggest histone acetylation defects in brain precursor cells, and consequent transcriptional dysregulation, as a pathogenic mechanism of AGC1-deficiency proliferation/differentiation dysfunction. Therefore, given NAA represents a source of acetate for histone acetylation, experiments on amino acids and vitamins supplementations directly involved in NAA synthesis are on-going, to compensate the metabolic impairment and the lack of acetyl-CoA with the aim of restoring proliferation/differentiation dysfunction in our in vitro AGC1 deficiency models.

## 45. Molecular and Metabolic Pathways Underlying the In Vivo Anti-Amyloidogenic Action of 12A12, a Cleavage-Specific Tau Antibody Targeting the 20–22 kDa Toxic Peptide

Valentina Latina ^1^, Anna Atlante ^2^, Alessia Loviglio ^3^, Federico La Regina ^1^, Pietro Calissano ^1^ and Giuseppina Amadoro ^4^

^1^  European Brain Research Institute (EBRI), Neurodegeneration, Rome, Italy 

^2^  National Research Council (CNR), Institute of Biomembranes, Bioenergetic and Molecular Biotechnologies (IBIOM), Bari, Italy 

^3^  Università degli Studi di Roma La Sapienza, Neuroscienze Cognitive e Riabilitazione Psicologica, Rome, Italy 

^4^  National Research Council (CNR), Institute of Translational Pharmacology (IFT), Rome, Italy

The development of antibodies that selectively remove the neurotoxic target peptides (Ab and tau) from the brain, without interfering with the normal physiology of the neuronal proteins they are derived from, is crucial to ensure an effective and avoid of harmful potential side-effects treatment for Alzheimer’s disease (AD). The cleavage-specific, monoclonal Antibody (mAb) 12A12mAb is unique in its ability to selectively bind the neo-epitope generated by cleavage of caspase-3 at D25-(D25(QGGYTMHQDQ) site located at the N-terminal end of the human tau without cross-reaction with the full-length protein. When intravenously injected into 6-month-old Tg2576 mice expressing the human amyloid precursor protein (APP) with the Swedish mutation KM670/671NL, 12A12mAb selectively neutralizes its target, both into hippocampus and retina, leading to a significant improvement of behavioral, electrophysiological, neuropathological, and metabolic cerebral-retinal parameters associated with animal’s AD phenotype. In the search of the molecular and metabolic mechanisms underlying the neuroprotective action exerted in Tg2576 by 12A12mAb, we found out that 12A12mAb immunization reduces, both in the hippocampus and retina, the steady state expression levels of APP and Beta-secretase-1 (BACE-1) along the amyloidogenic route by altering the protein amount of neuron-specific BIN1 and RIN3, two key regulators of endocytic pathways. Besides, retinal and hippocampal alterations of energetic glucose metabolism that are strictly linked with Ab production (lactate production) are mitigated in Tg2576 AD mice by 12A12mAb treatment in concomitance with its local anti-amyloidogenic action. Taken together these findings indicate that the in vivo beneficial action of 12A12mAb involves, both in hippocampus and in retina, the energy-dependent modulation of the dynamic convergence of APP and BACE-1 into Rab5-positive early endosomes, leading to Aβ generation.

## 46. Modeling of Nigro-Striatal Circuits through the Generation of Human 3D Organoids

Manuela Magni

IRCCS Ca Granda Policlinico di Milano, Neurologia, Milano, Italy

Recent advances in stem cell culture techniques led to the development of protocols for in vitro 3D cultures, called organoids, capable of developing organo-typically and exerting organ-specific functions. These complex structures are particularly useful to mimic the embryonic organogenesis. Furthermore, it has been found that by combining two distinct brain regions it is possible to achieve higher-order thought processes. These assembloids are 3D structures formed from the fusion and functional integration of multiple cell types. And, most important, it has been seen that they mimic the complex cellular interactions from which organs arise in the body.

Here, the aim of the project is to generate an assembloid by fusing two distinct brain regions. Specifically, it involves fusing a mesencephalic organoid with a striatal one in order to study the nigro-striatal circuits that are implicated in a rare neurodegenerative disorder called Multiple System Atrophy (MSA). By creating this assembloid using cells derived from patients with this particular disease, it is possible to reproduce the pathology of this disease in vitro. In this study, organoids showed a correct spatial and temporal progression of the genes involved in the development of both mesencephalon and striatum, mimicking the correct neurodevelopment in vivo.

## 47. Neuroprotective Effect of a Novel Metabotropic Glutamate Receptor 3 Positive Allosteric Modulator in In Vitro Model of Parkinson’s Disease

Miriana Scordino ^1^, Monica Frinchi ^1^, Giulia Urone ^1^, Virginia Spanò ^2^, Paola Barraja ^2^, Alessandra Montalbano ^2^, Marilia Barreca ^2^, Giuseppa Mudò ^1^ and Valentina Di Liberto ^1^

^1^  University of Palermo, Department of Biomedicine, Neuroscience and Advanced Diagnostic, Palermo, Italy 

^2^  University of Palermo, STEBICEF department, Palermo, Italy

Parkinson disease (PD) is a heterogeneous disorder caused by the necrosis of dopaminergic neurons in the substantia nigra, which leads to disability in motor performance. Due to the fact that PD is characterized by a complex pathophysiology, involving among others oxidative stress and neuroinflammation, there is no cure for PD, and the current pharmacology basically aims to control only the symptoms. Therefore, the development of novel therapies for PD represents a challenge for medical research. In this context, indirect evidence strongly suggests metabotropic glutamate receptors 3 (mGluR3) as good therapeutic candidate for PD. The recent development of positive allosteric modulator (PAM) that selectively activates mGluR3 allowed us an in-depth investigation of the neuroprotective role of these receptors in an in vitro model of PD, represented by SH-SY5Y neuroblast-like cells treated with the dopaminergic neurotoxin 6-hydroxydopamine (6-OHDA).

Dose-effect investigation showed that PAM treatment has not toxic effect on cells. Interestingly, when PAM was applied alongside 6-OHDA, it rescued cell viability SH-SY5Y impaired by 6-OHDA treatment. Neuroprotective action of PAM treatment seems to be associated with the modulation of Glial cell line-derived neurotrophic factor (GDNF) expression and its receptors (RET) activation. Indeed, treatment with PAM enhanced both GDNF and RET phosphorylation levels severally impacted by 6-OHDA treatment. Finally, pilot experiments suggested that PAM neuroprotective response might be linked to the activation of the mitogen-activated protein kinase (MAPK) cascade.

These results, although preliminary, support the role of drugs that activate mGluR3 as new disease-modifying and symptomatic agents in PD.

## 48. Characterization of Spinal Cord Organoids Derived from sALS Patients

Matteo Bordoni ^1^, Eveljn Scarian ^1^ ^2^, Maria Garofalo ^3^, Maria Garau ^4^, Francesca Dragoni ^5^, Rosalinda Di Gerlando ^3^, Emanuela Jacchetti ^6^, Manuela Teresa Raimondi ^6^, Stella Gagliardi ^3^ and Orietta Pansarasa ^1^

^1^  Cellular Model and Neuroepigenetics, IRCCS Mondino Foundation, Pavia, Italy 

^2^  Department of Brain and Behavioral Sciences, University of Pavia, Pavia, Italy 

^3^  Molecular Biology and Transcriptomics, IRCCS Mondino Foundation, Pavia, Italy 

^4^  Neurogenetics Research Centre, IRCCS Mondino Foundation, Pavia, Italy 

^5^  Department of Biology and Biotechnology, University of Pavia, Pavia, Italy 

^6^  Department of Chemistry, Materials and Chemical Engineering “Giulio Natta”, Politecnico di Milano, Milan, Italy

Amyotrophic lateral sclerosis (ALS) is a non-cell autonomous disorder as many cell types contribute to motor neurons death. The lack of effective treatments is probably due to the absence of a realistic model that can recapitulate pathogenic mechanisms. Cerebral organoids are pluripotent stem cell-derived self-organizing structures that allow in vitro generation of the tissues. We developed a new method for the generation of spinal cord organoids (SCOs) that can be used for the study of pathogenic mechanisms in ALS. Aim of the work was to characterize a 3D organoid model for the study of ALS pathogenesis. We started from iPSCs obtained from healthy controls and sporadic ALS (sALS) patients. We differentiated iPSCs into neural stem cells (NSCs). We dissociated NCSs using StemPro Accutase and a cell strainer. Then, we plated NSCs on low-attachment plates and we cultured them in floating conditions using an orbital shaker. We differentiated NSCs to generate SCOs. We then characterized cells by phase-contrast and confocal microscopy. We found that SCOs derived from sALS patients were smaller and with irregular morphology compared to healthy controls. Using the GFAP marker, we found that sALS organoids have a thicker glial layer compared to healthy controls. We also found that healthy controls organoids show longer neurites compared to sALS organoids. Finally, we found a diverse composition of cell populations. Indeed, healthy controls organoids show a higher amount of differentiated cells compared to sALS organoids. We investigated cytokines released in culture supernatant of SCOs, and we found several differences between ALS patients and healthy control organoids. In particular, we reported the upregulation of ApoA1, CD30, EGF, GM-CSF, MMP-9, OPN, and ADSF/RETN and the downregulation of Ang-1, HGF, IGFBP3, IL8, MCP-1, and VCAM1. In conclusion, our data suggest that SCOs represent a promising tool for the investigation of pathogenic mechanisms of ALS.

## 49. The Loss of Frataxin Impairs Microglia Homeostatic Functions in Friedreich’s Ataxia

Martina Milani, Ilaria Della Valle, Riccardo Turchi, Flavia Tortolici, Daniele Lettieri Barbato, Katia Aquilano, Savina Apolloni and Nadia D’Ambrosi

Università degli Studi di Roma Tor Vergata, Biologia, Roma, Italy

Friedreich’s ataxia (FRDA) is a rare genetic disorder caused by mutations in the gene frataxin, encoding for a mitochondrial protein involved in iron handling and the biogenesis of iron-sulphur clusters, with consequent progressive nervous system damage. Although the overt manifestations of FRDA in the nervous system are mainly observed in neurons, alterations in non-neuronal cells may contribute to the pathogenesis of the disease, as recently suggested for other neurodegenerative disorders. In FRDA, the involvement of glial cells can be ascribed to direct effects caused by frataxin loss in these cells, eliciting different aberrant mechanisms that can concur to and exacerbate neuron loss. Recent findings obtained in FRDA patients and cellular and animal models of the disease have suggested that neuroinflammation can accompany or even be causative of the neuropathology. Thus, with this project, we aim to demonstrate that frataxin deficiency leads to an impairment of microglia homeostatic functions in FRDA. Our results demonstrate that microglia from the cerebellum of knock-in/knockout (KIKO) FRDA mice display impairment in phagocytic and migratory capabilities and dysregulation in several genes, such as gp91phox, IL-1beta, P2Y12, TREM2, and CX3CR1. Most importantly, frataxin knockdown in primary microglia alters their capacity to support the development and survival of mouse neurons, evidencing a non-cell autonomous detrimental mechanism in FRDA, where frataxin can control microglia activity. These data suggest that microglia targeting could play a valuable role in ameliorating neuronal circuits in FRDA-affected CNS regions, consistent with other neurodegenerative conditions, where the modulation of microglia represents one of the most promising therapeutic strategies.

## 50. Chronic Administration of Palmitoylethanolamide Counteracts Cognitive Decline in Tg2576 Mice

Davide Decandia ^1^, Stefano Sacchetti ^2^, Eugenia Landolfo ^3^, Greta Massa ^2^, Elena Allegretti ^2^, Giacomo Giacovazzo ^4^ and Debora Cutuli ^2^

^1^  IRCCS Fondazione Santa Lucia; Sapienza University of Rome, PhD program in Behavioral Neuroscience, Department of Psychology, Rome, Italy 

^2^  IRCCS Fondazione Santa Lucia; Sapienza University of Rome, Department of Psychology, Rome, Italy 

^3^  Sapienza University of Rome, Department of Psychology, Rome, Italy 

^4^  IRCCS Fondazione Santa Lucia, Laboratory of Neurochemistry, Rome, Italy

Palmitoylethanolamide (PEA) has been emerging as a safe and well tolerated analgesic, anti-inflammatory, and neuroprotective mediator, acting at several molecular targets in the nervous system. PEA is present in foods, such as egg yolk, corn, peanut, and soy oil. It is synthesized from lipid components of cellular membranes and can be found in high concentrations in brain tissues. In this study, we evaluated the effects of a chronic (six months) administration of ultra-micronized PEA on cognitive decline in transgenic Tg2576 (Tg) mice expressing mutant APP. When aged, Tg mice develop accumulation of amyloid peptide and amyloid plaques in the brain, as well as cognitive deficits, representing thus an animal model of AD. PEA administration was performed via a subcutaneous delivery system in Tg mice and wild-type control group (from six to 12 months of age). PEA effects on behavior were observed longitudinally in a pre-symptomatic phase (three months), a mild-symptomatic phase (6.5 months) and a full-symptomatic phase (11–12 months). Behavioral assessment was performed by using the following validated tests: Elevated Plus Maze, Rotarod Test, Y-Maze Spontaneous Alternation Test, Novel Object Recognition Test, Tail Suspension Test and Morris Water Maze. PEA administration restored the novelty recognition memory of Tg mice during the full-symptomatic phase. PEA was able to counteract hippocampal-dependent mnesic deficits, suggesting the therapeutic potential for the early treatment of AD. Further progress analyses involved histological evaluation of dendritic branching, spine number, amyloid plaques, and glial reactivity in the hippocampal CA1. This research is aimed to increase knowledge of the effects of PEA as a safe and low-cost nutraceutical tool useful to improve quality of life in AD.

## 51. Increased Apoptotic Cell Death in Riboflavin Transporter Deficiency

Chiara Marioli ^1^, Maurizio Muzzi ^1^, Fiorella Colasuonno ^2^, Valentina Magliocca ^2^, Enrico Silvio Bertini ^2^, Marco Tartaglia ^2^, Claudia Compagnucci ^2^ and Sandra Moreno ^1^

^1^  University Roma Tre, Dept. of Science, Rome, Italy 

^2^  IRCCS Children’s Research Hospital Bambino Gesù, Dept. of Neuroscience, Rome, Italy

Riboflavin transporter deficiency (RTD) is a rare, neurological disorder characterized by hearing loss and sensory ataxia associated with spinal motor neuron (MN) degeneration. The disease is caused by loss of function mutations in SLC52A2/3 genes, respectively encoding riboflavin transporters hRFT2 and hRFT3. As RF is the precursor of the coenzymes FMN and FAD, their abnormally low levels result in defective functionality of flavoproteins, which are involved in cellular bioenergetics and cell survival processes. We previously demonstrated mitochondrial and peroxisomal altered energy metabolism pathways, accompanied by cytoskeletal derangement. As this disorder lacks dependable in vivo models, we took advantage of iPSC technology to recapitulate human neuronal features of RTD. More specifically we studied MNs differentiated from patient-specific iPSCs to perform combined ultrastructural and confocal analyses, aimed at characterizing the pathomechanisms associated to RTD. Patient-specific iPSCs and iPSC-derived MNs have been analysed by focused ion beam/scanning electron microscopy (FIB/SEM) and conventional SEM. RTD cells displayed profound alterations including neurite swellings, typical neurodegeneration hallmarks suggesting impaired intracellular trafficking. Increased apoptosis was observed in RTD cells, confirmed by the presence of vesicles and blebs budding from the cell surface of RTD cells and by activated caspase-3 immunofluorescence and TUNEL assays. Consistent with these results, ultrastructural characterizations revealed aberrant mitochondrial features, confirming the persistence of mitochondrial damage after differentiation, suggesting that energy metabolism was impaired. Overall, our work contributes to the knowledge on the multiple cellular features associated to the neuronal phenotype of RTD, supporting a central role played by mitochondrial apoptosis in its pathogenesis, thus indicating potential targets for future therapies.

## 52. Towards Developing a Mass Spectrometry Assay to Identify Post-Translational Modifications of Deoxycytidine Kinase Possibly Relevant to the Response to Cladribine

Federico Carlini ^1^, Paola Barboro ^2^, Erika Iervasi ^2^, Gabriela Coronel Vargas ^2^, Marco Ponassi ^2^, Aldo Profumo ^2^, Camillo Rosano ^2^, Nicole Kerlero de Rosbo ^1^ and Antonio Uccelli ^1^

^1^  U.O. Neuroscienze Sperimentali, Ospedale Policlinico San Martino- Sistema Sanitario Regione Liguria-IRCCS, Genova, Italy

^2^  U.O. Proteomica e Spettrometria di Massa, Ospedale Policlinico San Martino- Sistema Sanitario Regione Liguria-IRCCS, Genova, Italy

Activation of cladribine (2CdA), a drug approved for multiple sclerosis (MS), is driven by a high ratio of deoxycytidine kinase (dCK)/5′nucleotidase. In view of their high dCK content, lymphocytes are preferential targets for 2CdA. We demonstrated that the 2CdA-induced apoptosis in stimulated T cells is correlated with enhanced dCK expression and activity. Phosphorylation of serine (Ser) 74 was shown to be crucial for dCK activity. However, little is known about if and/or how the other 13 phosphorylation sites described to date in dCK amino acid sequence play a role in dCK activity. Our first objective was to assess the differential phosphorylation status of dCK isoforms to understand its possible implication in dCK activity and therefore response to 2CdA treatment. We used Phos-tag™ electrophoresis, which separates proteins according to their phosphorylation status, followed by immunoblotting with a monoclonal anti-dCK antibody. The immunoreactive bands were cut out from the immunoblot and the membrane-bound protein digested with trypsin. This method allows to obtain enriched extracts of phosphorylated dCK isoforms and to reduce, at the same time, the background noise of other proteins. Tryptic digests were analyzed by mass spectrometry and yielded four different dCK peptides. Interestingly, three of these peptides contained the ATP binding site (Gly 28-Thr 36), whereas one encompassed a phosphorylation site at Ser 35). Through in silico modeling of dCK crystalized together with 2CdA (Protein Data Bank code 2ZIA), we hypothesize that Ser 35 is involved in the transfer of a phosphate group from the phosphate donor (ATP) to the substrate (2CdA). None of the peptides obtained from this preliminary experiment contained Ser 74 and other trypsinization protocols are being tested. Further analysis of dCK phosphorylation status and activity in lymphocytes from 2CdA-treated MS patients will help to predict and monitor the impact of 2CdA.

## 53. The Function of GPR183/7α,25OHC Signalling in the Brain Microvessels and Multiple Sclerosis

Fionä Caratis ^1^, Bartosz Karaszewski ^2^, Tomomi Furihata ^3^ and Aleksandra Rutkowska ^1^

^1^  Medical University of Gdańsk, Dept. of Anatomy and Neurobiology, Gdańsk, Poland

^2^  Medical University of Gdańsk & University Clinical Centre, Department of Adult Neurology, Gdańsk, Poland

^3^  Tokyo University of Pharmacy and Life Sciences, Laboratory of Clinical Pharmacy & Experimental Therapeutics, School of Pharmacy, Tokyo, Japan

The endogenous ligand for the G protein-coupled receptor GPR183 (aka EBI2) is an oxysterol 7α,25OHC. It is synthesized in vivo from cholesterol via sequential enzymatic activity of CH25H and CYP7B1 and is degraded with HSD3B7. The GPR183 receptor has chemotactic properties and upon binding with 7α,25OHC induces migration of GPR183-expressing cells in vitro and in vivo. We showed before that lipopolysaccharide (LPS)-treated mouse astrocytes release 7α,25OHC in vitro and that the astrocyte/LPS-conditioned media induces migration of macrophages. Others demonstrated that during the early stages of experimental autoimmune encephalomyelitis, the concentration of 7α,25OHC increases in the central nervous system (CNS) thus facilitating brain infiltration by GPR183-expressing immune cells. Most importantly, we and others showed increased expression of GPR183 in infiltrating lymphocytes and glial cells in multiple sclerosis (MS) plaques. Here, we investigate the expression levels of 7α,25OHC-synthesising (CH25H, CYP7B1) and degrading (HSD3B7) enzymes in MS brains and the microvasculature, and challenge the human tri-cell BBB spheroids with cerebrospinal fluid (CSF) or serum from control and MS patients. We then measure the permeability of the spheroid and migration of MS patient CD4+ cells in this model. The data showed that GPR183, CH25H, CYP7B1, and HSD3B7 enzymes are expressed in the human brain white matter and the brain microvasculature. Only the MS patient serum significantly increased the permeability of the spheroids. The spheroid permeability and chemotaxis of patient CD4+ cells in the BBB model were modulated with GPR183 ligands thus indicating GPR183-mediated signaling in CD4+ T cell migration. Taken together, the data indicate that pharmacological modulation of the GPR183/7α,25OHC signaling in the brain microvasculature may limit the entry of encephalitogenic peripheral immune cells into the CNS.

## 54. Multi-Dimensional Genome-Wide Analysis Reveals Robust Pre-Symptomatic Defects in Translation in Two SMA Mouse Models

Martina Paganin ^1^, Fabio Lauria ^2^, Ilaria Signoria ^2^, Deborah Donzel ^2^, Ewout J.N. Groen ^3^, Yu-Ting Huang ^4^, Marina Boido ^5^, Thomas H. Gillingwater ^4^, Gabriella Viero ^2^

^1^  Istituto di Biofisica, CNR, Università di Trento, Trento, Italy 

^2^  Istituto di Biofisica, CNR, Trento, Italy 

^3^  UMC Utrecht Brain Center, Department of Neurology and Neurosurgery, Utrecht, Italy 

^4^  Biomedical Sciences & Euan MacDonald Centre for Motor Neuron Disease Research, University of Edinburgh, Edinburgh, UK 

^5^  Neuroscience Institute Cavalieri Ottolenghi & University of Turin, University of Turin, Torino, Italy

Spinal muscular atrophy (SMA) is an autosomal recessive neurodegenerative disease representing the most common genetic cause of infant mortality. SMA is caused by deletions or mutations in the survival motor neuron gene (Smn1) which induces reduced levels of the SMN protein. Deficient levels of Survival Motor Neuron protein (SMN) were recently observed to lead to defective translation in primary motor and cortical neurons, and multiple tissues at an early stage of disease in the Taiwanese model of SMA. Even though structural and functional impairments in several tissues and organs are increasingly acknowledged, the impact of SMN loss in ribosome occupancy along mRNAs in diverse tissues, SMA mouse models and disease stages is still unclear. SMN protein levels in physiological conditions vary at different developmental stages and disease models, leading to the hypothesis that these translational defects may vary accordingly. We performed polysomal profiling and a multi-dimensional analysis of translational defects by obtaining and integrating ribosome profiling data in all the above-mentioned conditions. From polysomal profiling we computed the fraction of ribosome in polysomes in brain, spinal cord and liver, at pre, early and late stage of disease in the Taiwanese and Δ7 mouse model of SMA, finding a remarkable decrease of this parameter across these multiple conditions. By ribosome profiling, we identified a set of genes with altered ribosome occupancy in brain, spinal cord, and liver, at pre- and early-symptomatic stages in the Taiwanese mouse model. We also found strong translational defects in brain at the pre-symptomatic stage in the Δ7 mouse model, and observed defective biological processes shared with the Taiwanese mouse model. Our results demonstrate that the very strong variations in translation occur in SMA across multiple models at pre-symptomatic stages of disease, suggesting a new scenario in SMA pathogenesis.

## 55. Dissecting the Role of PCDH19 in Clustering Epilepsy by Exploiting Patient-Specific Models of Neurogenesis

Rossella Borghi ^1^, Valentina Magliocca ^1^, Stefania Petrini ^2^, Libenzio Adrian Conti ^2^, Sandra Moreno ^3^, Enrico Silvio Bertini ^1^, Marco Tartaglia ^1^ and Claudia Compagnucci ^1^

^1^  Bambino Gesù Children’s Research Hospital, Genetics and Rare Diseases Research Division, Rome, Italy 

^2^  Bambino Gesù Children’s Research Hospital, Confocal Microscopy Core Facility, Research Laboratories, Rome, Italy 

^3^  University “Roma Tre”, Department of Science, Rome, Italy

PCDH19-related epilepsy is a rare genetic disease caused by the defective function of PCDH19, a calcium-dependent cell–cell adhesion protein of the cadherin superfamily. This disorder is characterized by a heterogeneous phenotypic spectrum, with partial and generalized febrile convulsions that are gradually increasing in frequency. Developmental regression may occur during disease progression. Patients may present with intellectual disability (ID), behavioral problems, motor and language delay, and a low motor tone. In most cases, seizures are resistant to treatment, but their frequency decreases with age, and some patients may even become seizure-free. ID generally persists after seizure remission, making neurological abnormalities the main clinical issue in affected individuals. An effective treatment is lacking. In vitro studies using patient-derived induced pluripotent stem cells (iPSCs) reported accelerated neural differentiation as a major endophenotype associated with PCDH19 mutations. By using this in vitro model system, we show that accelerated in vitro neurogenesis is associated with a defect in the cell division plane at the neural progenitors stage. We also provide evidence that altered PCDH19 function affects proper mitotic spindle orientation. Our findings identify an altered equilibrium between symmetric versus asymmetric cell division as a previously unrecognized mechanism contributing to the pathogenesis of this rare epileptic encephalopathy.

## 56. Healthy Life-Style Approaches to Attain Disease Modification in Acquired Epilepsies

Valentina Kebede, Daniele Spallacci, Nicole Tonesi, Rossella Di Sapia and Annamaria Vezzani

Istituto Mario Negri, Neuroscience, Milan, Italy

Healthy lifestyle was reported to improve neurological outcomes after acute brain injuries. In particular, physical activity might improve neurological deficits and reduce seizures in epilepsy. Our study investigates whether aerobic and regular physical exercise reduces the risk of developing epilepsy, the burden of seizures and neuropathology following a focal brain lesion. C57BL6/N adult male mice were given free access to running wheels in their home cage for five weeks before being exposed to intra-amygdala kainate to induce a status epilepticus that leads to epilepsy development. Then, injured mice were allowed to run for six additional weeks, a time required for chronic epilepsy development. Control mice were similarly treated with kainate but left in their home cage in the absence of running wheels (sedentary mice). Sham mice were prepared for post-mortem histological analysis. Data show that running mice develop status epilepticus of reduced severity (decreased number of spikes, *p* < 0.05 vs. sedentary mice). Although epilepsy incidence and seizure frequency were not modified by running activity, mice developed spontaneous seizures of reduced duration (*p* < 0.05) vs. sedentary mice. Pyramidal neuron loss in CA1-4 hippocampal sector was similar in running vs. sedentary mice whereas GluR2/3-positive hilar mossy cell were protected in running mice (*p* < 0.05 vs. sedentary mice). Notably, seizure duration negatively correlated with the number of hilar mossy cells (r = −0.7, *p* < 0.05). Data support the beneficial effects of physical activity to improve pathologic outcomes after an epileptogenic brain insult.

## 57. Somatic Mutations and Epileptic Seizures Originating from the Contralateral Hemisphere: Two Possible Pathogenetic Mechanisms and Personalized Pharmacological Approaches

Cristiana Pelorosso, Valerio Conti, Renzo Guerrini

Children’s Hospital A. Meyer-University of Florence, Children’s Hospital A. Meyer-University of Florence, Florence, Italy

In recent years, somatic mosaicism has been demonstrated as a significant cause of neurodevelopmental disorders, and in particular of focal cortical dysplasia (FCD), which is the major cause of drug-resistant epilepsy in children. Among the different types of FCD, Type II FCD, which is the best characterized, is mainly caused by somatic mutations in mTOR pathway genes. Surgery is the only treatment option effective at controlling seizures in FCD patients. As complete as possible resection of the dysplastic tissue influences post-surgery outcome. In some cases, patients undergo multiple surgeries, which may end with a hemispherotomy, due to the persistence of seizures. Although hemispherotomy is effective in about 60–75% of cases, in some patients this surgical procedure resulted in recurrent seizures originating from the contralateral, seemingly normal, hemisphere. Here we propose two possible genetic mechanisms underlying this phenomenon. The first implies a double-hit mechanism involving two pathogenic mutations in different genes of the same pathway. One of the two mutations is confined to the dysplastic tissue and is mainly associated with the malformation phenotype, while the other is present also in the contralateral hemisphere and mainly contributes to epileptogenesis. The second mechanism is based on the possibility that the pathogenic mutation originates early during brain development, before the hemispheric cleavage, and asymmetrically distributes between the brain hemispheres. In this case, one hemisphere receives a number of mutated cells sufficient to develop an MRI-visible malformation, whereas mutant cells in the contralateral hemisphere, not sufficient in number to result in a visible malformation, would become epileptogenic only after removal of the overwhelmingly epileptogenic contralateral dysplasia. Finally, we discuss possible personalized pharmacological approaches with MTOR inhibitors to treat drug-resistant epilepsy in FCD.

## 58. Nr2f1 Haploinsufficiency Affects Immature Granule Neurons Morphology and Leads to an Altered Activation of Neuronal Ensembles within the Adult Mouse Hippocampus

Eleonora Dallorto ^1^, Sara Bonzano ^1^, Enis Hidisoglu ^2^, Alessia Pattaro ^1^, Michèle Studer ^3^, Andrea Marcantoni ^2^ and Silvia De Marchis ^1^

^1^  University of Torino, Neuroscience Institute Cavalieri Ottolenghi (NICO) & Department of Life Sciences and Systems Biology (DBIOS), Torino, Italy 

^2^  University of Torino, Department of Drug Science and Technology, Torino, Italy 

^3^  Univ. Côte d’Azur, CNRS, Inserm, Institute of Biology Valrose (iBV), Nice, France

The newly identified Bosch-Boonstra-Schaaf optic atrophy-intellectual syndrome (BBSOAS; OMIN#615722), is a rare neurodevelopmental disorder caused by mutations in the NR2F1 gene, also known as COUP-TFI, a transcriptional regulator playing pleiotropic functions in brain development. Although BBSOAS is characterized by a complex and wide array of clinical features, intellectual disability (ID) associated to global developmental delay, visual impairment, and autistic traits are the most common. Interestingly, alterations in postnatal neurogenesis and functional integration of adult-born granule neurons in the hippocampal circuit have been reported in animal models of ID and recent findings suggest that a deficit in hippocampal plasticity may contribute to BBSOAS. Here, to investigate the possible effects of Nr2f1 haploinsufficiency on the hippocampal circuit we took advantage of constitutive Nr2f1 heterozygous mice (i.e., Nr2f1-HET), a recently validated BBSOAS mouse model, and focused on the dentate gyrus (DG). Our data indicate that Nr2f1 haploinsufficiency does not alter the total number of DCX+ neuroblasts/immature neurons in the adult DG. However, these cells in Nr2f1-HET mice show atypical and peculiar neuronal morphologies, which are usually associated with pathological conditions and aberrant hippocampal circuitry activation. We thus focused on the expression of immediate early genes (e.g., Npas4, c-fos) and found that Nr2f1 heterozygous mice exhibit an increased activation of DG mature granule neurons both under basal conditions and in response to short-term exposure to a novel enriched environment. Accordingly, patch-clamp recordings of DG granule cells from hippocampal slices focusing on spontaneous GABA release showed a reduced mIPSCs frequency with no changes in mIPSCs amplitude, suggesting reduced inhibition on granule cells. Experiments are in progress to further elucidate excitatory/inhibitory imbalance in the DG of Nr2f1-HET mice.

## 59. Selective Behavioral Alterations after Acute Particulate Matter Exposure in a Presymptomatic Multiple Sclerosis Mouse Model

Martino Bonato ^1^, Francesca Montarolo ^2^, Roberta Parolisi ^1^, Claudio Pandino ^1^, Antonio Bertolotto ^2^, Annalisa Buffo ^1^ and Enrica Boda ^1^

^1^  Università degli studi di Torino, Department of Neuroscience, Turin, Italy 

^2^  Università degli studi di Torino, Neuroscience Institute Cavalieri Ottolenghi, Orbassano, Italy

Exposure to air pollution, and particularly to particulate matter (PM), has been associated with higher rates of Multiple Sclerosis (MS) relapses and increased neuroinflammation in MS patients, suggesting that PM exposure may contribute to MS exacerbation. To address this issue, we have combined the induction of MOG35-55-induced experimental autoimmune encephalomyelitis (EAE) in mice and PM10 exposure. To study the effects of both short- and long-term PM exposures, mice were exposed to PM10 at dosages relevant for human exposure either acutely, before the immunization or during the pre-symptomatic phase or chronically for seven days pre-immunization and again seven days post-immunization. Both chronic and acute PM10 exposures did not significantly modify the disease course or the neuropathology of EAE mice. Yet, few hours after exposure, EAE mice acutely exposed to PM10 during the pre-symptomatic phase (PM-EAE) showed behavioral alterations, that could not be detected neither in control EAE (Ctrl-EAE) nor in PM-exposed wild-type (PM-WT) mice. Namely, when tested in the Open Field, Elevated Plus Maze and Novel Object Recognition tests, PM-EAE mice showed reduced anxiety and a significant increase in novelty seeking. Stereotypic behaviors (i.e., grooming and rearing) instead did not appear selectively affected in PM-EAE mice. Since the observed behavioral phenotypes are frequently associated with alterations of the dopaminergic neurotransmission, along with neuroinflammation markers, we are now studying whether PM10 exposure in EAE mice is associated with changes in brain dopamine levels or in the expression of genes coding for dopamine receptors/transporters and their dynamics/recycling. Our data indicate that PM10 exposure did not alter the EAE course, probably due to the low PM10 dose used to mimic the human exposure. However, acute PM10 exposure selectively induces behavioral changes in EAE mice, possibly interacting with their altered neuroimmune/neurotransmission background.

## 60. The Spinal Cord Plasticity: Regionalization and Time-Course of Neurovascular Events Following Peripheral Nerve Injury

Ivana Allocca, Raffaella Cirillo, Giovanni Cirillo, Assunta Virtuoso, Michele Papa and Ciro De Luca

University of Campania “Luigi Vanvitelli”, Department of Public Medicine, Laboratory of Morphology of Neuronal Network, Napoli, Italy

The central nervous system (CNS) plasticity is significantly perturbed following peripheral injuries. Structural or functional alterations of the neurovascular unit (NVU) can be pivotal and early changes leading to maladaptive rewiring of the CNS. The Spared Nerve Injury (SNI) model was used to trigger a perturbation of CNS homeostasis. Rats were sacrificed at 24 h, 48 h and seven days after SNI or SHAM surgery. Immunohistochemistry, immunofluorescence, and western blot techniques were performed. Analysis of rat spinal cord sections revealed a time-dependent response that was differently modulated for microglia and astrocytes. Astrocytic pedicles (AQP4) polarization and morphology were evaluated for their involvement in the constitution of NVU together with the endothelial and basal lamina. To confirm the pleiotropy of protein usually confined to the vascular compartment, we found the overexpression of the thrombin receptor PAR-1 and the concomitant increase of the vascular endothelial growth factor (VEGF) influencing the CNS circuitry, without the direct lesion of vessels or parenchyma. Our results shed new light on the CNS maladaptive plasticity in the early phases following peripheral injury. These findings prompt further studies on NVU and novel multi-target and time-dependent therapies.

## 61. The Promoter Methylation Status, mRNA Expression and Production of TNFα, IL6 and IL10 in Multiple Sclerosis Patients

Lisa Aielli ^1^, Erica Costantini ^2^, Valentina Gatta ^3^, Fani Konstantinidou ^3^ and Marcella Reale ^1^

^1^  University “G. d’Annunzio” Chieti-Pescara, Department of Innovative Technologies in Medicine and Dentistry, Chieti, Italy 

^2^  University “G. d’Annunzio” Chieti-Pescara, Department of Medicine and Science of Aging, Chieti, Italy 

^3^  University “G. d’Annunzio” Chieti-Pescara, Department of Psychological, Health and Territorial Sciences, Chieti, Italy

Multiple sclerosis (MS) is an immune-mediated inflammatory demyelinating disease of the central nervous system and epigenetic alteration may modulate inflammatory mediators, playing a pivotal role in MS pathogenesis. To shed more light on the involvement of cytokines in MS pathogenesis, we investigated the association between cytokine production, mRNA expression, and promoter methylation status in naïve MS patients.

The promoter methylation status of tumor necrosis factor (TNF)α, interleukin (IL)6 and IL10 genes was performed on whole blood of 31 MS patients and 16 healthy controls (HC), using the pyrosequencing method. The gene expression was evaluated by Real-time PCR in peripheral blood mononuclear cells and cytokines levels were measured in serum by ELISA assay.

The average methylation index calculated by PyroMark CpG software was significantly declined for TNFα, IL6, and IL10 promoters in MS patients in comparison with HC. Our results indicate that TNFα and IL6 genes hypomethylation can predict their increased mRNA expression and production, helping to discriminate between HC and MS patients. Instead, the expression and production of anti-inflammatory IL10 were lower in MS patients than in HC and not related with its hypomethylated promoter.

Due to the complex regulation of cytokine signaling, epigenetics’ role in MS needs to be clarified by the analysis of circular RNA and microRNA (miRNA) as epigenetic modulators. Preliminarily, we observed a significant downregulation of miRNA-124 in MS patients compared to HC, consistent with the support of inflammation. Thus, further research in larger groups of patients and on additional epigenetic regulators is ongoing to better elucidate the functions of epigenetic modifications in MS pathogenesis, progression, and activity.

## 62. Neuroinflammation in Fabry’s Disease: A New Insight into a Multisystemic Disease

Francesca Massenzio ^1^, Cecilia Delprete ^1^, Serena Aleo ^1^, Barbara Monti ^1^, Rocco Liguori ^2^, Anna Maria Ghelli ^1^, Roberto Rimondini-Giorgini ^3^ and Marco Caprini ^1^

^1^ University of Bologna, Department of Pharmacy and Biotechnology, Bologna, Italy 

^2^ University of Bologna, Department of Biomedical and Neuromotor Sciences, Bologna, Italy

^3^ University of Bologna, Department of Medical and Surgical Sciences, Bologna, Italy 

Fabry’s disease (FD) is a rare X-linked lysosomal storage disease characterized by defective lysosomal enzyme α-galactosidase A (α-Gal), leading to multisystemic manifestations due to the accumulation of the globotriaosylceramide (Gb3). A hallmark symptom of FD patients is neuropathic pain that appears in the early stage of the disease, as a result of peripheral small fibres damage and central nervous system disease. Evidence regarding the extent of neurocognitive impairment is still limited, even though different forms of cognitive dysfunctions have been recently reported in FD patients.

Cognitive impairment is commonly accompanied by neurodegenerative and mental disorders, in which the neuronal dysfunction is probably induced by an excessive inflammatory response. To clarify whether a neuroinflammatory process could be observed in FD animal model, we studied the level of neuroinflammatory markers in 3–4 month-old and 12 month-old α-Gal A KO mouse.

We observed an increase in the pro-inflammatory marker IL-1β in both cortex and hippocampus, together with an increasing trend in the expression of TNF-α in the cortex of 12 months-old α-Gal KO mice compared to WT ones. This observation could indicate the presence of an inflammatory process in 12-month FD since the expression of the abovementioned markers was not detected in young mice. Moreover, the reconstruction analysis of microglia morphology showed a decrease in the ramification of microglia in 12 months-old α-Gal A KO mice compared to WT animals, compatible with an activated phenotype in response to inflammatory stimuli.

Taken together, this evidence could indicate an inflammatory response driven by microglial cells toward a pro-inflammatory phenotype although the ameboid morphology has been preserved by confirming the heterogenicity of the cell population.

## 63. Time-Dependent Modifications in Glia Cells, Macrophages and Extracellular Matrix Supporting Glioblastoma Progression

Raffaella Cirillo, Ivana Allocca, Ivana Allocca, Immacolata Viscovo, Immacolata Viscovo, Ciro De Luca, Ciro De Luca, Michele Papa, Michele Papa, Assunta Virtuoso and Assunta Virtuoso

University of Campania Luigi Vanvitelli, Department of Mental and Physical Health and Preventive Medicine, Napoli, Italy

Glioblastoma (GBM) is the most common malignant brain tumor. GBM progression is promoted by intricate and dynamic crosstalk with resident glia cells, which undergo several molecular and morphological changes also reflected as extracellular matrix (ECM) modifications. To better understand GBM’s mechanism of action and its effects on the tumor microenvironment, context- and disease stage-specific molecular targets should be determined.

GL261 glioma cells were injected into the right striatum of immuno-competent C57Bl6J mice and the brains extracted after seven, 14, and 21 days (7D, 14D, 21D). Immunohistochemistry and western blotting analysis were conducted. In the early stage, the GBM growth was barely boosted as showed by spotted ki67+ cells distribution and moderate reactive astrogliosis (GFAP+, glial fibrillary acidic protein), which was progressively enhanced at later phases. The tumor bulk was established at 14D and infiltration of phagocytic cells (CD68+: Iba1+) was detected in the peritumoral area, suggesting their functional role in the ECM remodelling. Among the ECM modifiers, metalloproteinase-9, tenascin-C, and fibulin-2 increased at this stage, indicative of a selective re-organization of the ECM. Surprisingly, microglia and macrophages were scarcely localized at the site of injection with a decreased expression of monocyte chemoattractant protein-1 (MCP-1/CCL2) and Iba1, which increased later. Despite the number of Iba1-positive cells at 21D, the microglia (TMEM119+ cells) response appeared inhibited, suggesting a differential regulation for tumor-associated microglia and peripheral macrophages during GBM progression. Analyzing the glioblastoma time course and its interplay with resident glia cells may provide key information to reveal the cellular mechanisms that regulate the tumor development, encouraging a proper multi-targeted approach that could be translated to the human disease.

## 64. MiR-142-3p Is a Critical Modulator of TNF-Mediated Neuronal Toxicity in Multiple Sclerosis

Sara Balletta ^1^, Silvia Caioli ^2^, Francesca De Vito ^2^, Alessandra Musella ^3^, Diego Fresegna ^3^, Valentina Vanni ^3^, Livia Guadalupi ^3^, Francesca Romana Rizzo ^1^, Krizia Sanna ^1^, Antonietta Gentile ^3^, Diego Centonze ^2^, Georgia Mandolesi ^3^

^1^  Tor Vergata University, Department of Systems Medicine, Rome, Italy 

^2^  IRCCS Neuromed, Unit of Neurology, Pozzilli, Italy 

^3^  IRCCS San Raffaele Roma, Synaptic Immunopathology Lab, Rome, Italy

Multiple sclerosis (MS) is the main neurodegenerative autoimmune disease of the central nervous system (CNS) in young adults. In MS and its mouse model experimental autoimmune encephalomyelitis (EAE), proinflammatory cytokines trigger ‘synaptopathy’, an early and potentially reversible synaptic dysfunction that promotes excitotoxic damage. Therefore, the interest in identifying synaptotoxic biomarkers and inflammatory molecular axis is increasingly emerging to dampen excitotoxicity in MS. Notably, tumor necrosis factor (TNF) stimulation is strictly responsible for synaptic alterations and neuronal damage in both MS and EAE, but the underlying molecular mechanism is still unclear.

Small noncoding microRNAs (miRNAs) are new modulators of gene expression circulating in the cerebrospinal fluids (CSF), recently proposed as diagnostic and prognostic biomarkers for MS.

Here, we investigated miR-142-3p, a synaptotoxic microRNA induced by inflammation in EAE/MS, as a potential downstream effector of TNF-signaling. High levels of both TNF and miR-142-3p were detected in the EAE striatum and in MS CSF, and patients with elevated CSF levels of both molecules present the most unfavorable progression index. Electrophysiological recordings in EAE mice, supported by histochemical and molecular analyses, show that low miR-142-3p levels in the inflamed striatum of miR-142 heterozygous mice impaired TNF-dependent synaptotoxicity. Accordingly, TNF treatment was ineffective in healthy striatal slices incubated with LNA-anti miR-142-3p. However, both preclinical and clinical data suggested that miR-142-3p levels are independent of TNF increase. To clarify these results, we investigated a new neuronal permissive role for miR-142-3p able to modulate TNF-mediated synaptotoxicity. In conclusion, we propose miR-142-3p as a critical modulator of TNF-mediated neuronal toxicity in neuroinflammatory conditions and highlight the potentiality of anti-miR-142-3p therapy.

## 65. Stavudine “Interferes” via alpha-7nAChR to Inhibit NLRP3 in (LPS+Amyloid-Beta) Stimulated PBMC of AD Patients

Francesca La Rosa ^1^, Elisa Conti ^2^, Elisa Conti ^2^, Marina Saresella ^1^, Marina Saresella ^1^, Federica Piancone ^1^, Federica Piancone ^1^, Chiara Fenoglio ^3^, Chaiara Fenoglo ^3^, Mario Clerici ^4^, Mario Clerici ^4^

^1^  IRCCS Fondazione Don Carlo Gnocchi-Onlus, Laboratorio di biologia molecolare e traslazionale, Milano, Italy 

^2^  Laboratory of Neurobiology, School of Medicine and Surgery, Milan Center for Neuroscience, University of Milano-Bicocca, University of Miano-Bicocca, Monza Brianza, Italy 

^3^  Department of Pathophysiology and Transplantation University of Milan IRCCS Ospedale Maggiore Policlinico, Ospedale maggiore milano, Via F.Sforza 35, Milan, Italy 

^4^  Department Head, Department of of Pathophysiology and Transplantation and Chair of Immunology and Immunopathology, University of Milan, Milano, Italy

Alzheimer’s disease (AD) is marked by neuroinflammation, cholinergic hypofunction, and decreased nicotinic acetylcholine receptor (nAChR) density from the cortex and hippocampus. α7nAChR firstly identified in the autonomous nervous system, is a ligand-gated ion channel exerted as a regulator in cognitive processes through the modulation of specific neurotransmitters. Cholinergic neurons and more recently resident macrophages of different tissue proved to highly express α7nAChRs and the activation of these receptors inhibits inflammasome-NLRP3 assembly by arrestin-beta1 (ARRB1) and the production of cytokines thereby attenuating the local inflammatory response suggesting that α7nAChR activation represents a useful therapeutic strategy for AD. The current study was performed to investigate the effects of Stavudine (D4T), an antiviral drug normally used for HIV-treatment, to modulate inflammation through α7nAChR in in vitro assay of cultured PBMC of 15 AD patients compared to 12 sex and age matched healthy control (HC). PBMC of all subjects enrolled in the study were cultured in unstimulated (MED) or primed with LPS (1 µg/mL) for 2 h and Aβ (10 µg/mL) for 24h in presence/absence of D4T (50 µM) for 22h to evaluate: (1)TNF-α, IL-1β, IL-6, IL-18 and caspase-1 protein production detected by ELISA in supernatants of cells; (2) detection of ACht and anti-Ab42 antibodies levels in plasma of AD patients and HC by ELISA; (3) α7nAChR, TNF-a, IL-1β, IL-6, IL-18, Nlrp3, ARRB1, and caspase-1 mRNA expression by qPCR. The efficacy of D4T to damper NLRP3 was previously demonstrated in in vitro cells line experiments, herein data confirmed that in PBMC Stavudine reduces: (1) down-stream inflammasome derived protein production (IL-18, caspase-1 and IL-1β) and inflammatory cytokines (IL-6 and TNF-a) both in AD and HC (*p* < 0.001); (2) α7nAChR and ARBB1 mRNA only in HC with the same production of plasmatic Acth both for AD and HC. Cholinergic neurons and more recently resident macrophages of different tissue proved to highly express α7nAChRs and the activation of these receptors inhibits the production of inflammatory cytokines, thereby attenuating the local inflammatory response, suggesting that α7nAChR activation represents a useful therapeutic strategy for AD.

## 66. Characterization of Astrocyte Reactivity in a Model of Encephalopathy of Prematurity

Ariane Heydari Olya, Jennifer Hua, Estelle Nicolas, Pierre Gressens and Juliette Van Steenwinckel

INSERM U1141 Neurodiderot, Hôpital Robert Debré, Paris, France

Premature birth caused by maternal infection represent an increased risk factor of brain lesions affecting both developing gray and white matter, known as encephalopathy of prematurity (EoP), and long-term neurodevelopmental disorders. It has been suggested that the set-up of a pro-inflammatory environment with the secretion of cytokines and chemokines might initiate an inappropriate inflammatory response driven by reactive microglia and astrocytes, which participate to neurodevelopmental disruption. Astrocytes, located at the interface between the brain parenchyma and the blood brain barrier, preserve homeostasis. They also participate in the inflammatory response and go through morphological and functional changes called astrogliosis. However, little is known about astrocytic reactivity during perinatal inflammation. Our team has developed a mouse model of EoP based on systemic injections of IL-1beta (a pro-inflammatory cytokine) reproducing the deficits seen in premature infants. Using this model, we aim to precisely characterize astrocyte reactivity in EoP. Astrocyte subpopulations are highlighted using flow cytometry, emphasizing their heterogeneity. Bulk RNA sequencing of purified astrocytes showed a transcriptomic signature of astrocytes during perinatal inflammation. Analysis of A1/A2 phenotypes by quantitative RT-PCR revealed a pro-inflammatory phenotype of the astrocytic response along time. Significant morphological changes of GFAP+ astrocytes in the subventricular zone have been shown by immunohistochemistry. Functional differences have been studied by quantitative RT-PCR and revealed a decrease of synaptogenesis factors secreted by astrocytes. This in-depth characterization of astrocytes will pave the way for designing new strategies to restore the homeostatic functions of astrocytes and protect the brain of preterm infants.

## 67. Microglia-Derived Extracellular Vesicles Are Involved in Synaptic Pruning In Vitro

Giulia D’Arrigo ^1^, Giulia Cutugno ^1^, Marta Lombardi ^1^, Marta Tiffany Lombardo ^1^, Maria Teresa Golia ^1^, Francesca Sironi ^2^, Roberta Ghidoni ^3^, Caterina Bendotti ^2^, Martina Gabrielli ^1^, Claudia Verderio ^1^

^1^  National Research Council of Italy, Institute of Neuroscience, Milan, Italy 

^2^  IRCCS “Mario Negri” Institute for Pharmacological Research, Department of Neuroscience, Milan, Italy 

^3^  IRCCS Fatebenefratelli, Istituto Centro San Giovanni di Dio, Brescia, Italy

During development, microglia is responsible for beneficial synaptic pruning by phagocytosis of aberrant synapses tagged by complement factors. Moreover, excessive complement-mediated synaptic pruning is activated during neurodegeneration, causing pathological loss of synapses. However, how complement factors are delivered to synapses is not yet completely clear.

Extracellular vesicles (EVs) released by microglia carry multiple signals implicated in synaptic pruning and scan the neuron surface before establishing a stable contact with dendrites, thus representing ideal vehicles to tag synapses with molecules guiding microglial removal.

To investigate the role of microglial EVs in synaptic pruning, we cocultured mature hippocampal neurons with wild type (wt) microglia or C9orf72 knock out (ko) microglia, which produce a double amount of EVs and more complement factors (C1q/C3) associated to EVs compared to wt cells.

While wt microglia induced a decrease in the density of Shank-2-positive (postsynaptic) but not Bassoon (presynaptic) puncta, C9orf72 ko microglia reduced the density of both Bassoon and Shank-2-positive puncta in neurons. Parallel quantification of synaptic puncta in wt and C9orf72 ko microglia revealed an increased uptake of pre-synaptic markers in C9orf72 ko microglia compared to wt cells.

Importantly, pretreatment of C9orf72 ko microglia with GW4869, an inhibitor of EV biogenesis that reduces EV production by 50%, restored normal pre-synaptic density in microglia-neuron cocultures, while addition of microglial EVs to neurons before co-culturing with wt microglia induced a selective decrease in Bassoon-positive puncta, leaving the density of post-synaptic puncta unchanged.

Our results indicate that microglial EVs promote pre-synapses engulfment, thus possibly influencing the synaptic density during developmental critical periods as well in brain pathologies.

Analysis of synaptic density in hippocampi of C9orf72 are ongoing to validate in vivo microglial EVs-mediated pre-synaptic pruning.

## 68. Pro-Resolving Lipid Mediator Neuroprotectin D1 Ameliorates Chronic Experimental Autoimmune Encephalomyelitis by Modulating Macrophage Plasticity and Polarization

Alessandro Matteocci ^1^, Diego Fresegna ^2,3^, Francesca De Vito ^4^, Vladimiro Batocchi ^1^, Federico Fazio ^1^, Diego Centonze ^3,4^, Valerio Chiurchiù ^1,5^

^1^  Laboratory of Resolution of Neuroinflammation, IRCCS Santa Lucia Foundation, Rome, Italy 

^2^  IRCCS San Raffaele Pisana, Rome, Italy 

^3^  Università degli Studi di Roma Tor Vergata, Rome, Italy. 

^4^  IRCCS Neuromed, Pozzilli, Italy 

^5^  Institute of Translational Pharmacology, CNR, Rome, Italy

Multiple sclerosis (MS) is a chronic inflammatory neurodegenerative disease associated to uncontrolled inflammation and autoimmunity and for which there is still an unmet need for new diagnostic and therapeutic options. Recent studies suggest that chronic inflammation can be a consequence of failure to resolve inflammation, the resolution of which is mediated by a superfamily of bioactive lipids mainly derived from omega-3 essential fatty acids and termed specialized pro-resolving lipid mediators (SPMs). Since by means of targeted-metabololipidomics we previously found a significant impaired production of several SPMs, including neuroprotectin D1 (NPD1), in peripheral blood of MS patients, herein, we assessed its in vivo role in modulating the plasticity and activation of monocyte-derived macrophages, key cells involved in the immunopathogenesis of MS. To do so, acute and chronic experimental autoimmune encephalomyelitis (EAE) were induced in mice, and these were daily injected with NPD1 in both clinical phases. M1-like and M2-like macrophages were differentiated from monocytes obtained from spleen of acute and chronic vehicle- or NPD1-treated EAE mice and analyzed by high-dimensional flow cytometry for their signature activation markers. Although NPD1 could not impact on clinical symptoms at the peak of the disease (20–22 dpi), it significantly ameliorated disease course and severity at the chronic phase (35 dpi) and this was associated to significant changes in signature M1-like (CD86, MHC-II, CD62L, CD40 and CD68) and M2-like (Trem2, CD11c, CD44 and CD200R) markers, with an M1-to-M2 phenotypic shift in NPD1-treated mice. Altogether, these findings provide compelling evidence that boosting resolution of inflammation with a specific pro-resolving agent, could ameliorate the chronic course of MS by reprogramming macrophages plasticity and polarization and converting them into a protective and pro-resolving phenotype.

## 69. From Anatomy to Functional Connectivity in the Mouse Brain Assessed through Assembly Detection Methods

Giulia Arena, Fabrizio Londei, Francesco Ceccarelli and Aldo Genovesio

Sapienza University of Rome, Department of Physiology and Pharmacology, Roma, Italy

The concept of cell assembly as a unit of neural processing stretches back about 70 years. To date, many definitions have been provided, all of them converging on the concept of cell assembly as a functional network of neurons that fire with a defined temporal pattern and has information coding properties. We thus embarked on a series of studies using cell assemblies as a tool to investigate several aspects of mouse brain functioning, starting from the functional connectivity of a wide variety of brain areas with a magnified temporal and spatial resolution and ending, in future, in the analysis of assembly encoding of several task variables. In our studies, we used an algorithm for cell assembly detection that allows, for each assembly, the selection of the optimal bin and lag, enabling us to identify neural assemblies spanning more than 60 cortical and subcortical areas of the mouse brain in an available dataset of recordings. In the first study, we focused on the Zona Incerta (ZI) that establishes broad anatomical connectivity and through its outputs exerts mixed excitatory or inhibitory effects on many targets. This led us to expect a diverse profile of probability to detect assemblies which, being verified by our results, corroborate the biological realism of this method as a valid tool to qualitatively investigate interactions between areas. Beside confirming functional connections between anatomically connected areas and assessing their relative weight, our results led us to speculate about functional connections that may be underlined by structural connections yet unexplored. In a second study, following the validation offered by the first one, we focused on the hippocampal formation and the prefrontal cortex, objects of keen interest for their cognitive relevance, and explored their functional connections, providing a rich database of assembly distribution that can lay a solid foundation for future studies that will correlate assembly dynamics with behavior.

## 70. Mir-34a Selectively Modulates GABAergic Activation within Dorsal Raphe Nuclei in Response to Stressful but Not Rewarding Stimuli

Donald Ielpo ^1^, Serafina Manila Guzzo ^2^, Gianfranco Porcheddu ^3^, Luisa Lo Iacono ^1^, Cristina Marchetti ^3^, Carlo Cifani ^2^, Tiziana Pascucci ^4^, Rossella Ventura ^4^ and Diego Andolina ^4^

^1^  Fondazione Santa Lucia IRCCS, Fondazione Santa Lucia IRCCS, Rome, Italy 

^2^  University of Camerino, Pharmacology Unit, Camerino, Italy 

^3^  Ebri Foundation, Ebri Foundation, Rome, Italy 

^4^  La Sapienza University, Psychology Department, Rome, Italy

The dorsal raphe nuclei are the main source of serotonergic innervation in the brain. Within the DRN, GABAergic interneurons locally regulate 5-HT release in target structures, in response to external stimuli. Interestingly, similar physiological activation of DRN GABAergic neurons is observed in response to either stressful or rewarding stimuli, resulting however in very different behavioral responses, thus suggesting the presence of a diverse nature of DRN GABAergic neuronal control. However, the molecular signatures characterizing and regulating stimulus-specific GABAergic neurons in the DRN are unknown. We have reported that microRNA34a (miR-34a) is highly expressed in the DRN and plays a role in mediating behavioral and neurochemical alterations induced by stressful events.

Here, we hypothesize that miR-34a could selectively modulate GABAergic acute response to stressful but not to rewarding stimuli in the DRN.

Using histological, molecular, and genetic approaches, we first examined miR-34a cellular localization in the DRN. Then, by in vivo microdialysis and ex vivo patch clamp recording, we evaluated if miR-34a regulates acute stress and food-related GABAergic transmission in DRN.

Within the DRN, miR-34a seems to be specifically expressed in GABAergic neurons. Here, the exposure to either acute stress or palatable food similarly causes the inhibition of GABAergic activity in control mice. Notably, by two complementary pharmacological and genetic strategies, we show that the blockade of GABAergic miR-34 in the DRN enhanced local GABAergic activity, by means of local GABA release and mIPSCs frequency recorded in in 5HT-positive neurons, in response to acute stress but not to palatable food.

This study proposes miR-34a as a selective GABAergic regulator of the DRN activity, involved in the response to stressful but not rewarding stimuli. We thus propose miR-34a as a molecular signature characterizing stress-responsive GABAergic neurons in the DRN.

## 71. Alterations of Cholesterol Metabolism in Experimental Models of Rett Syndrome

Cecilia Cabasino, Elena Albizzati, Martina Breccia, Elena Florio, Nicoletta Landsberger and Angelisa Frasca

Università degli Studi di Milano, Dipartimento di Biotecnologie Mediche e Medicina Traslazionale, Segrate, Italy

Rett syndrome (RTT) is an X-linked neurodevelopmental disorder, representing the most common genetic cause of severe intellectual disability in girls. It is caused by mutations in the epigenetic factor methyl-CpG-binding protein 2 (MeCP2), mainly affecting the central nervous system. Besides the well-known neuronal and synaptic alterations, perturbed lipid metabolism has been reported both in brain and peripheral tissues of RTT mouse models and patients. Of relevance, cholesterol is involved in brain primary functions such as synaptogenesis and neurotransmission, and its alterations are associated with several neurological disorders. Since peripheral cholesterol cannot cross the blood–brain barrier, a biosynthetic pathway within the brain is necessary to maintain its physiological levels and astrocytes are thought to produce most of brain cholesterol. To determine if cholesterol metabolism is defective in RTT, we started characterizing the brain cholesterol biosynthetic pathway both in cultured astrocytes derived from Mecp2 null mice and in brain areas collected from Mecp2 deficient animals. By exploiting our RNA-Seq data we uncovered a deregulation of cholesterol pathway in Mecp2 null models, further confirmed by qRT-PCR. In particular, we observed a downregulation of cholesterol associated genes in cortex and hippocampus of symptomatic Mecp2 null animals and cultured astrocytes. Specifically, Nsdhl, an essential enzyme in cholesterol synthesis, resulted strongly downregulated both in the mRNA and in the protein levels. To analyze if these alterations result in a functional defect, we will measure cholesterol levels in brain areas of Mecp2 null mice and in cultured primary astrocytes, and we will uncover the effects of cholesterol supplementation on the synaptic phenotype. The final aim of our research is to determine if targeting the cholesterol metabolism pathway might be a possible therapeutic approach for treating RTT.

## 72. Mothers and Sons: How Bisphenols Target Brain and Behaviors

Brigitta Bonaldo ^1^, Antonino Casile ^2^, Laura Gioiosa ^3^, Martina Bettarelli ^4^, Marialaura Teresa Ostuni ^4^, Sofia Nasini ^5^, Stefano Gotti ^1^, Marilena Marraudino ^1^ and GianCarlo Panzica ^1^

^1^  Neuroscience Institute Cavalieri Ottolenghi and University of Turin, Neuroscience, Torino, Italy 

^2^  University of Camerino, Chemical and Pharmaceutical Sciences and Biotechnology, Camerino, Italy 

^3^  University of Parma, Medicine and Surgery, Unit of Neuroscience, Parma, Italy 

^4^  Neuroscience Institute Cavalieri Ottolenghi, NICO, Orbassano, Italy 

^5^  University of Padua, Pharmaceutical and Pharmacological Sciences, Padova, Italy

Bisphenols (BPs), synthetic compounds used in the production of plastics, are an extremely abundant class of endocrine disrupting chemicals, i.e., exogenous chemicals, or mixtures of chemicals, that can interfere with any aspect of hormone action. The first and still the most globally produced BP is the bisphenol A (BPA). Stricter regulations and increasing concerns about its impact on human health have led to an extensive search for safe alternatives. Bisphenol S (BPS) is one of the most used BPA substitutes, however, it seems to display the same, or even worse, endocrine disrupting properties as BPA. Interestingly, brain and behaviors appear to be targets of both BPA and BPS, particularly relevant when the exposure occurs during critical periods of development of adult life. Thus, this study aimed to evaluate the effects of oral exposure, either during pregnancy and lactation or during the perinatal period, to low dose (i.e., 4 µg/kg BW/day, EFSA TDI for BPA) BPA or BPS in mice. Dams were orally treated from mating to offspring weaning. Within the first postnatal week, we observed spontaneous maternal behavior. Finally, we analyzed the oxytocin immunoreactivity in the hypothalamic magnocellular nuclei, known to be involved in the control of maternal care. In the perinatally-exposed adult offspring, we evaluated the anxiety-related behaviors and the serotonin immunoreactivity in dorsal (DR) and median (MnR) raphe nuclei, which are highly involved in the control of these behaviors. Both BPs affected the sex ratio and the offspring mortality. Treated dams displayed impaired maternal behavior, but only the BPA-ones also showed alterations in the oxytocin immunoreactivity. In adult offspring, we observed different effects of the BPs exposure on anxiety-related behavior in males (anxiolytic) and females (anxiogenic), along with alterations of serotonin immunoreactivity in the analyzed nuclei. Both adult and perinatal periods are sensitive to BPs’ exposure which can lead to impairment in fundamental behaviors and related neural circuits.

## 73. Sexually Dimorphic Organizational Role of Estrogen Receptors on Different Neuroendocrine Systems Controlling Metabolism and Reproduction

Marilena Marraudino ^1^, Brigitta Bonaldo ^1^, GianCarlo Panzica ^1^, Paloma Collado ^2^, Helena Pinos ^2^, Stefano Gotti ^1^

^1^  NICO, Department of Neuroscience ‘Rita Levi Montalcini’, Turin, Italy 

^2^  Universidad Nacionald de Educacion a Distancia, Department of Psychobiology, Madrid, Spain

Many hypothalamic systems, controlling metabolism and reproduction, are programmed and stabilized during critical periods of development by many factors, including gonadal steroids. In particular, estradiol (E2) appears to have an important role in the organization of these circuits. E2 acts through three different receptors: Erα, ERβ, and GPR30.

To understand the role of these receptors in the organizational effects of E2, we treated male and female CD1 mice from post-natal day (PND) 5 to PND12 with subcutaneous injections of vehicle (corn oil), E2 and E2 associated with selective antagonist of estrogen receptors (MPP; PHTPP; G15) alone or together (mix). We analyzed, during the development, different physiological parameters related to food intake (body weight, food eaten, daily feed efficiency, gonadal and brown fat), reproduction (gonads, puberty onset, estrus cycle) and behavior (Y-maze, sexual behavior). Furthermore, in the adult, we have immunohistochemically highlighted the expression of some hypothalamic neuronal circuits closely associated with food-intake and metabolism, but also with the reproductive sphere: Pro-opiomelanocortin (POMC), Neuropetide Y (NPY), Orexin, and Kisspeptin systems.

In general, E2 induced effects mostly in females both on sexual and feeding behaviors. The treatments with G15 alone or in combination (mix) altered all the considered parameters in both sexes. On the contrary MPP and PHTPP showed sexually dimorphic effects. MPP modified, in males, feeding parameters, but not those related to reproduction, whereas PHTPP modified parameters related to reproduction, but not those related to feeding. In females the situation was exactly the reverse. In conclusion, our data demonstrate that E2 has a strong organizational role in different neuroendocrine systems, acting primarily on GPR30 and, in a sexually different way, on ERα and ERβ.

## 74. Addressing the Significance of Tumor-Released Microvesicles in Glioblastoma Aggressiveness and Invasion

Valentino Ribecco ^1^, Matteo Tamborini ^2^, Elisabetta Stanzani ^1^, Marco Pizzocri ^1^, Milena Mattioli ^1^, Federico Pessina ^3^, Michela Matteoli ^1^, Lorena Passoni ^1^

^1^  IRCCS Humanitas Research Hospital, Laboratory of Pharmacology and Brain Pathology, via Manzoni 56, 20089 Rozzano, Milano, Italy 

^2^  CNR Institute of Neuroscience, URT c/o Humanitas Clinical and Research Center, Laboratory of Pharmacology and Brain Pathology, via Manzoni 56, 20089 Rozzano, Milano, Italy 

^3^  IRCCS Humanitas Research Hospital, Department of Neurosurgery, via Manzoni 56, 20089 Rozzano, Milano, Italy

Glioblastoma (GBM) is the most aggressive primary brain tumor associated with a very poor prognosis. Intra-tumor heterogeneity remains a substantial barrier as it prevents the efficacy of pharmacological therapies. The presence of GBM stem-like cells (GSCs) with varying degrees of stemness heavily impacts on treatment success rates. Spatially heterogeneity is defined from the core to the edge of the tumor. Therefore, paired GSC-core and -edge primary cell lines were investigated for invasiveness. To promote tumor invasion GBM makes use of different communication routes with the neighbor environment, which include extracellular vesicles (EVs). Indeed, evidence reported in the literature suggest the involvement of the EVs released by GSCs in inducing cellular migration. EVs are mainly divided into exosomes (EXOs) and microvesicles (MVs). The migration capacity of EXOs and MVs released by both GSCs-core and GSCs-edge were investigated. Both GSC-core and -edge displayed a significant higher migratory capacity when incubated in the presence of MVs, whereas no effects were observed in the presence of EXOs. Overall, a higher significant migratory potential of the EVs derived from GSCs-edge compared to the ones derived from GSCs-core was observed. To assess the contribution of the EVs released by non-tumoral cells present in the tumor microenvironment, paired GSC-core and -edge were treated with EXOs and MVs derived from the surgical aspirate. MVs from surgical aspirate demonstrated to be more powerful than EXOs, suggesting that not only EVs from tumoral cells but also EVs from the stroma (neurons, glial and immune cells) are actively implicated in GSC mobility in vivo. These findings show widespread heterogeneity between core and edge GSC cell lines, as well as reciprocal intercellular communication via tumor and microenvironment-derived MVs, increasing invasiveness.

## 75. Relationship between Fatigue, Disability, and Reserve in Patients with MS: A Cross-Sectional and Longitudinal Analysis

Alessandra Scaravilli ^1^, Mario Tranfa ^1^, Valentina Iuzzolino ^2^, Pier Paolo Perrella ^1^, Antonio Carotenuto ^2^, Giuseppe Pontillo ^3^, Marcello Moccia ^2^, Sirio Cocozza ^1^, Andrea Elefante ^1^, Roberta Lanzillo ^2^, Arturo Brunetti ^1^, Vincenzo Brescia Morra ^2^, Maria Petracca ^2^

^1^  University of Naples ‘Federico II’, Naples, Italy, Departments of Advanced Biomedical Sciences, Naples, Italy 

^2^  University of Naples “Federico II”, Naples, Italy, Department of Neurosciences and Reproductive and Odontostomatological Sciences, Naples, Italy 

^3^  University of Naples ‘Federico II’, Naples, Italy, Departments of Advanced Biomedical Sciences and Electrical Engineering and Information Technology, Naples, Italy

Background and Aims: Fatigue is among most debilitating and common symptoms in multiple sclerosis (MS). Here, we hypothesized that individual resilience could affect motor and cognitive fatigue in MS patients, as already described for cognitive and motor disability, and explored the impact of clinico-demographic features and brain structural damage on fatigue.

Methods: Fifty-four MS patients were prospectively enrolled and underwent clinical examination (including Expanded Disability Status Scale-EDSS, Symbol Digit Modalities Test-SDMT and Beck Depression Inventory II-BDI) and MRI acquisition at baseline and after a mean follow-up of 14 months. Physical and cognitive MS-related fatigue was evaluated with the respective Modified Fatigue Impact (MFIS) subscales (MFIS-P and MFIS-C). Structural brain damage was estimated as white matter (WM) lesion load (JIM 6.0) and brain volume (SIENAX and SIENA). Percent change over time (%c) for clinical and MRI variables were also computed. A cognitive reserve index (CRI) was estimated by combining educational level, premorbid IQ and the participation in cognitive leisure activities. Brain reserve was expressed as sex adjusted intracranial volume (ICV). The association between putative risk factors (age, gender, phenotype, EDSS, SDMT-z, BDI, log transformed WM lesion load and normalized brain volume-NBV, brain reserve, cognitive reserve) and fatigue scores was assessed using bivariate correlations (preliminary screening) and hierarchical linear regressions. To explore the impact of risk factors on fatigue changes over time, partial correlations were tested between baseline features, their %c and fatigue scores %c, accounting for follow-up interval.

Results: At the cross-sectional analysis, MFIS-P was correlated with age, EDSS, BDI, NBV (r ranging from 0.01 to 0.001), but only marginally with brain reserve (*p* = 0.06). The full regression model accounted for 32% of the variance in MFIS-P (*p* = 0.001). The only variable accounting for significant variance was BDI (*p* < 0.001). MFIS-C was correlated with BDI (*p* < 0.001). As per the longitudinal analysis, none of the baseline features was associated to MFIS-P and MFIS-C %c. BDI %c was associated to MFIS-P and MFIS-C %c (r = 0.55, *p* < 0.001; r = 0.57, *p* < 0.001).

Conclusions: Among the explored features, only depression was strongly associated to both physical and cognitive fatigue. Brain and cognitive reserve did not affect fatigue symptoms in MS patients.

## 76. A Systematic Review of M-EEG Evidence on Value-Based Decisions in Humans: Experimental Paradigms and Spatiotemporal Characteristics

Isabella Colic and Jiaxiang Zhang

Cardiff University, School of Psychology, Cardiff, UK

Decision-making is an integrative process that is crucial for humans and animals on a daily basis, and it has been at the center of a long tradition of psychological and economic research. This systematic review focuses on the spatiotemporal dynamics of value-based decision-making. Since there is an abundance of fMRI, neuropsychological, and animal studies on the topic, we instead examined evidence collected from 100 magnetoencephalography and electroencephalography studies, which are known for their high temporal resolution. Additionally, we classified value-based decisions into ‘external’ (EDM) and ‘internal’ (IDM) and used this division as the theoretical framework of the present review. In ‘external’ decisions, the values of different options are objectively defined, whereas said values in ‘internal’ decisions are defined by the individual. The review aims to assess whether there is a convergent pattern of event-related potentials or fields (ERP/ERF) findings and how EDM and IDM processes have been studied so far with these methods. Based on precedents in the literature, we extracted statistically significant time intervals that result from contrasts between experimental conditions that are sensitive to differences in (external or internal) value. We also examined the topographical and source space distribution of these intervals. Finally, we classified the paradigms into specific clusters of experimental designs, an approach that will guide future research and inspire the development of novel tasks to study value-based decisions. Overall, our findings show that there are similarities as well as differences in the time course and the topography of EDM and IDM processes. To our knowledge, the current review is the first to directly contrast these two types of decision-making and to provide an in-depth description of the paradigms and ERP/ERF components that are most consistently reported in the field.

## 77. SANDIAMICO: An Open-Source Toolbox for Soma and Neurite Density Imaging (SANDI) with AMICO

Mario Ocampo-Pineda ^1^, Simona Schiavi ^2^, Raphaël Truffet ^3^, Emmanuel Caruyer ^3^, Alessandro Daducci ^4^, Marco Palombo ^5^

^1^  University Hospital Basel and University of Basel, Translational Imaging in Neurology (ThINK) Basel, Department of Biomedical Engineering, Faculty of Medicine, Basel, Switzerland

^2^  University of Genoa, Department of Neuroscience, Rehabilitation, Ophthalmology, Genetics, Maternal and Child Health (DINOGMI), Genoa, Italy 

^3^  Univ Rennes, CNRS, Inria, Inserm, Rennes, France 

^4^  University of Verona, Department of Computer Science, Verona, Italy 

^5^  Cardiff University, Cardiff University Brain Research Imaging Centre (CUBRIC), School of Psychology and School of Computer Science and Informatics, Cardiff, UK

The soma and neurite density imaging (SANDI) model has been recently proposed to estimate magnetic resonance imaging (MRI) indices of apparent neurite and soma density noninvasively in the brain. The SANDI model assumes that soma (neuronal and glial cell bodies) and neurites (axons, dendrites, and glial processes) can be approximated as two non-exchanging compartments, modeled as spheres of certain size and cylinders of zero radius (“sticks”), respectively. However, the estimation of these parameters from diffusion-weighted magnetic resonance imaging (dMRI) measurements is challenging and time consuming when using conventional fitting methods. To overcome this issue, we propose SANDI_AMICO: a new implementation of SANDI inside the Accelerated Microstructure Imaging via Convex Optimization (AMICO) framework.

SANDI_AMICO rewrites the SANDI model as a linear system Ax = y, where A is a matrix containing simulated signals (i.e., response functions) of each compartment, y is the vector of measured signals, and x is the vector of unknown contributions. In general, how we build matrix A impacts the results of the estimated model parameters. Here, we propose a data-driven approach for the design of an optimal matrix A, given a dMRI acquisition and the SANDI model.

We used both analytical simulations and in vivo human data to compare the performance of AMICO with and without matrix A optimization under controlled and real conditions, respectively. On synthetic data, SANDI_AMICO showed higher robustness to noise, and it was over 30x faster than a conventional fitting method. On in vivo data, we observed that the variations of the apparent soma density over the midpoint cortical surface followed the expected cyto-architectonics of several Broadmann’s areas. We showed that the SANDI_AMICO implementation provides a fast and robust estimation of SANDI parameters furthering their use in several neuroscience applications.

## 78. Microglial Large Extracellular Vesicles Propagate Early Synaptic Dysfunction in Alzheimer’s Disease by Moving at the Axon Surface

Martina Gabrielli ^1^, Ilaria Prada ^1^, Elisa Tonoli ^2^, Chiara Falcicchia ^3^, Giulia D’Arrigo ^1^, Grazia Rutigliano ^4^, Elisabetta Battocchio ^5^, Rossella Zenatelli ^6^, Francesca Tozzi ^7^, Annalisa Radeghieri ^6^, Ottavio Arancio ^8^, Elisabetta Verderio-Edwards ^2^, Nicola Origlia ^3^, Claudia Verderio ^1^

^1^  CNR Institute of Neuroscience, Dept. of Biomedical Sciences, Vedano al Lambro, Italy 

^2^  Nottingham Trent University, School of Science and Technology, Nottingham, UK 

^3^  CNR Institute of Neuroscience, Dept. of Biomedical Sciences, Pisa, Italy 

^4^  CNR Institute of Clinical Physiology, Dept. of Biomedical Sciences, Pisa, Italy 

^5^  University of Milano-Bicocca, School of Medicine and Surgery, Monza, Italy 

^6^  University of Brescia, Dept. of Molecular and Translational Medicine, Brescia, Italy 

^7^  Scuola Normale Superiore, Bio@SNS laboratory, Pisa, Italy 

^8^  Columbia University, The Taub Institute for Research on Alzheimer’s Disease and the Aging Brain, New York, NY, USA

Synaptic dysfunction occurs early in Alzheimer’s disease (AD), involving progressively larger areas of the brain over time. However, how it starts and propagates is unknown. We show that amyloid-beta (Aβ) released by microglia in association with large extracellular vesicles (Aβ-EVs) alters dendritic spine morphology in vitro and impairs synaptic plasticity both in vitro and in vivo in the entorhinal cortex-dentate gyrus circuitry. 1h after Aβ-EV injection into the mouse entorhinal cortex (EC), long-term potentiation (LTP) is impaired in the EC, while 24 h later it is impaired also in the dentate gyrus (DG), revealing a spreading of LTP deficit between the two synaptically connected regions. Similar results are obtained by injecting EVs carrying Aβ naturally secreted by CHO7PA2 cells, while neither Aβ42 alone nor inflammatory EVs devoid of Aβ are able to propagate LTP impairment. Using optical tweezers coupled to time-lapse imaging to study Aβ-EV-neuron interaction, we show that Aβ-EVs move anterogradely at the axon surface and that their motion can be blocked by annexin-V coating. Importantly, when Aβ-EV motility is inhibited, no propagation of LTP deficit occurs along the EC-DG circuit, implicating large EV motion at the neuron surface in the spreading of LTP impairment. Accordingly, proteomic analysis displays differences in the composition of Aβ-EVs vs. EVs from microglia not exposed to Aβ. The influence of mesenchymal stem cell (MSC) indirect co-colture with microglia primed with Aβ on cell phenotype, EVs and functions is currently being explored. Our data indicate the involvement of large microglial EVs in the rise and propagation of early synaptic dysfunction in AD, and suggest a new mechanism controlling the diffusion of large EVs and their pathogenic signals in brain parenchyma, paving the way for novel therapeutic strategies to delay the disease. Fundings: Horizon2020#874721PREMSTEM, 2018-AARF-588984.

## 79. The Synergistic Role of SMN and eIF3e in Ribosome Heterogeneity and the Impact of Their Loss in Spinal Muscular Atrophy

Deborah Donzel ^1^, Toma Tebaldi ^2^, Ilaria Signoria ^3^, Yu-Ting Huang ^4^, Ilaria Bruno ^1^, Nora Detering ^5^, Ewout J.N. Groen ^3^, Dinja Van der Hoorn ^4^, Marta Marchioretto ^1^, Gaurav Sharma ^1^, Heidi Fuller ^6^, Giulia Maule ^2^, Alessandro Umbach ^2^, Anna Cereseto ^2^, Alessandro Quattrone ^2^, Ludo van der Pol ^3^, Alberto Inga ^2^, Peter Claus ^5^, Marina Boido ^7^, Thomas H. Gillingwater ^4^, Gabriella Viero ^1^

^1^  CNR, IBF, Trento, Italy 

^2^  University of Trento, CIBIO, Trento, Italy 

^3^  Utrecht Brain Center, Department of Neurology and Neurosurgery, Utrecht, The Netherlands 

^4^  University of Edinburgh, Edinburgh Medical School: Biomedical Sciences & Euan MacDonald Centre for Motor Neuron Disease Research, Edimburgo, UK 

^5^  SMATHERIA gGmbH, Non-Profit Biomedical Research Institute, Hannover, Germany 

^6^  University of Keele, Wolfson Centre for Inherited Neuromuscular Disease, Keele, UK 

^7^  University of Turin, “Neuroscience Institute Cavalieri Ottolenghi, Torino, Italy

Spinal muscular atrophy (SMA) is a neurodegenerative disorder caused by decreased expression of survival motor neuron (SMN) protein. The mechanism through which the disease develops is unknown yet. Recently, it has been demonstrated that SMN protein is a Ribosome Associated Factor (RAF), and that SMN-primed ribosomes modulate the translation efficiency of a specific subset of transcripts related to the disorder. To understand the extend of SMN-primed ribosome heterogeneity, we obtained the entire catalogue of protein interactors of SMN-primed ribosomes. By proteomics analysis of SMN-primed ribosomes obtained from control mouse tissues, we identified hundreds of RNA-independent interactors. This finding supports the hypothesis that ribosomes are much more complex molecular machines than previously thought. To identify putative direct SMN interactors on ribosomes, we intersected our proteomics analysis with the SMN proximal interactome and found several proteins belonging to the eIF3 complex. In addition to its classical role in translation initiation, eIF3 has been recently described as a RAF involved in muscle physiology. By integrating SMN- and eIF3e-specific multi-omics data, we observed a robust and significant overlap between down-regulated transcripts upon SMN loss and eIF3 silencing, and RNAs associated with SMN-specific and eIF3-specific ribosomes. Intriguingly, both SMN and eIF3e-specific ribosomes display a preference for binding mRNAs with IRES-like sequences. Next, we confirmed the association of SMN and eIF3e to ribosomes and ribosomal subunits in multiple control tissues and the loss of their association in SMA or in eIF3e KO-cell lines, suggesting that decreased levels of SMN lead to a corresponding alteration in the co-sedimentation profile of eIF3e with ribosomes. These results highlight the existence of a functional interplay between SMN and eIF3e on ribosomes, strengthening our hypothesis that SMN plays a crucial role in translation regulation.

## 80. A preliminary In Vitro Study to Assess the Stressor Effect on Amyotrophic Lateral Sclerosis Onset and Progression

Daniela Maria Rasà ^1^, Ilaria Stoppa ^2^, Marina Boido ^1^

^1^  Neuroscience Institute Cavalieri Ottolenghi, Department of Neuroscience Rita Levi Montalcini, University of Turin, Turin, Italy 

^2^  Neuroscience Institute Cavalieri Ottolenghi, University of Turin, Turin, Italy

Nowadays, worldwide people are continuously exposed to many stressors, due to exhausting lifestyle, increased population density, pollutants and environmental/global changes. These conditions can trigger several cellular alterations, in turn predisposing to neurodegenerative diseases, as amyotrophic lateral sclerosis (ALS). ALS is a motor neuron (MN) disease, determining weakness, muscle atrophy and premature death. It is characterized by excitotoxicity, oxidative stress, and neuroinflammation, with cellular processes activated also by stressor exposure. This study aims to clarify the contribution of different stressors in causing/anticipating ALS, since many mechanisms are still unclear. Preliminary experiments have been set-up in vitro, using NSC-34 cells expressing hSOD1(G93A) gene under the control of a doxycycline-inducible promoter. To differentiate the cells in MN-like cells, different retinoic acid (RA) concentrations have been tested: RA (1, 5, 10, 15 or 20 µM) was added to the culture medium for two, four, six, and eight days. Based on the MTT assay results, 20 µM RA for four days represents the most proper condition to induce cell maturation. Concerning the overexpression of hSOD1(G93A), the cells were grown in complete medium and 5 µg/mL of doxycycline for 24 h, confirming hSOD1 expression by WB, both in undifferentiated and differentiated cells. Finally, to mimic a stress condition, cells underwent oxygen glucose deprivation: CoCl2 was used as hypoxic agent and its toxicity was measured by MTT assay: different concentrations of CoCl2 were evaluated in both high and low glucose medium, suggesting 100 µM CoCl2 in low glucose as optimal stress condition. With these preliminary experiments, we have set-up the conditions for the next analyses, to evaluate genetic/epigenetic mutations and cellular/molecular alterations, and to clarify the stressor impact on the neurons and on the predisposition to neurological pathologies.

## 81. Multifunctional Liposomes Increase Synaptic Transmission Strength in Mouse Cortical Neurons

Giulia Terribile ^1^, Sara Di Girolamo ^1^, Paolo Spaiardi ^2^, Gerardo Biella ^2^, Silvia Sesana ^3^, Francesca Re ^3^, Giulio Sancini ^3^

^1^  University of Milano-Bicocca, School of Medicine and Surgery, Monza, Italy 

^2^  University of Pavia, Department of Biology and Biotechnologies, Pavia, Italy 

^3^  University of Milano-Bicocca, School of Medicine and Surgery, Nanomedicine Center, Monza, Italy

Alzheimer’s disease (AD) is characterized by the accumulation of plaques of β-amyloid (Aβ) peptide in the brain. Given its pivotal role, new nanotechnological therapeutic tools targeting Aβ are in demand. For this purpose, we synthesized bifunctional liposomes (mApoE-PA-LIP) with a peptide derived from the apolipoprotein-E receptor-binding domain (mApoE) for blood–brain barrier (BBB) targeting and with phosphatidic acid (PA) for Aβ binding. Our previous results indicate that mApoE-PA-LIP can destabilize brain Aβ aggregates and promote peptide removal across the BBB and its peripheral clearance both in vitro and in vivo models with memory improvement. We firstly evaluated the impact of mApoE-PA-LIP on intracellular Ca2+ dynamics in hCMEC/D3, as an in vitro human BBB model, and in cultured astrocytes, that are both among the main components of the neurovascular unit. Our results show that the mApoE-PA-LIP pre-treatment increases the ATP-evoked intracellular Ca2+ waves, both in the presence and absence of extracellular Ca2+, indicating that this effect is mainly due to endogenous Ca2+ release from endoplasmic reticulum (ER). Indeed, blocking Sarco-ER Ca2+ ATPase (SERCA) activity with cyclopiazonic acid, ATP failed to trigger any intracellular Ca2+ waves, indicating that metabotropic purinergic receptors (P2Y) are mainly involved. So, we assessed whether mApoE-PA-LIP can modulate the neuronal synaptic transmission in the cortical area. For this purpose, we performed electrophysiological recordings, using the whole-cell patch-clamp technique, on mouse brain slices. We evaluated inward and outward total currents, firing pattern, spontaneous excitatory post-synaptic currents (sEPSCs) frequency, and amplitude before and after the perfusion with mApoE-PA-LIP. Our preliminary data suggest that mApoE-PA-LIP trigger an increase of sEPSCs frequency. The results here outlined could give additional support to promote mApoE-PA-LIP as a putative therapeutic tool for AD treatment.

## 82. Co-Ultramicronized Palmitoylethanolamide/Luteolin Prevents Alteration in Astrocyte-Oligodendrocyte Crosstalk Relevant for Myelination in an In Vitro Model of β-Amyloid Toxicity

Marta Valenza, Roberta Facchinetti and Caterina Scuderi

Sapienza University of Rome, Dept. Physiology and Pharmacology Vittorio Erspamer, Rome, Italy

Astrocytes are cells pivotal for the correct functioning of the brain. One of their less explored functions concerns their ability to affect myelination by supporting oligodendrocytes precursor cells (OPCs). Brain imaging studies link myelin impairments with Alzheimer’s disease (AD), appointing beta-amyloid (Aβ) deposition as a possible etiological factor. However, recent evidence reported that Aβ stimulates OPCs maturation. To clarify this counterintuitive evidence, we studied the interaction between astrocytes and oligodendrocytes, with particular emphasis on astrocyte reactivity and the release of trophic factors required for OPCs maturation, by setting up an in vitro model of Aβ1-42 toxicity. In a transwell system we posed in co-culture rat primary astrocytes and OPCs. We treated astrocytes with human Aβ1-42 and analyzed what astrocytic alterations relevant to myelination occurred, as well as how that challenge affected the maturation of co-cultured OPCs. As a possible treatment to dampen Aβ1-42 effects, we tested a formulation of palmitoylethanolamide combined with the flavonoid luteolin (co-ultra PEALut), that we previously demonstrated to be able to counteract Aβ-induced alteration.

Astrocytes exposed to human Aβ1-42 increased the transcription of pro-inflammatory cytokines and reduced that of specific factors implicated in OPCs maturations. We also observed severe morphological changes in co-cultured OPCs, which lose the complexity of arborization, indicating aberrant maturation. Co-ultra PEALut prevented such pathological changes and some of these effects were mediated by the peroxisome proliferator-activated receptor alfa.

Considering that co-ultra PEALut is already approved for human use as a dietary supplement, altogether, our findings open new opportunities for the treatment of diseases characterized by myelination impairments such as AD.

## 83. Mitochondrial SMN1-Anticorrelated Genes as Potential Targets for Spinal Muscular Atrophy Therapy

Anna Caretto, Ferdinando Di Cunto, Marina Boido and Alessandro Vercelli

University of Turin, Neuroscience Institute Cavalieri Ottolenghi, Department of Neurosciences “Rita Levi Montalcini”, Orbassano (TO), Italy

Spinal muscular atrophy (SMA) is a pediatric disease caused by the mutation of survival motor neuron 1 (SMN1) gene and, consequently, low levels of SMN protein. It determines not only motor neuron (MN) loss in brainstem and spinal cord, but also the impairment of peripheral tissues (i.e., skeletal muscles and heart). Nowadays, the identification of new targets and therapeutic strategies is necessary to overcome some limitations of the SMN-dependent available therapies.

Recently it has been demonstrated that stress conditions can induce a morphofunctional “switch” in mitochondria that are considered the “powerhouse of the cells”. Since all the affected tissues in SMA require a lot of energy, mitochondria can be studied as promising targets for the investigation of new treatments.

In this context, through a bioinformatic approach, we searched for SMN1-anticorrelated mitochondrial genes whose expression could be normalized to regulate mitochondrial functionality. In particular, we identified some genes expressed in the most affected tissues in SMA (spinal cord, brain, skeletal muscles, and heart): GCSH, COX7A1, BAG1, GOLPH3, DNAJC5, SLC25A36, GLRX2, and UQCRC2. To assess their levels in SMA, we analyzed tissues obtained from SMNdelta7 mice (an intermediate model of SMA) after a period of behavioral testing, from postnatal day 2 (P2) to P5, sacrificing WT and SMA animals in an early symptomatic stage of the pathology (P5). Molecular analysis on collected samples revealed a marked upregulation of GCSH in SMA spinal cord, brain, and gastrocnemius, compared to controls.

Starting from this evidence, we can hypothesize an existing relationship between SMN1 and GCSH expression. Therefore, normalizing GCSH levels could lead to the restoration of mitochondrial integrity and to a recovery from cellular disease impairments.

## 84. The chrOMICles of ALS Spinal Cord Organoids, OMIC Characterization of Patient-Derived Spinal Cord Organoids to Unravel New Therapeutic Targets in C9ORF72 form of Amyotrophic Lateral Sclerosis

Noemi Galli ^1^, Mafalda Rizzuti ^2^, Lorenzo Brambilla ^2^, Jessica Ongaro ^2^, Chiara Cordiglieri ^3^, Monica Nizzardo ^1^ and Stefania Corti ^1^

^1^  Università degli Studi di Milano, Dipartimento di Fisiopatologia Medico-chirurgica e dei Trapianti, Università degli Studi di Milano, Milano, Italy 

^2^  Fondazione IRCCS Ca’ Granda Ospedale Maggiore Policlinico (Milano), UOC Neurologia, Milano, Italy 

^3^  Istituto Nazionale di Genetica Molecolare (INGM), IRCCS Ca’ Granda Ospedale Maggiore Policlinico, Milano, Italy

Amyotrophic lateral sclerosis (ALS) is a rare neurodegenerative disorder involving motor neurons (MNs) in brain and spinal cord, resulting in progressive muscle atrophy and weakness that eventually compromise diaphragm functionality. No efficacious treatment is currently able to halt or reverse disease progression, making it mandatory to develop novel therapeutic approaches that would improve the lives of the patients affected by this devastating disease. In the study of pathophysiology, 3D models are a promising powerful tool that can recapitulate the complex architecture of tissues in a more accurate manner than 2D cultures. The objectives of this work included the characterization of spinal cord organoids to refine the reliability and reproducibility of the differentiation protocol, as well as to delineate the ALS phenotype with omic techniques; over and above, the selection of ALS-related candidates from the outlined transcriptomic and proteomic profiles. Our spinal cord-like organoids displayed neural progenitors that progressively decreased post-mitotic neurons, MNs, and glia. Organoids were collected at 30, 55, and 80 days in vitro (DIV) and evaluated for their morphology and neurodevelopmental features by IHC and qPCR. Specifically, DIV80-organoids expressed SMI32, TUBB3, MAP2, DCX, OLIG2, PAX6, HOXB4, GFAP, and S100β. Besides astrogliosis, the C9 condition interestingly showed PRPH aggregation, as described in literature. Mass spectrometry and gene ontology depicted an enrichment in pathways related with cytoskeletal coordination, synaptic functionality, astrocyte reactivity, and stress response in C9-ALS condition. Single-cell RNA sequencing and gene annotation disclosed the predominance of neuroectoderm and neural cell populations in the samples, remarking the potential of this disease model. Overall, this project might allow the assessment of novel candidate genes linked with C9ORF72-ALS pathogenesis and their potential as therapeutic targets.

## 85. Cognitive Frailty and Oxygen-Ozone Therapy: Differential Expressed Genes as Predictive Biological Markers of Response/Improvement to Treatment

Chiara D’Amelio ^1^, Valeria Manzini ^2^, Cristian Bonvicini ^3^, Catia Scassellati ^4^, Ivan Arisi ^5^, Rossella Brandi ^6^, Mara D’Onofrio ^2^, Antonino Cattaneo ^7^

^1^  European Brain Research Institute (EBRI) “Rita Levi-Montalcini”, Genomics Facility, Rome, Italy 

^2^  European Brain Research Institute (EBRI), Genomics Facility, Rome, Italy 

^3^  IRCCS Istituto Centro San Giovanni di Dio Fatebenefratelli, Molecular Markers Laboratory, Brescia, Italy 

^4^  IRCCS Istituto Centro San Giovanni di Dio Fatebenefratelli, Biological Psychiatry Unit, Brescia, Italy 

^5^  European Brain Research Institute (EBRI), Bioinformatic Facility, Rome, Italy 

^6^  Army Medical Center, Scientific Department, Rome, Italy 

^7^  European Brain Research Institute (EBRI), Rome, Italy

Frailty is a multidimensional geriatric syndrome characterized by increased vulnerability to stressors as a result of the reduced functional capacity of different physiological systems. This heterogeneous clinical syndrome is known to show not only a physical or biological dimension, but also a multidimensional concept, that includes cognitive frailty (CF). Biological mechanisms and specific treatments for CF are still understudied. Oxygen-Ozone (O2-O3) therapy is a non-invasive/non-pharmacological low-cost procedure with no side effects based on therapeutic effects of low O3 concentrations that can induce a mild oxidative stress stimulating antioxidant defenses, and preventing the inflammatory response and cell damage. We hypothesized that O2-O3 therapy might induce a significant effect on those oxidative and inflammation processes, strongly altered in CF. We thus conducted the first pilot double blind randomized controlled trial where the group of elderly frail subjects aged between 65 and 80 were stratified as untreated, treated with O2 and O2-O3 mixture. The methodology consisted in rectal insufflations for five weeks (three sessions for week), and a total amount of 150cc of O2-O3 mixture at the concentration of 30 µg of O3 per cc of O2 over a 5–10 min period was administered. The mRNA profiling was analyzed in whole blood from 72 CF subjects by Agilent microarray, and measured before (T0) and three months post-treatment (T1). Preliminary results highlighted a specific mRNA response to treatment, which involves differentially expressed mRNAs of several pathways. These biomarkers will be integrated with clinical and neuropsychological data, allowing to predict and optimize the therapeutic response to O2-O3 therapy.

## 86. The Neuropathology of the SARS-CoV-2: An Autoptic COVID-19 “Biobanking” of Brain Specimens for Future Translational Biomedical Research

Stigliano Egidio ^1^, Amadoro Giuseppina ^1,2^, Dell’Aquila Marco ^1^, Concetta Cafiero ^1^, Ugo Di Tondo ^1^, Maria Luisa Lavitrano ^3^, Guido Rindi ^1^, Arena Vincenzo ^1^

^1^  Catholic University of Sacred Heart, Largo Francesco Vito, 00168 Rome, Italy

^2^  National Research Council, Institute of Translational Pharmacology (IFT), Via Fosso del Cavaliere, 100, 00133 Rome, Italy

^3^  University Milano Bicocca, Department of Medicine and Surgery & Director BBMRI.it, Milan, Italy

Neurological symptoms are detected in Severe Acute Respiratory Syndrome Coronavirus 2 (SARS-CoV-2) infected individuals and biomarkers of viral involvement of the nervous system in individuals who died of coronavirus disease 2019 (COVID-19) related respiratory failure are documented (NEUROCOVID). Autopsy is the “gold standard” for understanding the etiopathogenetic mechanisms associated with the onset of morbid processes, including the COVID-19 pandemic. From 2019 up to now, we performed 500 autopsies, including 200 on deceased patients with diagnosis of SARS-CoV-2 related pneumonia to provide clinicians with appropriate feedback. Approximately 27% of cases (range 74–93 years) were hospitalized patients affected by neurodegenerative processes such as Alzheimer’s disease, senile dementia and Lewy body dementia. The diagnosis of SARS-CoV-2 infection was initially carried out by RT-PCR on oropharyngeal swabs, then confirmed on post-mortem lung tissues. We performed a trasnethmoidal approach to the skull using Jamshidi needle, in order to obtain a “window” to the brain and to reduce the risk of exposition to the virus for the autopsy staff. A minimally invasive autopsy technique was also executed for body sampling. We collected specimens of all tissues that were formalin-fixed and paraffin embedded while brain and olphactory bulbs and CSF were stored at −80 °C. Histological (H/E) and molecular (RT-PCR) investigations revealed the presence of SARS-CoV-2 in all organs, tissues, cells and biological fluids. The SARS-CoV-2 presence was documented especially in lung parenchyma and upper respiratory tract secretions with concordance with clinical data greater than 95%. We propose that the large number of clinical data and biological samples can represent a valuable collection for future BBMRI.it COVID-19 “biobanking” activities and for translational research aimed at understanding the pathogenetic mechanism underlying the SARS-CoV2 infection and developing effective therapies.

## 87. Transcriptome-Phenotype Relationship in Unmutated Sporadic ALS Patients Highlights Phenotype-Specific Gene Expression Patterns

Maria Garofalo ^1^, Loris Grieco ^2^, Maria Busacca ^2^, Matteo Bordoni ^3^, Francesca Dragoni ^2^, Jessica Garau ^4^, Eveljn Scarian ^5^, Rosalinda Di Gerlando ^1^, Giuseppe Fiamingo ^5^, Luca Diamanti ^6^, Orietta Pansarasa ^3^, Stella Gagliardi ^1^

^1^  IRCCS Mondino Foundation, Molecular Biology and Transcriptomics Unit, Pavia, Italy 

^2^  University of Pavia, Department of Biology and Biotechnology “L. Spallanzani”, Pavia, Italy 

^3^  IRCCS Mondino Foundation, Cellular Models and Neuroepigenetics Unit, Pavia, Italy 

^4^  IRCCS Mondino Foundation, Neurogenetics Research Centre, Pavia, Italy 

^5^  University of Pavia, Department of Brain and Behavioral Sciences, Pavia, Italy 

^6^  IRCCS Mondino Foundation, Neuro-Oncology Unit, Pavia, Italy 

Amyotrophic lateral sclerosis (ALS) is a progressive and fatal neurodegenerative disorder affecting human motor system, characterized by heterogeneity and phenotypes variability. This background highlights a critical need for identification of an adequate strategy to stratify ALS patients and to identify reliable biomarkers for early diagnosis, prognosis, and disease progression. The discovery of the involvement of RNA-mediated toxicity, which could be controlled by the alteration of gene expression, is considered a key event in ALS. Regulation of gene expression represents a novel opportunity to identify specific traits in ALS subgroups. For these considerations, the classification based on specific clinical phenotypes could be associated with different gene expression patterns that are shaped during lifespan, representing a novel opportunity to identify specific ALS subtypes with homogeneous clinical and biological features.

Our objective is to identify the transcriptomic signatures of distinct ALS phenotypes, and to use this information for biomarker assessment and personal therapy development.

We characterized 36 unmutated sALS patients by clinical and paraclinical phenotype and subdivided them in “Classic” (n = 12), “Bulbar” (n = 7), “Flail Arm” (n = 6), “Flail Leg” (n = 6) and “Pyramidal” (n = 5). Then, RNAs extracted from PBMCs isolated from patients (n = 15) and healthy controls were sequenced. By performing a Principal Component Analysis (PCA) of DE genes in each group of patients compared to controls, we observed a gene expression clusterization of patients and controls, except for “Flail Arm” group. Interestingly, we found only one gene commonly deregulated in all groups (Y RNA, a component of the Ro60 ribonucleoprotein), while the remaining DE genes were phenotype-specific.

The identification of phenotype-specific pathogenic mechanisms will be crucial for the prognosis and the identification of new therapeutic targets to delay onset or attenuate disease progression rate.

## 88. The Possible Role of Cholesterol Metabolism in the Onset and Progression of Huntington’s Disease

Monica Favagrossa ^1^, Alice Passoni ^2^, Helena Villavicencio ^1^, Alessia Lanno ^2^, Rebecca Camastra ^1^, Giulia Birolini ^3^, Marta Valenza ^3^, Mario Fichera ^4^, Elena Cattaneo ^3^, Caterina Mariotti ^4^, Renzo Bagnati ^2^, Laura Colombo ^1^, Mario Salmona ^1^

^1^  Istituto di Ricerche Farmacologiche Mario Negri IRCCS, Biochimica e Farmacologia Molecolare, Milano, Italy 

^2^  Istituto di Ricerche Farmacologiche Mario Negri IRCCS, Ambiente e Salute, Milano, Italy 

^3^  Università degli studi di Milano, Milano, Italy 

^4^  Istituto Neurologico Carlo Besta, Milano, Italy

Huntington’s disease (HD) is an autosomal dominant neurodegenerative disorder caused by the abnormal expansion of the CAG repeats in the first exon of the IT15 gene, which encodes for an expanded PolyQ tract in the huntingtin protein (HTT). One of the affected pathways is cholesterol (Chol) metabolism. Many data in vivo and in vitro have shown that the presence of mHTT causes a reduction in cholesterol synthesis and an alteration of its turnover. 24OH-cholesterol (24OHC) is the main metabolite of Chol in the brain. Unlike Chol, it can cross the blood–brain barrier and enter peripheral blood circulation, and 24OHC can be considered an indirect indicator of Chol metabolism in the brain. A biomarker is needed to monitor disease progression. This project aimed to characterize the cholesterol metabolism in an HD mouse model using an LC-MS method and evaluate 24OHC as a biomarker of disease progression in a clinical trial. The HD mouse model efficiently represents the disease progression at a behavioral level. In the striatum, the results show a significant difference between WT and HD animals at 12 weeks in desmosterol and 24OHC levels when the worst behavioral defects were found in the animals. There is no difference between 24OHC levels between HD and WT mice in plasma. In parallel, a clinical trial is ongoing to study plasmatic 24OHC in HD patients to evaluate the metabolite as a biomarker of HD progression.

These preliminary data suggest that a deficit of cholesterol metabolism correlates with the worst HD phenotype in animals and suggests that a decrease of desmosterol and 24OHC production occurs only at 12 weeks of age when HD symptoms are very severe in HD animals. Still, it will be necessary to use a mouse model with slower disease progression to deepen our understanding of the deficit in cholesterol synthesis.

## 89. Mitochondrial Alterations in Subjects with Idiopathic REM Sleep Disorders as a Predictive Biomarker for Conversion to Parkinson’s Disease

Gerardo Ongari ^1^, Silvia Cerri ^1^, Micol Avenali ^1^, Michele Terzaghi ^1^ and Fabio Blandini ^2^

^1^  IRCCS Fondazione Mondino, Unità di Neurobiologia Cellulare e Molecolare, Pavia, Italy 

^2^  University of Pavia, Department of Brain and Behavioural Sciences, Pavia, Italy

One of the most important prodromal markers of Parkinson’s disease (PD) is the presence of idiopathic rapid eye movement (REM) sleep disorders (iRBD). Nonetheless, only a few studies have looked into the mechanisms that may be involved in the development of PD in iRBD patients. Since mitochondrial dysfunction has been linked to sleep disturbances in PD, we investigated potential mitochondrial alterations in fibroblasts of subjects with iRBD in order to identify a biochemical profile that can characterize this condition and predict the future onset of PD.

The project included 23 subjects so far, divided into three experimental groups: healthy subjects (HC), iRBD subjects, and iRBD subjects subsequently converted to PD (iRBD-PD).

The evaluation of oxygen consumption rate (OCR) and extracellular acidification rate (ECAR) using a XFe24 Seahorse Analyzer, indices of the mitochondrial respiration efficiency, revealed a reduction in maximal and spare respiration levels in the fibroblasts of iRBD patients if compared to HC, though the difference was not statistically significant. Instead, a significant worsening of the bioenergetic profile was observed in the iRBD-PD patients, as evidenced by lower levels of adenosine triphosphate (ATP) production and reduced basal, maximal, and spare respiration. Moreover, the presence of mitochondrial fragmentation and a significant decrease in the expression levels of electron transport chain complexes III and V are associated with mitochondrial dysfunction in iRBD-PD patients. Similar, but less severe changes were observed in iRBD subjects.

These findings imply that mitochondrial alterations (e.g., a decreased ability to respond to increased energy demand) observed in iRBD subjects’ fibroblasts may predispose to worsening of the bioenergetic profile observed in iRBD subjects already converted to PD, indicating a potential mechanism underlying the progression of PD in iRBD patients.

## 90. Contingent Intramuscular Boosting of P2X7 Axis Improves Motor Function in Transgenic ALS Mice

Paola Fabbrizio ^1^, Jessica D’Agostino ^1^, Cassandra Margotta ^1^, Giulia Mella ^1^, Nicolò Panini ^2^, Laura Pasetto ^3^, Eliana Sammali ^3^, Flavia Raggi ^4^, Gianni Sorarù ^4^, Valentina Bonetto ^3^, Caterina Bendotti ^1^, Giovanni Nardo ^1^

^1^  Istituto di Ricerche Farmacologiche Mario Negri, Neuroscience, Milan, Italy 

^2^  Istituto di Ricerche Farmacologiche Mario Negri, Oncology, Milan, Italy 

^3^  Istituto di Ricerche Farmacologiche Mario Negri, Biochemistry and Molecular Pharmacology, Milan, Italy 

^4^  Azienda Ospedaliera di Padova, Neuroscience, Padua, Italy

Muscle weakness plays an important role in neuromuscular disorders comprising amyotrophic lateral sclerosis (ALS), a fatal neurodegenerative disorder that leads to progressive degeneration of motor neurons and severe muscle atrophy without effective treatment. Most research on the disease has been focused on studying motor neurons and supporting cells of the central nervous system. Strikingly, recent observations have shown that the expression of the SOD1G93A mutation in skeletal muscles causes denervation of the neuromuscular junctions, inability to regenerate and consequent atrophy, all clear symptoms of ALS, suggesting that these morpho-functional alterations in skeletal muscle precede motor neuron degeneration, bolstering the interest in studying muscle tissue as a potential target for the delivery of therapies.

We previously showed that the systemic administration of the P2XR7 agonist, 2′ (3′), O, (4, benzoylbenzoyl) adenosine 5, triphosphate (BzATP), enhanced the metabolism, improved the innervation and promoted the myogenesis of new fibres in the skeletal muscles of SOD1G93A mice. Here, we further corroborated this evidence showing that intramuscular administration of BzATP improved the motor performance of ALS mice by enhancing satellite cells and the muscle pro-regenerative activity of infiltrating macrophages. The preservation of the skeletal muscle retrogradely propagated along with the motor unit, suggesting that backward signaling from the muscle could impinge on motor neuron death. In addition to providing the basis for a suitable adjunct multisystem therapeutic approach in ALS, these data point out that the muscle should be at the centre of ALS research as a target tissue to address novel therapies in combination with those oriented to the CNS.

## 91. Investigation on the Neuroprotective Role That Astrocytes Exert on Neurons in the Context of Riboflavin Transporter Deficiency

Valentina Magliocca ^1^, Chiara Marioli ^2^, Enrico Silvio Bertini ^3^, Marco Tartaglia ^1^, Tiziana Persichini ^2^ and Claudia Compagnucci ^1^

^1^  Ospedale Pediatrico Bambino Gesù, Genetics and Rare Diseases Research Division, Rome, Italy 

^2^  Università degli Studi di Roma Tre, Scienze, Rome, Italy 

^3^  Ospedale Pediatrico Bambino Gesù, Dept. of Neuroscience, Unit of Neuromuscular and Neurodegenerative Diseases IRCCS, Rome, Italy

Riboflavin (Rf) is the precursor of flavin adenine dinucleotide (FAD) and flavin mononucleotide (FMN), two biological cofactors involved in different metabolic redox reactions, especially in mitochondrial processes. Mutations in the genes which encode for human riboflavin transporters hRFT2 and hRFT3 (called SLC52A3 and SLC52A2 respectively), cause a rare autosomal recessive disease that arises in childhood: riboflavin transporter deficiency (RTD). To better understand the patho-mechanisms which characterize this neurodegenerative disease, induced pluripotent stem cells (iPSCs) were used as in vitro cellular model. RTD is considered a motoneuronal progressive disease, as motoneurons (MNs) represent the most affected cell type. Since astrocytes have a neuroprotective role, we decided to investigate if iPSC-derived astrocytes (ASTROs) in co-culture with RTD MNs can improve their morphology and neuronal activity. To this aim, we differentiated RTD and Ctrl iPSCs into ASTROs and examined their effect on RTD MNs. The results obtained show that both RTD and Ctrl ASTROs are able to increase the neurites length and intracellular calcium (Ca^2+^) levels of RTD MNs. These results suggest that astrocytes may have a strong neuroprotection role on RTD neurons.

## 92. Disease Modifying Therapy Specifically Impacts on microRNAs Expression Profiling in Relapsing-Remitting Multiple Sclerosis

Valeria Manzini ^1^, Leonardo Malimpensa ^2^, Ivan Arisi ^1,3^, Rossella Brandi ^1,4^, Chiara D’Amelio ^1^, Francesca Malerba ^1^, Rita Florio ^1^, Sebastiano Crisafulli ^2,5^, Viola Baione ^2^, Gina Ferrazzano ^2^, Esterina Pascale ^6^, Marco Salvetti ^7^, Antonino Cattaneo ^1,8^, Antonella Conte ^2,9^, Mara D’Onofrio ^1,3^

^1^  European Brain Research Institute (EBRI) Rita Levi-Montalcini, Rome, Italy 

^2^  Department of Human Neurosciences, Sapienza University of Rome, Rome, Italy 

^3^  Institute of Translational Pharmacology, National Research Council (IFT-CNR), Rome, Italy 

^4^  Division of Molecular Biology, Immunology and Experimental Medicine. Scientific Department, Army Medical Center, Rome, Italy 

^5^  Neuroimmunology and Neuromuscular Diseases Unit, Fondazione I.R.C.C.S. Istituto Neurologico Carlo Besta, Milan, Italy 

^6^  Department of Medical-Surgical Sciences and Biotechnologies, Sapienza University of Rome, Rome, Italy 

^7^  Department of Neurosciences, Mental Health and Sensory Organs, Sapienza University, Rome, Italy 

^8^  Bio@SNS Laboratory of Biology, Scuola Normale Superiore, Pisa, Italy 

^9^  IRCCS Neuromed, Pozzilli, (IS), Italy

Multiple sclerosis (MS) is an autoimmune demyelinating and degenerative disease of the central nervous system characterized by heterogeneous clinical phenotypes, disease progression and response to disease-modifying therapies (DMTs). Diagnosis, monitoring and therapeutic choices are guided by neuroimaging and clinical neurological features that are considered to predict long-term disability. DMTs can change the disease course, especially for the relapsing remitting, RR-MS. Among DMTs, drugs depleting immune cells emerge for their efficacy: they can deplete specifically B or both B and T cells. However, the current clinical approach involves clinical and neuroimaging follow-up for at least one year before being able to define whether the drugs adequately control the disease. The unsatisfying availability of non-invasive and easily detectable molecular biomarkers represents an unmet need in clinical practice, for a more accurate diagnosis and better prognostic or response to treatment predictions. In this study, we focused on Cladribine and Ocrelizumab, which are widely used immune cell-depleting DMTs in Italian clinical MS Centres. Although the DMT mechanisms of action are relatively defined, their impact on gene expression is still unknown. Thus, we investigated the microRNAs profiling, proposed as diagnostic and prognostic tool for neuroinflammatory diseases, and their response to the Cladribine or Ocrelizumab treatment. The microRNA profiling was analyzed in peripheral blood mononuclear cells from 20 patients with RR-MS (Ethical approval Rif. 6361, Prot. 0635/2021), by Agilent microarray, and measured before (T0) and 6 months post-treatment (T1). Results highlight a specific microRNAs response to DMT, which involves differentially expressed microRNAs of the neuroinflammatory, immune-regulatory, and neurodegenerative pathways. These microRNA candidates, integrated with clinical and imaging data, might allow to predict and optimize the therapeutic response to DMT.

## 93. Stathmin-2 in Spinal Muscular Atrophy (SMA): Assessing Molecular and Therapeutic Role in SMA Human and Murine Models

Paolo Manzini, Elisa Pagliari, Michela Taiana, Giulia Ferrero, Manuela Garbellini, Giacomo Comi and Stefania Paola Corti

Fondazione IRCCS Ca Granda Ospedale Maggiore Policlinico di Milano, Unità di Neurologia, Milano, Italy

Spinal muscular atrophy (SMA) is a severe genetic neuromuscular disease with early onset and represents the most common genetic cause of infant mortality. SMA is caused by mutations in the survival motor neuron 1 gene (SMN1) that impair the function and survival of lower motor neurons (MNs) in the spinal cord. The majority of current SMA therapeutic approaches are focused on increasing the levels of full length SMN protein. However, finding SMN-independent approaches to target downstream pathological events can be valuable, particularly in the symptomatic phase of the disease.

To develop a complementary approach, one possibility is to identify downstream genes responsible for selective MN dysfunction. Stathmin-2 (STMN2), a gene involved in neurite outgrowth, cytoskeleton metabolism and axonal regeneration, was already observed to be a target in other neurodegenerative diseases as amyotrophic lateral sclerosis (ALS) and frontotemporal dementia (FTD). Therefore, we investigated the role of STMN2 in the context of SMA pathogenesis by studying its expression in SMA in vitro iPSC-derived MNs and in vivo murine models. Furthermore, we demonstrated that STMN2 overexpression, obtained using a lentiviral vector or a pharmacological drug, was able to increase survival, axon length, and neurite complexity in patients iPSC-derived MNs.

Overall, the investigation of the molecular and therapeutic role of STMN2 in SMA could offer new insights into increased MN vulnerability and may also support the finding of downstream modifier genes or SMN-independent therapeutic targets for a complementary SMA therapy that combines SMN-independent and SMN-dependent strategies.

## 94. Oxygen-Ozone Therapy and Cognitive Frailty: A Non-Pharmacological Approach to Potentially Resolve Immune and Inflammatory Dysfunctions

Miriam Ciani ^1^, Catia Scassellati ^2^, Roberta Zanardini ^1^, Antonio Carlo Galoforo ^3^, Ilaria Passeggia ^4^, Monica Almici ^4^, Cristina Geroldi ^5^, Cristian Bonvicini ^1^

^1^  IRCCS Istituto Centro San Giovanni di Dio Fatebenefratelli, Molecular Markers Laboratory, Brescia, Italy 

^2^  IRCCS Istituto Centro San Giovanni di Dio Fatebenefratelli, Biological Psychiatry Unit, Brescia, Italy 

^3^  Ozone Therapy Scientific Society (SIOOT); University of Pavia, Oxygen-Ozone Therapy Scientific Society (SIOOT), Gorle; University of Pavia, Pavia, Gorle and Pavia, Italy 

^4^  IRCCS Istituto Centro San Giovanni di Dio Fatebenefratelli, Laboratory of Alzheimer’s Neuroimaging and Epidemiology, Brescia, Italy 

^5^  IRCCS Istituto Centro San Giovanni di Dio Fatebenefratelli, Alzheimer Unit, Brescia, Italy 

Introduction. As the world’s population ages, cognitive frailty (CF) is becoming one of the most serious health problems and elucidating its biological mechanisms along with prevention and treatments becomes increasingly important also considering the associated health costs. We thus performed a clinical randomized trial where CF subjects received a non-pharmacological therapy based on the regenerative properties of ozone (O3) known to act on immune/inflammation processes, strongly altered in CF.

Methods. A cohort of 75 patients was stratified in non-, mildly- or severely frail rate and treated with placebo, oxygen (O2) or O2-O3. The serum levels of 27 peculiar pro- and anti-inflammatory cytokines and chemokine cell signalling molecules were measured by using the Bio-Plex Pro Human Cytokine 27-plex immunoassay. The student’s *t*-test and analysis of variance (ANOVA) followed by Tukey’s post hoc test were used for comparison of means between the groups.

Results. Preliminary analyses evidenced the implication, at different levels, of some molecules in relation to the frailty rate. Notably, we observed modulations of immune (i.e., inteleukin, IL-9) and inflammation (i.e., IL-1β) biomarkers at baseline (Time, T0) and after treatment (T1 = 3 months). Correlations between clinical CF profiles and peripheral levels of the considered biomarkers are ongoing to predict the response to O2-O3 therapy.

Conclusions. Although preliminary, these results confirm that the immune-inflammation systems are involved in the aetiopathogenetic mechanisms of CF, and that the related molecules could be potential therapeutic targets/biomarkers for the O2-O3 therapy. These data will further permit to validate a new non-pharmacological treatment approach for this condition.

## 95. A New Role of NBS1 in the Regulation of Primary Cilium

Mariaconcetta Augusto, Francesca Fabretti, Vittoria Nicolis di Robilant, Stefano Di Giulio, Veronica La Monica, Francesca Belardinilli, Damiana Battaglini, Marialaura Petroni and Giuseppe Giannini

Sapienza Università di Roma, Dipartimento di Medicina Molecolare, Roma, Italy

Microcephaly is caused by the depletion of neuronal progenitors and is usually associated with defects in proteins that localize at or regulate the centrosome. Notably, also defects in proteins of the DNA damage Response (DDR) can lead to microcephaly. Curiously, many DDR proteins localize and regulate centrosomal proteins, suggesting a functional link between centrosomes and DDR proteins that seems to converge in the control of the neuronal progenitor’s expansion. In interphase, the centrosome becomes the basal body (BB) and enucleates the primary cilium (PC), an organelle that is essential for the transduction of many mitogenic pathways also in neuronal progenitors. Indeed, ciliopathies, caused by defects in PC proteins, can be characterized by defects in brain development. We recently demonstrated that a DDR protein, NBS1, stably localizes at the centrosome/BB. Moreover, its depletion lengthens PC, affects the expression of PC-dependent pathways and perturbs the proliferation of neuronal progenitors, in vitro and in vivo. Because centrosome is known as regulator of PC assembly/disassembly and trafficking, we speculate that NBS1 could regulate PC working as an adapter at the BB. In order to address this issue, we propose to investigate the physical interactors of NBS1 at the BB. To this end, we will use an innovative method called “proximity-dependent biotin identification”. We will stably transfect RPE-1 cells with a plasmid expressing the sequence of NBS1 fused with a mutant of E.coli BirA biotin ligase. The cells will be incubated with an excess of biotin to allow the covalent biotinylation of protein near to NBS1. Then, we will purify the centrosome fraction performing mass spectrometry analysis on putative NBS1 interactors isolated by streptavidin beads. Our studies can better explain the mechanisms that regulate PC and they could give a possible justification for the neuronal phenotypes associated with defects in centrosome and DDR proteins.

## 96. In Vivo Functional Analysis of New Disease-Genes and Variants Impairing Trafficking and Cytoskeleton Dynamics as Underlying Cause of Undiagnosed Neurodevelopmental Diseases

Giulia Fasano ^1,†^, Martina Venditti ^1,†^, Graziamaria Paradisi ^2^, Catia Pedalino ^1^, Marco Tartaglia ^1^, Antonella Lauri ^1,^*

^1^  Bambino Gesù Children’s Hospital, IRCCS, Genetics and Rare Research Division, Rome, Italy 

^2^  Università della Tuscia, Department for Innovation in Biological, Agro-Food and Forest System, Viterbo, Italy

* Correspondence: antonella.lauri@opbg.net

† Equal contribution.

Rare diseases represent a serious societal burden, with at least 70% of the cases manifested already during childhood in chronic forms often affecting the nervous system and resulting in extremely debilitating conditions, which include early onset neurodegeneration. The lack of a fundamental understanding of the underlying pathophysiological mechanisms, which might involve a variety of cell populations and developmental processes, make them difficult to diagnose and treat. New and potentially pathogenic gene variants are continuously identified in undiagnosed patients thanks to advanced genomic technologies, resulting in an increased need for effective in vivo disease models to obtain functional validation. Here, we show the most recent examples which benefited from functional validation and in-depth analysis through ad hoc modeling and phenotyping in zebrafish obtained in our newly established facility. The in vivo workflow utilizes transient and stable (CRISPR-Cas9) gene knockdown and overexpression approaches for loss and gain of function conditions, coupled to improved minimally invasive genotyping and whole embryo imaging-based phenotype characterization at cellular and subcellular levels. We present zebrafish data which recently contributed to: (1) validating the pathogenicity of new disease genes and variants involved in endosomal trafficking, Golgi homeostasis, or autophagy and affecting neurodevelopment; (2) providing in-depth insights into the role and function of microtubules binding proteins in central nervous system and spinal neuron development and function; and (3) setting up pilot imaging-based in vivo readout systems for signaling involved in complex developmental syndromes.

## 97. Towards an In Vitro Model for Therapeutic Opportunities in Lafora Disease

Gabriele Trentini ^1^, Giulia Cazzanelli ^1^, Andrea Dalle Vedove ^1^, Marina Cardano ^1^, Alessandro Cutarelli ^1^, Nicolò Sbardellati ^1^, Giuseppe D’Orsi ^2^, Orazio Palumbo ^3^, Massimo Carella ^3^, Graziano Lolli ^1^

^1^  University of Trento, CIBIO, Trento, Italy 

^2^  Policlinico Riuniti, Centro per Lo Studio E la Cura Dell’Epilessia, Foggia, Italy 

^3^  Ospedale Casa Sollievo della Sofferenza, Genetica Medica, Foggia, Italy

Lafora disease (LD) is a rare, autosomal recessive, severe, and progressive myoclonus epilepsy. The onset occurs during adolescence with an average life expectancy of about ten years. Main symptoms include myoclonus and/or generalized seizures, visual hallucinations, and progressive neurological decline.

LD is mainly caused by loss-of-function mutations of the EPM2A gene (encoding laforin) or NHLRC1 gene (expressing malin). These proteins are involved in the metabolism of glycogen, ubiquitinating protein targeting to glycogen (PTG) which positively regulates the glycogen synthesis. In pathological conditions, PTG escapes from degradation due to dysfunctional laforin–malin complex, leading to the accumulation of structurally abnormal, insoluble glycogen into Lafora bodies, and driving the neurodegeneration.

Nowadays, neither a cure nor a relevant cellular model is available to allow a complete depiction of the pathophysiological mechanisms behind LD as well as the development and test of effective therapeutic strategies.

Therefore, we employed LD patient-derived induced pluripotent stem cells, reprogrammed from peripheral blood mononuclear cells. These have been characterized for the expression of pluripotency markers and their ability in generating the three germ layers. We managed to induce the neuralization into neural progenitor cells to further differentiate into pan-neurons, as an in vitro patient-derived neuronal model of LD. In parallel, we genetically modified a commercially available and stable human neural stem cell line (AF22) for the overexpression of PTG to boost the accumulation of glycogen.

On the other hand, we are performing structural studies on PTG crystals to test hit compounds able to bind the protein with the aim of inhibiting its activity.

Taken together, these models should allow the screening of drug hits emerging from the structure-based drug discovery approach and drug repurposing to select the best candidates for further pre-clinical trials.

## 98. A New Role of Nijmegen Breakage Syndrome Gene in Neuronal Development

Damiana Battaglini, Francesca Fabretti, Vittoria Nicolis di Robilant, Stefano Di Giulio, Veronica La Monica, Francesca Belardinilli, Mariaconcetta Augusto, Marialaura Petroni and Giuseppe Giannini

Università di Roma- La Sapienza, Medicina Molecolare, Roma, Italy

The DDR-defective syndromes are characterized by immune deficiency, cancer predisposition and neuronal defects (microcephaly and ataxia). One example is the Nijmegen breakage syndrome (NBS) caused by mutations in the NBS1 gene. Why DDR-defective syndromes patients show neuro-developmental defects has never been explained. We recently demonstrated that NBS1 stably localized at the centrosome/basal body and regulates primary cilia (PC) length and functionality. The PC is a non-motile organelle that works as an antenna for external stimuli and is essential for many mitogenic pathways. Defects in PC lead to diseases termed ‘ciliopathies’, some of which are characterized by defects in brain development and mental retardation (i.e., Joubert and Meckel syndromes). Therefore, we propose a model in which PC deregulation, due to NBS1 defects, is responsible for the neuronal defects observed in NBS patients. In order to address this issue, we need to verify whether hypomorphic mutations in the NBS1 gene affect PC structure and functionality. Consistent with this hypothesis, we found a significant increase in the PC length in human fibroblasts (HF) from NBS patients compared to the healthy ones. However, this is not a syngenic model. Therefore, in order to better evaluate our hypothesis in a syngenic context, we propose to generate RPE-1 cells in which we will both introduce the 675D5 mutation, the most frequently observed in NBS patients, in the NBS1 gene by Crispr/Cas9 technology and stably transfect a plasmid expressing a WT form of NBS1 under the control of an inducible promoter. In this cell model, we will analyze the localization of NBS1 and its interactors at the centrosome and we will study morphology, dynamic and functionality of the PC. Our study can represent an important step in understanding the molecular mechanism that causes neuronal defects in NBS and potentially in other DDR-defective syndromes and could reclassify NBS as a ciliopathy.

## 99. Expression of a Secretable, Cell-Penetrating CDKL5 Protein Enhances the Efficacy of AAV Vector-Mediated Gene Therapy for CDKL5 Deficiency Disorder

Manuela Loi ^1^, Giorgio Medici ^1^, Marianna Tassinari ^1^, Giuseppe Galvani ^1^, Stefano Bastianini ^1^, Laura Gennaccaro ^1^, Nicola Mottolese ^1^, Sara Alvente ^1^, Chiara Berteotti ^1^, Giovanna Zoccoli ^1^, Helen Rappe Baggett ^2^, Hiroyuki Nakai ^3^, Stefania Trazzi ^1^, Elisabetta Ciani ^1^

^1^  University of Bologna, Department of Biomedical and Neuromotor Science, Bologna, Italy 

^2^  Departments of Molecular and Medical Genetics and Molecular Immunology and Microbiology, Oregon Health & Science University, Portland, OR, USA 

^3^  Departments of Molecular and Medical Genetics and Molecular Immunology and Microbiology, Oregon Health & Science University, Portland, OR, USA

CDKL5 deficiency disorder (CDD) is a severe neurodevelopmental disease caused by mutations in the CDKL5 gene. CDD is characterized by early-onset epileptic seizures, hypotonia, intellectual disability, motor and visual impairment and respiratory dysregulation. There is currently no cure or effective treatment to ameliorate cognitive and behavioral symptoms for CDD. Although delivery of a wild-type copy of the mutated gene to cells represents the most curative approach for a monogenic disease, proof-of-concept studies highlight significant efficacy caveats for brain gene therapy. Herein, we develop a cross-correction-based strategy to enhance the efficiency of a gene therapy for CDD. We created a vector for gene therapy that produces an Igk-TATk-CDKL5 fusion protein that can be secreted via constitutive secretory pathways and, due to the transduction property of the TATk peptide, be internalized by neighboring cells. We carried out a comparative evaluation of CDKL5 and TATk-CDKL5 protein biodistribution in vivo and the effect of intravascular treatment with the AAVPHP.B_CDKL5 vector or AAVPHP.B_Ikg-TATk-CDKL5 vector on brain structure and behavior in adult symptomatic Cdkl5 knockout (KO) mice. We found that, although AAVPHP.B_Igk-TATk-CDKL5 and AAVPHP.B_CDKL5 vectors had similar brain infection efficiency, the first one led to a higher CDKL5 protein replacement due to secretion and transduction of the TATk-CDKL5 protein into the neighboring cells. Importantly, Cdkl5 KO mice treated with the AAVPHP.B_Igk-TATk-CDKL5 vector showed a behavioral and neuroanatomical improvement in comparison with vehicle-treated Cdkl5 KO mice or Cdkl5 KO mice treated with the AAVPHP.B_CDKL5 vector. These results indicate that a gene therapy based on a secretable recombinant TATk-CDKL5 protein is more effective at compensating Cdkl5-null brain defects than gene therapy based on the expression of the naive CDKL5.

## 100. Effects of Interleukin-9 on Striatal Synaptic Dysfunction in a Mouse Model of Multiple Sclerosis

Krizia Sanna ^1^, Alessandra Musella ^2,3^, Livia Guadalupi ^2^, Sara Balletta ^1^, Diego Centonze ^1,4^, Georgia Mandolesi ^2,3^

^1^  Dipartimento di Medicina dei Sistemi, Università Tor Vergata, Rome, Italy 

^2^  Synaptic Immunopathology Lab, IRCCS San Raffaele, Rome, Italy 

^3^  Department of Human Sciences and Quality of Life Promotion University of Rome San Raffaele, Italy 

^4^  IRCCS Istituto Neurologico Mediterraneo (INM) Neuromed, Pozzilli (IS), Italy

Multiple sclerosis (MS) is an autoimmune neuroinflammatory disease of the central nervous system (CNS) characterized by the infiltration of lymphocytes into the CNS resulting in a diffuse demyelination, neuroinflammation, neuroaxonal loss and dysfunction. Clinical and preclinical studies revealed that CNS inflammation drives a synaptic damage, named synaptopathy, independently of demyelination. Interleukin (IL)-9 is a cytokine that plays an important immunoregulatory role in MS and experimental autoimmune encephalomyelitis (EAE). However, the exact role of IL-9 in MS disease is still unclear.

Here, we studied the effect of IL-9 on clinical disability and striatal synaptic alteration in EAE mice. Our data clearly indicate beneficial effects of systemic IL-9 treatment in both presymptomatic and therapeutic stages in EAE mice. In particular, we observed that IL-9 treatment is able to induce a less severe disease course accompanied by a recovery of the spontaneous glutamatergic current kinetics in the striatum of EAE mice. Intracerebroventricular infusion (ICV) of IL-9 with osmotic minipumps implanted in the brain of EAE mice also ameliorates clinical disability and glutamatergic alteration suggesting a direct role of IL-9 into the CNS. The study of the receptor localization of IL-9 in the brain reveals a detectable expression of the IL-9 receptor on the microglia cell membrane, whereas no signal was observed from neurons or infiltrating lymphocytes, suggesting its indirect role on neuronal cells. Overall, these data suggest that IL-9 significantly ameliorates EAE pathogenesis by a direct effect into the CNS, likely due to modulation of resident immune cell activity.

## 101. The Role of SK Channels and the Vagus Nerve in Turning on AgRP Neurons in Experimental Autoimmune Encephalomyelitis

Eleonora Cornacchia ^^1^^, Maria Cristina Mariani ^1,2^, Erika Ricci ^1^, Kerlero de Rosbo Nicole ^1^, Antonio Uccelli ^1,2^, Tiziana Vigo ^1^

^1^  IRCCS San Martino Hospital, Neuroscience Lab, Genoa, Italy 

^2^  DINOGMI, University of Genoa, Genoa, Italy

Hypothalamic AGRP (agouti-related neuropeptide)-expressing neurons sense caloric needs to coordinate homeostatic feeding. Activation of AgRP neurons by fasting is mediated by N-methyl-D-aspartate receptors for glutamate and by the down-regulation of the inhibitory small-conductance calcium-activated potassium (SK) channels. Activation of AgRP neurons impairs hematopoiesis and increases the generation of regulatory T lymphocytes. Sensory fibers of the vagus nerve are involved in conveying glutamatergic signals from the periphery to the hypothalamus. AgRP neurons are activated upon induction of experimental autoimmune encephalomyelitis (EAE), and AgRP neuropeptide, produced by activated AgRP neurons, is increased in patients with multiple sclerosis (MS). We have analyzed, at different times upon immunization, the mRNA expression of AgRP, which increases when AgRP neurons are activated, in the hypothalamus of EAE-induced mice that underwent or not unilateral cervical vagotomy. We found that the increase in Agrp expression upon EAE induction is significantly less in mice that have undergone unilateral cervical vagotomy than in non-operated mice, indicating that the vagus nerve is implicated in AgRP neuron activation. Moreover, we observed that expression of AgRP is inversely correlated with the expression of Knnc1, the gene coding for SK1 channel, suggesting that modulation of SK1 channels is involved in activation of AgRP neurons in EAE. Our results support the possibility that glutaminergic signals transmitted by the vagus nerve and SK1 channels are involved in the modulation of AgRP neuron activity in EAE. Further experiments are necessary to establish whether electrical stimulation of the vagus nerve and/or pharmacological modulation of SK1 channels may be used to modulate the functionality of AgRP neurons and, thereby, hematopoiesis and lymphopoiesis in EAE.

## 102. Dendritic Cells Educated through Exposure to Specialized Pro-Resolving Mediators Acquire a Tolerogenic Phenotype

Marta Bottero ^1^, Giada Pessina ^2^, Fabrizio Loiacono ^3^, Nunzio Iraci ^4^, Loredana Leggio ^4^, Greta Paternò ^4^, Silvia Ravera ^5^, Nadia Bertola ^5^, Santina Bruzzone ^6^, Valerio Chiurchiù ^7^, Nicole Kerlero de Rosbo ^1^, Antonio Uccelli ^1^, Giovanni Ferrara ^1^

^1^  IRCCS Policlinico San Martino, Experimental Neuroscience, Genoa, Italy 

^2^  University of Genoa, Dinogmi, Genoa, Italy 

^3^  IRCCS Policlinico San Martino, Immunology, Genoa, Italy 

^4^  University of Catania, Biometec, Catania, Italy 

^5^  University of Genoa, Dimi, Genoa, Italy 

^6^  University of Genoa, Dimes, Genoa, Italy 

^7^  IRCCS Santa Lucia Foundation, Laboratory of Resolution of Neuroinflammation, European Center for Brain Research, Rome, Italy

In experimental autoimmune encephalomyelitis (EAE), the loss of immunological tolerance is one of the main autoimmune pathological mechanisms. In this context, dendritic cells (DCs) play a role in inducing both immunity and tolerance by acting as modulators of thymic and peripheral immune tolerance. We propose to generate tolerogenic DCs using a novel approach whereby DCs are exposed to specialized pro-resolving mediators (SPMs), a novel class of lipid autacoids that reduce tissue infiltration and activation of pro-inflammatory macrophages and T lymphocytes. Accordingly, we hypothesize that DCs conditioned by exposure to SPMs (DCsSPMs) could acquire a tolerogenic phenotype and could reduce the activation of T cells, thus ameliorating EAE. qPCR analysis showed that differentiation of bone-marrow-derived DCs induced to mature with LPS-INF in the presence of SPMs imparts a tolerogenic phenotype to these cells, with downregulation of pro-inflammatory markers (Cd40 and Il1b) and concomitant upregulation of tolerogenic markers Lilrb4, Cd274, and Pdcd1lg2. Moreover, mature DCsSPMs maintained the upregulation of those tolerogenic markers after overnight, 24 h and 48 h, migrated less upon SDF-1 and CCL19 engagement. Flow cytometry experiments confirmed that DCsSPMs upregulate the surface markers of tolerance, ILT3 and PD-L1, as well as other anti-inflammatory markers, such as MerTK and CTLA4. Activated T cells co-cultured with DCsSPMs or in the presence of supernatant of DCsSPMs, or their derived extracellular vesicles, produced low levels of pro-inflammatory cytokines INFγ and IL-17 and displayed a reduced mRNA expression of the transcription factors Tbx21 and Rorc, related to the inflammatory T-cell phenotypes. Furthermore, metabolic assessment of DCsSPMs revealed a complete coupling between ATP synthesis and oxygen consumption, suggesting that these DCs are anti-inflammatory. Our preliminary data suggest a novel role of SPMs in the induction of a tolerogenic phenotype of DCs.

## 103. Influence of the Sympathetic Nervous System on the Thymus: β3-Adrenergic Receptor-Expressing Stromal Cells as Sentinels of the Thymic Function

Maria Cristina Mariani ^1^, Tiziana Vigo ^2^, Erika Ricci ^2^, Eleonora Cornacchia ^2^, Nicole Kerlero de Rosbo ^2^, Antonio Uccelli ^2^

^1^  DINOGMI, University of Genoa, Genoa, Italy 

^2^  Experimental neuroscience lab, IRCCS Polyclinic Hospital San Martino, Genoa, Italy

The thymus is organized into discrete areas, in which the interaction of thymocyte precursors with stromal cells drive the maturation and the specification of T cells. The thymus is innervated by fibers of the sympathetic nervous system (SNS), that release norepinephrine (NE) in thymic environment. NE interacts with α and β2 adrenergic receptors (AR) expressed by thymocytes and with β3AR expressed by stromal cells. Hence, we have speculated that SNS signals acting on thymic stromal cells through β3AR, may promote T cell maturation and egress from the thymus. As NE increases in the thymus upon induction of experimental autoimmune encephalomyelitis (EAE), we have assessed the effect of the activation of β3AR on thymus homeostasis in EAE-induced mice treated with an antagonist of β3AR. To monitor T-cell maturation, we performed a FACS analysis of T lymphocytes within the thymus, and to evaluate the egress of mature lymphocytes from the thymus, we quantified TREC (T-cell Receptor Excision Circles) in the blood by Real-time PCR. We found that activation of β3AR increases the frequency of regulatory T (Treg) cells in thymus and increases the expression in stromal cells of IL15, a cytokine involved in the maturation process of Treg cells. The quantification of TREC in blood revealed an increase in newly generated T cells that move from the thymus into the blood upon activation of β3AR. As the decreased expression of sphingosine 1 phosphate receptor (S1P1) by T lymphocytes is the main mechanism involved in the retaining of mature T lymphocytes and in the expansion of Treg cells, we evaluated the expression of S1P1 and of Klf2, a transcription factor mediating the expression of S1P1; activation of b3ar induce a decrease expression of both molecules. Overall, our results indicate that the SNS controls the generation of Treg cells and the egress of newly generated T lymphocytes from the thymus through a mechanism that involves β3AR-expressing stromal cells.

## 104. Nutritional Overload Promotes Inflammatory Synaptic Damage and Disease Course Worsening in Clinical and Experimental Multiple Sclerosis

Silvia Caioli ^1^, Francesca De Vito ^1^, Sara Balletta ^2^, Alessandra Musella ^3^, Diego Fresegna ^3^, Mario Stampanoni Bassi ^1^, Luana Gilio ^1^, Valentina Vanni ^3^, Livia Guadalupi ^3^, Francesca Romana Rizzo ^2^, Krizia Sanna ^2^, Antonietta Gentile ^3^, Fortunata Carbone ^4^, Claudio Procaccini ^4^, Giuseppe Matarese ^4^, Diego Centonze ^1^, Georgia Mandolesi ^3^

^1^  IRCCS INM-Neuromed, Unit of Neurology, Pozzilli, Italy 

^2^  Tor Vergata University, Department of Systems Medicine, Rome, Italy 

^3^  IRCCS San Raffaele Roma, Synaptic Immunopathology Lab, Rome, Italy 

^4^  Consiglio Nazionale delle Ricerche, Laboratorio di Immunologia, Naples, Italy

Multiple sclerosis (MS) is a chronic autoimmune demyelinating disease with an unpredictable course also influenced by lifestyle factors. Increasing evidence, obtained in both MS and its mouse model, the experimental autoimmune encephalomyelitis (EAE), has revealed that proinflammatory cytokines and miRNAs trigger reversible synaptic dysfunctions as early hallmarks of the disease. The persistence of this inflammatory synaptopathy can cause excitotoxic damage and neuronal death, contributing to a silent disease progression independent of demyelination.

Considering that nutritional overload has recently been shown to enhance chronic inflammation in several autoimmune diseases, we aimed at clarifying its effects on neuroinflammation and synaptic damage in EAE and MS.

We explored the impact of high-fat diet (HFD) compared to standard diet (SD) in EAE and control mice by evaluating clinical, behavioral, electrophysiological and molecular parameters. As expected, HFD-obesity worsened EAE clinical score and EAE-dependent weight loss. Importantly, our results also indicated that HFD significantly increases striatal inflammation and excitatory transmission (sEPSC) in both control and EAE mice. In particular, control mice fed on HFD showed an enhancement of both sEPSC frequency and duration, reproducing the striatal synaptopathy observed in EAE SD mice.

Moreover, fecal 16S rDNA sequencing revealed that HFD induces gut microbiota dysbiosis in control mice and exacerbates it in EAE mice. Mechanistically gut dysbiosis likely alters immune homeostasis and increases inflammatory synaptopathy.

Clinical studies have confirmed the high synaptotoxic potential of HFD by showing serum triglyceride levels to directly correlate with both CSF glutamate levels and MS severity (EDSS) at diagnosis.

Overall, we demonstrated that HFD negatively affects EAE and MS course by altering glutamate signaling and inflammatory synaptopathy with an exacerbation of the consequent neurodegenerative processes.

## 105. Evaluation of Early Aging Following Perinatal Inflammation-Driven Encephalopathy of Prematurity in a Mouse Model

David Guenoun ^1,2^, Irvin Sautet ^1^, Solène Ruinet ^1,3^, Valerie Faivre ^1^, Jennifer Hua ^1^, Pierrette Young-Ten ^1^, Alice Jacquens ^1^, Mickael Tanter ^3^, Pierre Gressens ^1^, Juliette Van Steenwinckel ^1^, Homa Adle-Biassette ^4^, Cindy Bokobza ^1^

^1^  Inserm, U1141 Neurodiderot, Paris, France 

^2^  Department of Pharmacy, AP-HP, Robert Debré Hospital, Paris, France 

^3^  Physics for Medicine Paris – Inserm, ESPCI Paris-PSL, CNRS, Paris, France 

^4^  Université de Paris, service d’Anatomie Pathologique, AP-HP, Hôpital Lariboisière, DMU DREAM, UMR 1141, INSERM, Paris, France

Background: 15 million infants are born prematurely every year, 40% of the time following maternal infections. The resulting neuroinflammatory processes affecting these newborns are driven by glial cells’ reactivity (microglia and astrocytes) and increase the onset of brain lesions collectively termed encephalopathy of prematurity (EoP). EoP is associated with neurodevelopmental disorders in children and, in recent studies, with mental deficits such as mood disorders in young adults. Evidence demonstrated that long after initial reactivity, glial cells are keener to react exaggeratedly to later inflammatory stimuli, most probably through cellular priming. Recent literature focusing on the normal aging brain highlighted a low-grade chronic inflammatory state playing a potential role in the brain’s susceptibility to neurodegeneration. Glial cells primed by perinatal inflammation could therefore increase the age-related inflammation state leading to early aging.

Aims: We sought to determine whether a perinatal inflammatory challenge could accelerate the brain aging trajectory.

Methods: This study is based on a mouse model of perinatal inflammation responsible for EoP-like lesions. Months later, at the onset of EoP, in middle-aged mice, we used transcriptomic (RNA sequencing), functional (ultra-fast Doppler imaging, flow cytometry, histology), and behavioral analyses (open-field, three-chamber test, etc.) to evaluate the impact of perinatal inflammation on age-related inflammation and neuronal impairments, including functional brain connectivity and its behavioral consequences.

Results: Our results showed signs of ongoing inflammation and glial reactivity while brain connectivity defects were recorded in middle-aged mice exposed to perinatal inflammation.

Conclusions: Preliminary data tended to confirm long-term consequences of perinatal inflammation suggesting early brain aging.

## 106. Targeting the Brain 5-HT7 Receptor to Prevent Hypomyelination in a Rodent Model of Perinatal White Matter Injuries

Cindy Bokobza, Alice Jacquens, David Guenoun, Valérie Faivre, Gressens Pierre and Van Steenwinckel Juliette

Inserm, NeuroDiderot, Université de Paris, Paris, France

Children born prematurely (one birth out of 10) are at higher risk of developing perinatal brain lesions, especially white matter injuries (WMI). Evidence demonstrates that systemic inflammation-induced microglial and astrocyte reactivity are the prominent processes of WMI. Thus, a new challenge is to develop new neuroprotective strategies to target neuroinflammation to prevent WMI. Serotonin (5-HT) and its receptors play an important role in inflammation and emerging evidence indicates that 5-HT may regulate brain inflammation by the modulation of microglial reactivity and astrocyte functions. The present study is based on a mouse model of WMI induced by intraperitoneal (i.p.) injections of IL-1β during the first five days of life. In this model, certain key lesions of preterm brain injuries can be summarized by (i) systemic inflammation, (ii) pro-inflammatory microglial and astrocyte activation, and (iii) inhibition of oligodendrocyte maturation, leading to hypomyelination. We demonstrate that Htr7 mRNA (coding for the 5-HT7 receptor) is significantly overexpressed in the anterior cortex of IL-1β-exposed animals, suggesting it as a potential therapeutic target. LP-211 is a specific high-affinity HTR7 agonist that crosses the blood–brain barrier (BBB). When co-injected with IL-1β, LP-211 treatment prevented microglial and astrocyte reactivity and the down-regulation of myelin proteins (MBP and PLP) linked to hypomyelination. Thus, HTR7 may represent an innovative therapeutic target to protect the developing brain from preterm brain injuries.

## 107. Inflammatory Pathways Signal Transducers Analysis in iPSC-Derived Neurons and 3D Cerebral Organoids

Rosalba Monica Ferraro ^1^, Elena Laura Mazzoldi ^1^, Michele Manganelli ^1^, Marta Parigi ^1^, Giovanna Piovani ^1^, Daniele Moratto ^2^, Jessica Galli ^3^, Marco Cattalini ^4^, Elisa Fazzi ^3^ and Silvia Clara Giliani ^1^

^1^  Università degli Studi di Brescia, Department of Molecular and Translational Medicine, Brescia, Italy 

^2^  ASST Spedali Civili Brescia, Dipartimento di Diagnostica di Laboratorio, Brescia, Italy 

^3^  ASST Spedali Civili Brescia, Unit of Child Neurology and Psychiatry, Brescia, Italy 

^4^  ASST Spedali Civili Brescia, Pediatric Clinic, Brescia, Italy

Aicardi-Goutières syndrome (AGS) is a rare severe genetic disorder characterized by constitutive type I interferon (IFN) upregulation due to mutations in genes involved in nucleic acid metabolism and sensing. Clinically, AGS is characterized by microcephaly, brain atrophy and leukodystrophy. To date, some treatments with immuno-modulatory drugs that block interferon alpha (IFNa) signaling seem to improve the immunological conditions of patients, but the lack of therapies for their neurological degeneration is particularly pressing. The inaccessibility of autologous neurons to test new pharmacological compounds is hindering improvements in the field. The possibility to derive neurons from inducible pluripotent stem cells (iPSCs) obtained from patients offers the opportunity for in vitro modeling. We have deepened the in vitro 2D neuronal differentiation, generating and characterizing neural stem cells (NSCs) and neurons from iPSCs of three AGS patients mutated in different genes. Given the central role of IFNa in AGS, we investigated the IFNa signaling in NSCs, analyzing STAT1 as its principal signal transducer. Despite what we observed in lymphocytes and monocytes of AGS patients that showed a statistically significant increase of STAT1 activation and expression, NSCs seem to be anergic to IFNa stimulation. No increment of STAT1 activation was in fact detected in NSCs, while STAT1 expression was still present in iPSC-derived NSCs. Our results suggest that neuronal degeneration observed in AGS could not be caused directly by IFNa but can be a consequence of an unhealthy state of glia, which, stimulated by IFNa, assumes the antiviral phenotype that impairs the typical glia functions such trophic support and myelination. Moreover, we generated 3D cerebral organoid in dynamic suspension from control and AGS iPSCs as a better in vitro model to explore the pathogenetic contributions and interaction between neurons and glia.

## 108. Enhancing Dendritic Spine Plasticity by Coupling Physical Activity with Non-Invasive Brain Stimulation

Federica Marchiotto, Arianna Di Giorgio, Marco Cambiaghi and Mario Rosario Buffelli

University of Verona, Neuroscience, Biomedicine and Movement, Verona, Italy

There is a huge interest in coupling transcranial direct current stimulation (tDCS) and physical activity as an effective strategy to further enhance cortical excitability in physiological and pathological conditions. Nevertheless, the mechanisms underlying this phenomenon are not well understood yet. Animal studies revealed that tDCS affects the motor cortical plasticity by modulating dendritic spines, similarly to voluntary physical exercise. Thus, in this study we investigated the effects of combining tDCS and physical activity in healthy mice. For this purpose, we studied the effects of coupling anodal tDCS and physical activity on the morphological plasticity in primary motor cortex (M1) layer II/II and layer V in both young (2–3 months) and middle-aged (14–16 months) mice. At both ages, the combination of stimulation and physical activity results in an increased number of activated cells and in a higher density of spines in basal and apical dendrites of both hemispheres, compared to single interventions only. In young mice spine morphology analysis highlighted increased mushrooms spines, while middle-aged mice showed a higher number of thin spines after the association of tDCS and physical activity. Altogether, the coupling between tDCS and physical activity results in a significant inter-hemispheric plasticity enhancement in physiological conditions, maintained with aging. However, the spine morphology is differently displayed in young and middle-aged mice, probably indicating a different effect of the combination in the aging.

## 109. Exploring the Dynamics of Cell Excitability by Optogenetics in Ex Vivo Neuronal Cultures

Elena Gjorgievska and Michele Giugliano

International School for Advanced Studies, SISSA, Neurobiology, Trieste, Italy

It is currently unclear whether or how neuronal excitability dynamics differ between types of neurons over extended (little-studied, yet highly relevant) timescales.

We approach the subject via a medium-throughput methodology, coupling extracellular recording with optogenetic stimulation of subpopulation specific, ShChR/CaMKIIα-expressing i.e., putative glutamatergic cells. We analyze spike probability and spike latency as proxies of intrinsic excitability.

In accordance with previous findings, across a wide range of frequencies of repeated photoactivation, we report a transient and an intermittent phase. Based on the latter, we classify the considerable heterogeneity of responses. These observations question the validity of the established glutamatergic type, which is essential for understanding the computations it performs and its role within a circuit. Furthermore, we find the macroscopic properties of the evoked spike trains to be identical under different stimulation regimes above a critical stimulation rate. We show that the fluctuations in the spike probability which occur over extended observation windows follow the statistics of a fractional random process, indicating that intrinsic neuronal activity is significantly affected by past stimuli.

Our work is crucial to the development of more accurate generative models which are capable of accurate and versatile description of neuronal excitability: (a) across spatiotemporally diverse electrophysiological types and (b) over behaviorally relevant, extended timescales.

## 110. Tetanus Toxin Injections into the Rat Motor Cortex and Striatum Impair the Narrow Beam Walking Performance

Patrik Meglić, Petra Šoštarić, Davor Virag and Ivica Matak

School of Medicine, University of Zagreb, Pharmacology, Zagreb, Croatia

Abnormalities in the motor cortex and basal ganglia excitability play a central role in the onset of movement disorders such as dystonias and parkinsonism. Basal ganglia and motor cortex integrate the sensory proprioceptive input arriving from the periphery for the planning and execution of movement. Tetanus neurotoxin (TeNT) prevents the inhibitory neurotransmission in central synapses. The aim of present the study was to examine the behavioral effect of neuronal disinhibition in mentioned brain regions induced by low, non-convulsive doses of TeNT. The rats were unilaterally injected into the caudate putamen or motor cortex with 0.5 ng TeNT. The injections were repeated into the contralateral motor regions after two weeks, and the effect of TeNT was assessed for another two weeks. Different behavioral tests were performed repeatedly to assess the effect of TeNT induced disinhibition on rat motor performance, as well as to exclude possible epileptogenic action of tetanus neurotoxin. After unilateral toxin applications, the animals appear to be able to compensate for the proprioceptive motor deficit, which is then aggravated and becomes longer lasting after contralateral regional disinhibition. The plantar misplacement of the hind-limb during the narrow beam traversing were more evident on the hind-paw contralateral to the injected brain region. Open field test, pre-pulse inhibition, and attempted audiogenic seizure tests did not indicate the possible epileptogenic actions of TeNT. The motor cortex or striatal disinhibition with TeNT induces subtle motor impairment during the relatively complex motor task of crossing the elevated narrow beam, requiring correct prediction of paw placement. Since only 50% of treated animals have developed the described impairments, it is necessary to make additional experiments. Experiments with higher doses of TeNT or application into the different regions, such as the internal globus pallidus, are necessary.

## 111. Cancer-Neuronal Crosstalk in Glioblastoma

Chiara Saulle, Elisabetta Stanzani, Filippo Mirabella, Erica Tagliatti, Edoardo Fraviga, Michela Matteoli, Lorena Passoni and Davide Pozzi

Humanitas Research Center, Department of Biomedical Science, Rozzano (Milano), Italy

Glioblastoma (GBM) is a highly aggressive and invasive brain tumor with rather unique features. Interestingly, it is of recent acquisition that GBM also exploits neuronal activity as fuel to boost proliferation and aggressiveness and it induces hyperexcitability of surrounding network creating a vicious cycle. However, the mechanisms and the specific contribution of brain cells to the interplay between neurons and cancer cells are largely unknown. We set up an in vitro co-culture model to study the molecular mechanisms underlying the GBM-neuronal crosstalk. Primary human GBM stem-like cell lines (GSCs) were established from patient post-surgical specimens. To model the neuro-tumoral unit, GSCs were cocultured with primary neurons at either immature (4 DIV) or mature (11 DIV) stages. After seven days of coculture, both immature and mature neuronal cultures boosted GSCs proliferation. In addition, neuronal conditioned medium or astrocytes alone were not able to sustain cancer cell proliferation, likewise suggesting a putative mechanism based on cell-to-cell interaction.

To explore the possible causes, neuronal network activity was measured in presence/absence of tumor cells using High Density-Multi Electro Array. After 24 h of neuron-cancer co-culture, the mean firing rate of neurons was increased indicating that GSCs promote network excitability. By using the genetically encoded intracellular glutamate-sensitive fluorescent sensor (iGluSnFR), we found that GSCs were able to sense electrically evoked glutamate released from neurons, with kinetic properties resembling those observed in neuron-to-neuron synapses. In summary, here, we described a GBM-neurons vicious cycle in which neurons boost GSCs proliferation and cancer cells trigger neuron hyperexcitability, probably involving glutamate released by neuronal activity. These results represent an entry point to investigate the molecular mechanisms underpinning cancer-neuronal crosstalk.

## 112. Reduction of Lipoprotein Receptors Levels Synergistically Potentiates the Anti-Tumour Activity of Givinostat on Human Glioblastoma Cancer Cells

Lorenzo Taiarol ^1^, Chiara Bigogno ^2^, Silvia Sesana ^1^, Marcelo Kravicz ^1^, Francesca Viale ^1^, Eleonora Pozzi ^3^, Laura Monza ^4^, Valentina Alda Carozzi ^3^, Cristina Meregalli ^3^, Silvia Valtorta ^5^, Rosa Maria Moresco ^1^, Marcus Koch ^6^, Federica Barbugian ^1^, Laura Russo ^1^, Giulio Dondio ^2^, Christian Steinkühler ^7^, Francesca Re ^1^

^1^  University of Milano-Bicocca, BioNanoMedicine Center NANOMIB, School of Medicine and Surgery, Vedano al Lambro (MB), Italy 

^2^  Aphad srl, Aphad srl, Buccinasco (MI), Italy 

^3^  University of Milano-Bicocca, Experimental Neurology Unit, School of Medicine and Surgery, Vedano al Lambro (MB), Italy 

^4^  University of Milano-Bicocca, Experimental Neurology Unit, School of Medicine and Surgery, Vedano al Lambro (MB), Italy 

^5^  National Research Council (CNR), Institute of Bioimaging and Molecular Physiology (IBFM), Segrate (MI), Italy 

^6^  INM–Leibniz Institute for New Materials, Campus D2 2, Saarbrücken, Germany 

^7^  Italfarmaco SpA, Italfarmaco SpA, Cinisello Balsamo (MI), Italy

Dysregulation of histone modifying enzymes (HDACs) is commonly identified in many tumors and has been linked to cancer proliferation, changes in metabolism and drug resistance. These events also sustain the onset and progression of glioblastoma (GBM), the most common and aggressive brain tumor. Accordingly, HDAC inhibitors (HDACis) represent a promising class of anti-tumor agents. In this context, we analyzed the activity of Givinostat, a pan-HDACi, in a GBM cell model. The treatment of GBM cells with Givinostat inhibited HDACs activity, affected cell viability in a dose- and time-dependent manner and induced caspase-mediated cell death. Givinostat also display a natural selectivity for cancer cells versus healthy cells that was maintained up to the dose of 2.5 µM. In addition, the expression levels of several receptors involved in cholesterol uptake (low-density lipoprotein receptor, very low-density lipoprotein receptor and low-density lipoprotein receptor-related protein 1) were significantly decreased, unravelling an unprecedented mechanism of action of Givinostat on GBM cells. This effect was confirmed using ApoE-lipoprotein-like particles in 2D and 3D cellular models, providing evidence of the key role played by HDACs in tumor metabolism. We also provide the proof of concept for a delivery system that can improve the pharmacokinetic of Givinostat. Liposomes composed of cholesterol and sphingomyelin embedding Givinostat showed a 2.5-fold increase in the drug plasma half-life and a six-fold increase of the drug brain exposure in healthy mice. These results strongly suggest that Givinostat may have a clinical potential for HDACi-based therapeutic strategies against GBM and liposome valuable candidates for its brain delivery. IMMUNHUB “Sviluppo di nuove molecole di seconda generazione per immunoterapia oncologica”, CUP E51B19000550007–Call HUB Ricerca e Innovazione, cofunded by POR FESR 20142020 (Regional Operational Programme, European Regional Development Fund).

## 113. Molecular Changes Underlying Decay of Sensory Responses and Enhanced Seizure Propensity in Peritumoral Neurons

Marta Scalera ^1^, Elena Tantillo ^1^, Elisa De Santis ^2^, Chiara Cerri ^3^, Chiara Maria Mazzanti ^4^, Eleonora Vannini ^5^ and Matteo Caleo ^6^

^1^  Consiglio Nazionale delle Ricerche-Pisa, Istituto di Neuroscienze, Pisa, Italy 

^2^  CNR, Area di Pisa, Istituto di Neuroscienze, Pisa, Italy 

^3^  Università di Pisa, Dipartimento di Farmacologia, Pisa, Italy 

^4^  Fondazione Pisana per la Scienza ONLUS, Laboratorio di Genomica e Trascrittomica, Pisa, Italy 

^5^  Consiglio Nazionale della Ricerche-Pisa, Istituto di Neuroscienze, Pisa, Italy 

^6^  Università di Padova, Dipartimento Di Scienze Biomediche, Padova, Italy

In recent years, the interaction between glioma and brain cells has emerged as one important regulator of tumor progression. In particular, it has been proved that glioma growth impacts the structure and physiology of peritumoral neuronal networks, altering the activity of pyramidal neurons which drives further tumor progression. Using the GL261 syngeneic murine model, we performed in vivo electrophysiological recordings of visual evoked potentials (VEPs) to longitudinal assess modifications of peritumoral neurons along with glioma progression. With respect to controls, glioma-bearing mice showed a dampening of visual responses that started from day 14 after tumor induction (TI). At this stage, we microdissected layer II-III pyramidal neurons and evaluated the expression of a panel of genes involved in synaptic transmission and neuronal excitability. Among all genes, only gabra1 and SNAP25 were significantly reduced in peritumoral neurons from glioma-bearing mice. No significant changes were detected in glutamatergic markers. We also performed LFPs recordings in freely moving glioma-bearing and control mice. We found interictal spikes in 50% of glioma-bearing mice 18 days after TI. An intraperitoneally treatment with a subconvulsive dose of DMCM triggered epileptiform activity in glioma-bearing mice but not in controls, suggesting an involvement of the GABA-A receptor in seizures’ susceptibility. Elucidating the mechanisms underlying the decay of the sensory response and the propensity to seizures in glioma-bearing animals could add useful information to develop more effective therapeutic approaches aimed at ameliorating patients’ quality of life and survival.

## 114. An Unexpected Culprit: Intracerebellar Hemorrhage in At-Term Newborn

Roberta Pintus ^1^, Valentina Masile ^2^, Maria Cristina Pintus ^2^, Maria Antonietta Marcialis ^2^ and Vassilios Fanos ^1^

^1^  Università degli studi di Cagliari, Department of Surgery Neonatal Intensive Care Unit AOU Cagliari, Cagliari, Italy 

^2^  Azienda Ospedaliera Universitaria di Cagliari, Policlinico di Monserrato, Neonatal Intensive Care Unit, Cagliari, Italy

Cerebellar hemorrhage in at term neonates is very rare. In the past, it was usually diagnosed postmortem, but nowadays, its detection is increasing thanks to the new radio-imaging techniques. It is associated to traumatic birth (breech presentation), prolonged labor, vacuum application, and maternal factors, such as infections. They could be due to severe distortion of the venous structures with laceration of the falx or to direct cerebellar damage with vermis laceration. Clinically, the patients could present severe asphyxia requiring intubation and seizures. The outcomes are relatively unknown, but they can vary from normal to severe disabilities, including motor alterations, cognitive and psychiatric disorders, and autism.

In our case, the neonate had a gestational age of 39 + 5 weeks (spontaneous delivery with a labor of 18 h and double-wrapped cord around the neck) and mild hypoxic ischemic encephalopathy at birth. He was hypotonic, lethargic, with reduced motility and reflexes. He was treated with whole body hypothermia and the cerebral function monitoring was started. The electroencephalogram was moderately altered. After some hours the patient improved and was extubated.

On the second day of life, the patient suffered from severe apnea requiring intubation and drug-resistant seizures (phenobarbital). Thus, levetiracetam was added. The ultrasound detected an intraventricular hemorrhage. While the magnetic resonance imaging highlighted a bilateral intraparenchymal cerebellar hemorrhage (18.8 × 10 mm in the right hemisphere and 9.8 × 5 mm in the left hemisphere), a subdural hemorrhage in the same region and an intraventricular hemorrhage was also detected. Then, the patient recovered his respiratory function, with amelioration of his general conditions and was discharged after two weeks.

At two months of life, during the follow up examination, the patient displayed a normal psycho-behavioral and motor profile.

## 115. In Vivo Evaluation of Dentato-Thalamo-Cortical Tract Integrity in Friedreich Ataxia Using Diffusion MRI

Mario Tranfa ^1^, Giuseppe Pontillo ^1^, Sara Bosticardo ^2^, Chiara Pane ^3^, Simona Schiavi ^2^, Giulia Biolo ^2^, Alessandro Daducci ^2^, Francesco Saccà ^3^, Arturo Brunetti ^1^, Nellie Georgiou-Karistianis ^4^, Ian Harding ^5^ and Sirio Cocozza ^1^

^1^  University of Naples “Federico II”, Department of Advanced Biomedical Sciences, Naples, Italy 

^2^  University of Verona, Diffusion Imaging and Connectivity Estimation (DICE)Lab, Department of Computer Science, Verona, Italy 

^3^  University of Naples “Federico II”, Department of Neurosciences and Reproductive and Odontostomatological Sciences, Naples, Italy 

^4^  Monash University, School of Psychological Sciences and Turner Institute for Brain and Mental Health, Melbourne, Australia

^5^  Monash University, Monash Biomedical Imaging, Melbourne, Australia

Background and Objective • Brain involvement in Friedreich Ataxia (FRDA) is characterized by widespread microstructural alterations, extending beyond brainstem and cerebellum. Nevertheless, no information about the degree of involvement of the dentato-thalamo-cortical tract (DTT), the cerebellar motor system main efference, is available. The aim of this study was to explore the microstructural integrity of this tract in FRDA using diffusion MRI (dMRI).

Methods • Scans of 57 FRDA and 52 healthy controls (HCs) from three different sites were evaluated. In all subjects, a volumetric T1-weighted sequences, for brain parcellation purposes, and a high resolution dMRI sequence, for the quantification of DTT bundles, were obtained. Tracts computation was obtained as follows: fibers connecting each dentate nucleus (DN) to the contralateral thalamus, encompassing ipsilateral red nucleus and ending in the primary motor cortex were calculated for each HC. A study specific template was calculated as the average of all tracts, and then applied to each patient’s space to extract microstructural indices of bundle integrity (fractional anisotropy -FA-, radial -RD- and mean diffusivity -MD-).

Results • After excluding subjects with poor image quality, data of 50 FRDA patients (mean age 34.8 ± 13.9; M/F = 29/21) and 38 HCs (mean age 36.1 ± 12.7; M/F = 18/20) were compared. A significant decrease in FA in FRDA, compared to HCs, emerged on both sides (0.38 ± 0.03 vs. 0.42 ± 0.02, on the left; 0.39 ± 0.03 vs. 0.43 ± 0.02, on the right, *p*-values < 0.001), coupled to a significant increase in MD and RD (all *p*-values < 0.001).

Discussion and Conclusion • Our analysis further expands the current knowledge about brain involvement in FRDA, by showing the presence of significant microstructural abnormalities at the level of the main cerebellar efference in these patients. This finding is in line with the hypothesis of an anterograde secondary degeneration arising from the DN to the primary motor cortex and corroborate the possibility of employing dMRI to longitudinally evaluate damage spread and possibly treatment response in FRDA.

## 116. Examination of Whole-Brain Structural and Functional Connectivity in Fabry Disease

Ilaria Gabusi ^1^, Giuseppe Pontillo ^2^, Maria Petracca ^3^, Metteo Battocchio ^1^, Sara Bosticardo ^1^, Teresa Costabile ^4^, Alessandro Daducci ^1^, Chiara Pane ^5^, Eleonora Riccio ^6^, Antonio Pisani ^6^, Arturo Brunetti ^7^, Simona Schiavi ^8^ and Sirio Cocozza ^7^

^1^  University of Verona, Department of computer science, Verona, Italy 

^2^  University of Naples “Federico II”, Department of Electrical Engineering and Information Technology, Naples, Italy 

^3^  Sapienza University of Rome, Department of Human Neuroscience, Rome, Italy 

^4^  ‘’Luigi Vanvitelli” University, Department of Clinical and Experimental Medicine, Naples, Italy 

^5^  University of Naples “Federico II”, Department of Neurosciences and Reproductive and Odontostomatological Sciences, Naples, Italy 

^6^  University of Naples “Federico II”, Department of Public Health, Naples, Italy

^7^  University of Naples “Federico II”, Department of Advanced Biomedical Sciences, Naples, Italy 

^8^  University of Genoa, Department of Neuroscience, Rehabilitation, Ophthalmology, Genetics, Maternal and Child Health, Genoa, Italy

In Fabry disease (FD), a rare X-linked lysosomal storage disorder, the central nervous system is heterogeneously involved but the macro-scale connectivity is not yet been investigated. In this study, we processed diffusion and resting-state functional MRI data of 46 patients (FDs, 28F, 42.2 ± 13.2 y) and 49 healthy controls (HCs, 21F, 42.3 ± 16.3 y). To build structural connectomes (SC), we employed probabilistic tractography and convex optimization modeling for microstructure informed tractography, weighting each connection by the total intra-axonal signal fraction of the corresponding bundle. Functional connectomes (FC) were built by computing the correlation between BOLD timeseries and using a modified AAL parcellation with 100 regions (used also for SC). By exploring the between-group differences in terms of five global network metrics extracted for each brain network, we found that FDs have a significantly reduced global efficiency (*p* = 0.005) and mean strength (*p* < 0.001) in SC and an increased modularity (*p* = 0.005) in FC. Moreover, we employed network-based statistics to explore for the presence of connected subnetworks associated with a significant between-group difference. As result, we identified a subnetwork, involving mainly frontal areas, with decreased structural connectivity in FDs w.r.t. HCs. Finally, we tested the relationship between the altered properties of SC and FC and the cognitive performance of a FDs subset (n = 11). Significant correlations arose between SC mean strength and RAVLT-immediate score (r = 0.72, *p* = 0.03) and between FC modularity and DGS score (r = −0.77, *p* = 0.02). Instead, the subnetwork showed a mean structural disruption of –0.62 in FDs compared to HCs, which resulted significantly correlated to three different neuropsychological tests (WCFST, CBTT and RAVLT-delayed). These findings show widespread structural disconnection and functional reorganization in FDs, supported by loss in axonal integrity and with some associations with cognitive performance.

## 117. The β Amyloid-Derived Peptide Aβ1-6A2V Protects from Tau Toxicity In Vivo

Carmina Natale ^1^, Gloria Vegliante ^2^, Federico Moro ^2^, Federica Marta Xodo ^1^, Laura Colombo ^1^, Ada De Luigi ^1^, Alfredo Cagnotto ^1^, Fabrizio Tagliavini ^3^, Marcella Catania ^3^, Giuseppe Di Fede ^3^, Elisa R. Zanier ^2^, Mario Salmona ^1^ and Luisa Diomede ^1^

^1^  Istituto di ricerche farmacologiche Mario Negri-IRCCS, Molecular Biochemistry and Pharmacology, Milano, Italy 

^2^  Istituto di ricerche farmacologiche Mario Negri-IRCCS, Neuroscience, Milano, Italy 

^3^  Fondazione IRCCS Istituto Neurologico Carlo Besta, Milan, Italy, Neurology 5/Neuropathology, Milano, Italy

Altered amyloid β (Aβ) and tau species are the hallmarks of Alzheimer’s disease (AD) and the primary targets for therapeutic intervention. Although several attempts to find a treatment for AD have been made over the years, there are still no effective therapies, and the drugs available provide only modest symptomatic benefit. To design an innovative pharmacological strategy, we took inspiration from a clinical observation on a subject naturally protected from the onset of a genetic AD form. In this subject, the 673 Ala-to-Val substitutions in heterozygous form in the APP gene resulted in the production of an Aβ carrying A2V mutation able to interact with Aβ wild-type, thus interfering with its polymerization. Based on this observation, our group developed the Aβ1-6A2V synthetic peptide able to interfere in vitro and in vivo with Aβ polymerization and protect from its proteotoxicity.

To test whether this peptide can also interfere with tau aggregation and toxicity, we applied an integrated approach involving recombinant human tau, the nematode C. elegans, and two murine models of tauopathy: the transgenic P301L mice and 3xTg-AD mice subjected to traumatic brain injury (TBI). Aβ1-6A2V inhibited tau aggregation in vitro and counteracted the toxicity induced in C. elegans by brain homogenates from P301L mice. The intranasal administration of Aβ1-6A2V to injured 3xTg-AD mice caused a reduction in the TBI-induced cognitive impairment. Notably, brain homogenates from Aβ1-6A2V treated animals were not toxic when administered to C. elegans, indicating that the peptide reduced the cerebral toxic forms of tau in mice.

These findings indicate for the first time that Aβ1-6A2V can interact with tau in vitro and in vivo, protecting from its toxic effects, suggesting that this peptide can be an ideal therapeutic strategy for treating not only AD, but also other tauopathies.

## 118. Emerging Roles of SLITRK Family Members in αSyn- p.A53T Mediated Synaptic Dysfunction

Elissavet-Kalliopi Akrioti ^1^, Katerina Segklia ^1^, Aristofanis Stavrou ^1^, Panagiotis Chandris ^1^, Konstantina Charbi ^1^, Paraskevi Koutsoudaki ^2^, Sofia Havaki ^2^, Martina Samiotaki ^3^, George Panayotou ^3^ and Era Taoufik ^1^

^1^  Hellenic Pasteur Institute, Laboratory of Molecular & Cellular Neurobiology- Stem Cells, Athens, Greece 

^2^  Medical School, NKUA, Laboratory of Histology-Embryology, Athens, Greece 

^3^  B.S.R.C. Alexander Fleming, Institute for Bioinnovation, Athens, Greece

Alpha-synuclein (αSyn) is a highly expressed and conserved pre-synaptic protein, which when dysregulated leads mainly to sporadic Parkinson’s disease (PD). Familial forms of the disease also exist and are due to mutations in the SNCA gene that encodes αSyn. The best-characterized mutation of αSyn is the p.A53T (G209 SNCA), which leads to early onset progressive parkinsonism and is studied in numerous mouse and human-based experimental systems. Our team employing a human induced pluripotent stem cell (iPSC)-based model that harbors the p.A53T mutation and displays PD-associated phenotypes, showed early distortions in a variety of pathways, including those that are related to synaptogenesis. Of relevance, transcriptomic analysis suggested defects in synapse formation and function and showed dysregulated expression of genes involved in synaptic signaling. Here, we try to investigate the potential role of three members of the post-synaptic adhesion molecules family, so called-SLITRKs in p.A53T-αSyn mediated synaptic dysfunction. A transgenic mouse model that expresses the human p.A53T-αSyn and hiPSC-derived neurons is used to examine the ultrastructural and molecular defects of the p.A53T synapse and establish the link between pathological αSyn expression and SLITRKs expression and subcellular localization. E.M. analysis of p.A53T synapses showed distorted organization and fewer synaptic vesicles compared to control synapses. Additionally, artificial synapse formation assays revealed defects in early synaptogenesis as p.A53T neurons formed more inhibitory synapses than control neurons, while excitatory synapse formation remained unaffected. This imbalance was also obvious when naturally forming p.A53T synapses were analyzed. Furthermore, dysregulation in SLITRK1/2/4 RNA and protein expression was observed from early stages of p.A53T pathology while subneuronal localization was also greatly affected. Altogether, our work aims to identify the link between SLITRKs dysregulation and p.A53T-αSyn induced synaptopathy and to characterize the molecular and cellular mechanisms.

## 119. Sleep Fragmentation Accelerate Dementia in Transgenic 5xFAD AD Mice Model Inducing Astrogliosis and Affecting Glymphatic System

Valeria Vasciaveo ^1^, Antonella Iadarola ^2^, Antonino Casile ^3^, Davide Dante ^4^, Lorenzo Minotta ^4^, Giulia Morello ^4^, Elena Tamagno ^1^, Alessandro Cicolin ^2^ and Michela Guglielmotto ^1^

^1^  Neuroscience Institute Cavalieri Ottolenghi, University of Turin, Neuroscience Rita Levi Montalcini, Torino, Italy 

^2^  University of Turin, Neuroscience Rita Levi Montalcini, Torino, Italy 

^3^  University of Camerino, School of Pharmacy, Pharmacology unit, Camerino, Italy 

^4^  Neuroscience Institute Cavalieri Ottolenghi, University of Turin, Torino, Italy

Alzheimer’s disease (AD), the most common form of dementia, is characterized by genetic and multifactorial risk factors. Many studies correlated AD to sleep disorders. The acute effect of sleep disorders results in an increase in amyloid-b (Ab) concentration, due to a decrease in its clearance, besides the sleep quality in AD patients is impaired, leading to a possible further accumulation of Ab. In this study, we performed and validated a mouse model of AD and sleep fragmentation in 5xFAD mice of two months of age. All the animals underwent to behavioral studies to analyze anxiety and spatial and working memory. We had validated sleep fragmentation and its effect through EEG and biomolecular analysis, by observing all the macro-areas implied in sleep regulation. As regard behavioral activities, we observed a significant memory impairment and an increase in anxiety in fragmented mice compared to control. These results were confirmed in biomolecular analysis. In particular, Ab accumulation increased in all the interested areas, while tau phosphorylation appeared only in the dentate gyrus, and these data correlate to a significant increase in neuroinflammation, evaluating both microglia and astrocyte markers. Aquaporin-4 (AQP4), the astrocyte transporter for Ab clearance, was significantly increased in fragmented mice compared to control. This is not evident analyzing older 5xFAD mice (six months old) which underwent sleep fragmentation, where we observed a decrease of AQP4 levels without differences in the density of GFAP. In conclusion, we can assert that sleep fragmentation worsens AD pathology by accelerating Ab accumulation, which in turn triggers neuroinflammation and tau phosphorylation, but there is still an attempt from the brain to rescue the faster progression of the pathology by increasing AQP4 levels. Meanwhile, in an advanced pathological system in six-month-old mice, the AQP4 clearance mechanism fails, perhaps due to the decreased expression of the channel.

## 120. The Role of Astrocytes in β-Amyloid- and Magnetite Nanoparticles-Induced Neurotoxicity

Veronica D’Ezio ^1^, Marta Russo ^1^, Andrea Sperati ^2^, Ludovica Carpinelli ^1^, Roberta Balzani ^1^, Marco Colasanti ^1^, Tiziana Persichini ^1^

^1^  University “Roma Tre”, Department of Sciences, Roma, Italy 

^2^  University of Rome “Sapienza”, Department of Experimental Medicine, Rome, Italy

Air pollution is thought to be one of the causes of the increased occurrence of several neurodegenerative diseases such as Alzheimer’s. Very recent studies have shown that magnetite (iron oxide) nanoparticles (MNP), produced by urban traffic, can be inhaled and reach the brain. This ultrafine particulate matter is toxic to the brain as it can promote the formation of reactive oxygen species and induce oxidative stress, a condition often associated with Alzheimer’s disease and other neurodegenerative disorders. Iron has been shown to facilitate Amyloid-β (Aβ) deposition, a hallmark of Alzheimer’s disease, leading to neuronal damage. During oxidative stress, astrocytes can activate the transcription factor Nrf2 a regulator of several phase II detoxifying and antioxidant genes, such as the System Xc- subunit xCT. Here, we studied (i) the effect of the Aβ fragment 25–35 (Aβ25-35) and MNP on Nrf2-dependent System Xc- expression in U373 human astroglial cells and (ii) the effect of Aβ25–35- and MNP-induced astrocytic response on neuronal cell viability using an in vitro co-culture system. We found that Aβ25-35 as well as MNP were able to activate an antioxidant response in astrocytes, by inducing both Nrf2 activation and System Xc- up-regulation. However, this astrocytic response caused an enhanced cell mortality of co-cultured SH-SY5Y cells, taken as a neuronal model. Consistently, the specific System Xc- inhibitor sulfasalazine prevented the increase of both neuronal mortality and extracellular glutamate levels, thus indicating that the neurotoxic effect was due to an augmented release of glutamate through the transporter. The present study sheds light on the Nrf2/system Xc- pathway in the toxicity induced by Aβ25-35 and may help to better understand the involvement of astrocytes in neuronal death during Alzheimer’s disease.

## 121. Compensatory Myogenesis and Acetylcholine Receptor Clustering Delay Symptoms Onset and Progression in SOD1 Mutant Mice

Cassandra Margotta ^1^, Paola Fabbrizio ^1^, Jessica D’Agostino ^1^, Chiara Cambieri ^2^, Marco Ceccanti ^2^, Eleonora Palma ^3^, Maurizio Inghilleri ^2^, Giovanni Nardo ^1^ and Caterina Bendotti ^1^

^1^  Istituto di Ricerche Farmacologiche Mario Negri, Department of Neuroscience, Milan, Italy 

^2^  Sapienza University of Rome, Department of Human Neurosciences, Rome, Italy 

^3^  Sapienza University of Rome, Department of Physiology and Pharmacology, Rome, Italy

Amyotrophic lateral sclerosis (ALS) is a heterogeneous disease with high variability in the speed of progression even in cases with a defined genetic cause such as superoxide dismutase 1 (SOD1) mutations. SOD1G93A mutation on mice with distinct genetic backgrounds (C57 and 129Sv) show consistent differences in speed of disease progression resembling what is observed in ALS patients. We recently hypothesized that the difference in the peripheral neuromuscular system rather than the extent of spinal motor neuron loss reflects the phenotypic difference between these two mouse models. Therefore, we redirect our attention to the skeletal muscle as an early component of ALS pathogenesis, aiming to discover the molecular mechanisms contributing to the distinct phenotypes and to identify factors underlying fast and slow disease progression. In this work, we compare the functional, morphological, and molecular profiles of the gastrocnemius muscle (GCM) from these two SOD1G93A mouse strains at the pre-symptomatic and onset stage of the disease. Data collected clearly defined the extent of NMJ stability and muscle regeneration as a discriminator between rapidly and slowing progressing ALS mice. Notably, the slow-progressing mice, despite the premature denervation and muscle atrophy, activate different compensatory mechanisms including the expression and clustering of the AChR, myogenesis and inflammatory response, which are able to delay the onset and progression of their symptoms. On the contrary, the fast-progressing mice that are unable to activate these responses exhibit a rapid decline of muscle force. This study highlights a set of key genes and molecular pathways indices of fast or slow disease progression, which may prove useful in identifying potential disease modifiers responsible for the heterogeneity of human amyotrophic lateral sclerosis, which may provide new opportunities to hamper the disease progression.

## 122. Single-Cell Transcriptomic Comparison of Human Microglia in Alzheimer’s Disease and Multiple Sclerosis

Edoardo Pedrini ^1^, Francesca Fagiani ^1^, Maria Sofia Martire ^1^ and Martina Absinta ^1,2^

^1^  Institute of Experimental Neurology, Division of Neuroscience, IRCCS San Raffaele Hospital and Vita-Salute San Raffaele University, Milan, Italy 

^2^  Department of Neurology, Johns Hopkins University School of Medicine, Baltimore, MD, USA

Microglia activation has been reported to play a significant role in the progression of neurodegenerative diseases such as Alzheimer’s disease (AD) and Multiple sclerosis (MS). This has raised some interest in identifying potential microglia commonalities and molecular targets to investigate further. Previous studies highlighted a partial overlap between the transcriptional profile of microglia from the whole brain of AD animal models (5xFAD mouse) and MS plaques. However, a direct comparison of human microglia from AD and MS patients is missing. Thus, we compared microglial transcriptomic profiles using human MS and AD single nucleus RNA-seq (snRNA-seq) datasets. In particular, we compared the entorhinal and frontal cortex from AD patients (Braak stage 2 and 6) vs. chronic active lesions from MS patients vs. non-neurological control brains. We filtered the microglia population from the selected datasets and performed differential gene expression (DE) comparing control and disease states. We defined consensus gene signatures for each disease state for both upregulated and downregulated sets from the list of DE genes. These signatures were used to score the microglia enrichment of AD vs. MS and MS vs. AD by GSEA. Interestingly, we have identified a strong enrichment score for both upregulated and downregulated signatures from AD on MS and vice versa. To further confirm the similarity between the two disease states, we generated pseudobulks and built a correlation matrix between samples. The clustering analysis also supports the hypothesis that microglia from AD Braak 2 and chronic active MS lesions have some transcriptional similarities. In conclusion, we provided initial evidence supporting the hypothesis of similarity concerning the transcriptomic profile of activated microglia between AD and MS in specific disease stages. The signatures and the leading edges defined in the specific gene scoring can be used as reference for experimental validation.

## 123. n-3 PUFA Improves Psychological Well-Being during Menopausal Transition

Stefano Sacchetti ^1^, Davide Decandia ^2^, Eugenia Landolfo ^3^, Francesca Gelfo ^4^ and Debora Cutuli ^5^

^1^  IRCCS Fondazione Santa Lucia; Sapienza University of Rome, Department of Psychology, Rome, Italy 

^2^  IRCCS Fondazione Santa Lucia; PhD Program in Behavioral Neuroscience; Sapienza University of Rome, Department of Psychology, Rome, Italy 

^3^  Sapienza University of Rome, Department of Psychology, Rome, Italy 

^4^  IRCCS Fondazione Santa Lucia; Guglielmo Marconi University, Department of Human Sciences, Rome, Italy 

^5^  IRCCS Fondazione Santa Lucia; Sapienza University of Rome, Department of Psychology, Rome, Italy

Females show an increased risk of cognitive impairment when approaching menopause because of the loss of ovarian function and estrogen deficiency occurring during the climacteric. In addition, menopause is closely associated with emotional disorders, such as anxiety and depression.

Data on risk and protection factors have yielded robust evidence on the effects of lifestyle factors, such as diet, in preserving emotional and cognitive functioning. The impact of specific lifestyle factors on psychological health indicates that there may be potential to improve (or at least stabilize) declining trajectories of emotional and cognitive functions in menopause.

This work focused on the effects of omega-3 polyunsaturated fatty acids (n-3 PUFA) supplementation on cognitive functions, depression, and anxiety during the menopausal transition.

This systematic review, performed according to PRISMA guidelines, considered all articles published until 31 December 2021 and the search was performed on two databases, PUBMED and SCOPUS. The fields of interest were “menopausal transition”, “n-3 PUFA”, and “cognitive and affective aspects”.

Out of the 361 articles found on PUBMED and 283 on Scopus, 17 met the inclusion criteria. They encompassed 11 human and six experimental studies.

Most clinical and preclinical studies report relieved depressive symptoms in relation to n-3 PUFA intake in menopause. Controversial results have been found in menopausal women on anxiety and cognitive functions, while in the few studies carried out in animal models n-3 PUFA reduced anxiety symptoms and improved cognitive functions.

Taken together, the current results show beneficial effects of n-3 PUFA on emotional and cognitive behaviors during menopause transition. However, further investigations should be performed to increase knowledge about the real effectiveness of n-3 PUFA on psychological well-being in this delicate period of feminine life.

## 124. Microglia-Released Extracellular Vesicles to Slow down the Aging Process in Relation to the Gender

Arianna Rinaldi, Cristina Limatola and Myriam Catalano

University of Rome “La Sapienza”, Department of Physiology and Pharmacology “Vittorio Erspamer”, Rome, Italy

Aging is a progressive deterioration of physiological functions characterized by accumulation of cellular damage, oxidative stress, and cellular senescence. Although aging is not a disease, it represents a significant risk factor for cognitive impairment and neurodegenerative disorders.

Aged brain presents a chronic, low-grade inflammatory state defined “inflammaging”, characterized by high oxidative stress, chronic inflammation, and high production of inflammatory compounds. During aging, modifications occur in microglia cells (the immune “sentinel” cells of the brain) which become hyper-responsive and nonfunctional. Consistently, these cells undergo the most prominent aging-related changes in both the morphological and functional phenotypes, differently affecting males and females. These events lead to dramatic consequences for brain homeostasis and central nervous system (CNS) cellular interactions.

Extracellular vesicles (EVs) are key players of the inter-cellular communication and are exploited by the cells to exchange information consisting of lipids, proteins, and nucleic acids. In the brain, EVs participate to neuron-glial cross-talk, synaptic modulation and can contribute to spreading disease in many CNS pathologies. Given their properties, EVs are emerging as a promising tool to develop revolutionary non-invasive therapies for a wide range of diseases.

Considering the above stated background, we investigated the effect of EVs released by not-inflamed microglia and intranasally administered to both male and female mice during the old age (16–18 months). We evaluated in vivo memory and motor coordination by behavioral tests and ex vivo inflammatory state of glia by RT-qPCR for inflammatory markers (IL-6, TNFα, IL1β, CD86). In EVs treated mice, we observed an increased memory and motor coordination and a reduction of all pro-inflammatory genes analyzed. The findings indicate EVs as an innovative strategy to slow down the effects of aging on brain functioning.

## 125. Counteracting Alpha-Synuclein Aggregation: A Novel Role for GM1 Oligosaccharide

Maria Fazzari ^1^, Giulia Lunghi ^1^, Erika Di Biase ^1^, Maria Grazia Ciampa ^1^, Laura Mauri ^1^, Ludovica Zaccagnini ^2^, Tim Bartels ^2^, Sandro Sonnino ^1^ and Elena Chiricozzi ^1^

^1^  University of Milan, Medical Biotechnology and Translational Medicine, Segrate, Italy 

^2^  University College London, UK Dementia Research Institute, London, UK

Parkinson’s disease (PD) is the second most common neurodegenerative disorder, characterized by the progressive loss of dopamine (DA) releasing neurons in the substantia nigra (SN). Fibrillary aggregated α-synuclein (αS) is a PD neurologic hallmark, considered to play a causative role in the disease. Although the causes leading to αS aggregation are not clear so far, the interaction with GM1 ganglioside is recognized to prevent this process. Recent evidence shows that the GM1 deficiency can lead to a failure of trophic plasma membrane signaling and to the αS accumulation, increasing the susceptibility to neuronal death. How GM1 exerts these functions is not clear, though a primary role of its soluble oligosaccharide portion (OligoGM1) is emerging. Indeed, we recently demonstrated that OligoGM1 is the bioactive portion of the ganglioside, able to modify the PD phenotype.

By Real-Time Quaking-Induced Conversion (RT–QuIC) assay, we demonstrated that OligoGM1 is able to prevent both the spontaneous and the prion-like (+PFF) αS aggregation. By circular dichroism spectroscopy of recombinant monomeric αS, we found that the administration of OligoGM1 do not induce any change in αS secondary structure (~5% helical, 95% random coil). Following, we proved the OligoGM1 efficacy in an in vitro model of PD: its administration significantly increases neuronal survival and preserves neurite networks of rat DA neurons affected by αS oligomers. Finally, using a PD mouse model, based on partial deletion of GM1 ganglioside, we found that OligoGM1 systemically administered is able to reduce the αS aggregates, completely rescuing the DA neurons and the motor impairments.

Our data demonstrate that GM1 ganglioside prevents the αS pathogenic aggregation through its oligosaccharide head, suggesting a possible role of age-dependent GM1 deficiency as a possible initiator for sporadic PD and the use of OligoGM1 as a possible therapeutic strategy.

## 126. The Role of Nrf2-Mediated System xc- Activation in HIV-1 Tat-Induced Neurotoxicity

Ludovica Carpinelli ^1^, Veronica D’Ezio ^1^, Marta Russo ^1^, Andrea Sperati ^2^, Cecilia Casella ^1^, Marco Colasanti ^1^ and Tiziana Persichini ^1^

^1^  University “ROMA TRE”, Department of Sciences, Rome, Italy 

^2^  University of Rome “LA SAPIENZA”, Department of Experimental Medicine, Rome, Italy

HIV-associated neurocognitive disorders (HANDs) affect a large part of HIV-infected patients, despite highly active antiretroviral therapy. HANDs occur in the absence of a direct infection of neurons. Nevertheless, viral proteins (e.g., Tat) are capable to cause neuronal dysfunction via oxidative stress, but the cellular pathways leading to HANDs are not yet fully defined. Here, we investigated the effects of Tat on Nrf2-mediated antioxidant response and system xc- expression in U373 human astroglial cells. The role of Tat-producing astrocytes on neuronal cell viability was assessed using SH-SY5Y cells as a culture model. We demonstrated that Tat produced by astrocytes was able to induce Nrf2 activation and system xc- expression in astrocytes, thus reducing cell viability of co-cultured neuronal cells. Sulfasalazine, a specific system xc- inhibitor, was able to reduce extracellular glutamate and to prevent the reduction of neuronal viability, thus demonstrating that the neurotoxic effect was dependent on an increased glutamate release through the transporter. Moreover, we investigated on the efficacy of bovine lactoferrin (bLf), in both its native and iron-saturated (holo-bLf) forms, in counteracting oxidative stress in astroglial cells constitutively expressing HIV-Tat protein. Our findings provide evidence of the involvement of astroglial Nrf2/system xc- pathway in the neurotoxicity induced by HIV-1 Tat protein, thereby suggesting how astrocytes may exacerbate neurodegeneration through the conversion of an antioxidant response to excitotoxicity.

## 127. Mitochondrial Dysfunctions in Spinal Muscular Atrophy: Mitochondrial Aconitase as a Potential Biomarker of the Disease

Gianna Pavarino ^1^, Serena Stanga ^1^, Francesco Paolo Zummo ^2^, Alessandro Vercelli ^1^ and Marina Boido ^1^

^1^  Neuroscience Institute Cavalieri Ottolenghi, University of Turin, 10043 Orbassano (TO), Department of Neuroscience “Rita Levi Montalcini”, University of Turin, 10126 Turin, Italy 

^2^  Department of Biomedicine, Neurosciences and Advanced Diagnostics (BiND), University of Palermo, 90127 Palermo, Italy

Spinal muscular atrophy (SMA) is a pediatric and juvenile onset neurodegenerative disease due to a mutation/deletion of the Survival Motor Neuron 1 (SMN1) gene, which causes the selective and early death of spinal cord (SC) motor neurons following the decrease of functional SMN protein levels. Despite the genetic cause of SMA is known, many aspects of its pathogenesis are still elusive. In the last years, mitochondrial alterations have been found during the pre-symptomatic stages of the disease and now are considered a risk factor for SMA. Therefore, we decided to deepen the study of such dysfunctions in SMA both at central (SC of postnatal day 7 SMN∆7 mice, a severe SMA model) and peripheral (SMN∆7 and human fibroblasts) level. From a screening with 2-DE-MALDI-TOF-MS on pure mitochondria isolated from SC, we noticed altered expression and post-translational ubiquitination of the mitochondrial Aconitase (mAcn) enzyme, together with a strong reduction (<40%) of its functionality. Moreover, by Western blotting analysis, we identified alterations in mitochondrial dynamism (increased fission and decreased fusion) and respiration without any change in mitochondrial content. Interestingly, mAcn alterations were present also in fibroblasts derived from SMN∆7 embryos and SMA patients. Murine fibroblasts showed a decreased mAcn functionality (<60%) and there was completely no mAcn activity in two out of three SMA patients. MitoTracker staining of SMA murine and human fibroblasts followed by mitochondrial network analysis revealed an increase of mitochondrial footprint and individuals confirming the tendency to network fragmentation. Moreover, SMA human fibroblasts also displayed a remarkable reduction (−3 folds) of mitophagic processes. Overall, such data also show alterations of mAcn activity in peripheral cells and suggest this enzyme as a potential biomarker of the disease, eventually to be tested even in blood cells that can be collected by minimally invasive procedures.

## 128. Niclosamide Ameliorates Disease Progression in a Model of Amyotrophic Lateral Sclerosis

Ilaria Della Valle ^1^, Martina Milani ^1^, Simona Rossi ^2^, Mauro Cozzolino ^2^, Nadia D’Ambrosi ^1^ and Savina Apolloni ^1^

^1^  Università degli studi di Roma Tor Vergata, Biologia, Roma, Italy 

^2^  Centro Nazionale delle Ricerche (CNR), Ististuto di farmacologa traslazionale, Roma, Italy

Amyotrophic lateral sclerosis (ALS) is a neurodegenerative disease caused by interactions between genetic, epigenetic, and environmental factors, with consequent dysfunction of multiple cellular and molecular pathways. The multifactorial nature of the disorder could explain the modest results obtained by the treatments proposed so far and highlights the need for multitarget therapies acting synergistically on different aspects of the disease. Niclosamide is on the WHO list of essential medicines, already used for decades as an anthelminthic. It has recently been repurposed in clinical trials for its potent anti-inflammatory and anti-fibrotic properties. It is well documented that niclosamide can inhibit different molecular pathways (e.g., STAT3, Wnt/b-catenin, SQSTM1/p62, NF-kB), which, importantly, are dysregulated in ALS, suggesting its potential use to interfere with these mechanisms in the pathology. We found that niclosamide inhibits microglia reactivity, reduces inflammation and fibrosis, and promotes autophagy in familial and sporadic ALS fibroblasts. Further, in a proof-of-concept study conducted in a model of ALS, i.e., hFUS mice, niclosamide strongly inhibited inflammation and fibrosis and promoted autophagy and regeneration in the nervous system and skeletal muscles. This work aims to perform a preclinical validation of the drug in the hFUS-ALS model to analyze disease progression and investigate pathogenic mechanisms targeted by niclosamide. We demonstrated that niclosamide injected intraperitoneally starting at symptom onset ameliorates grip strength and behavioral scores, increasing mice disease duration and survival. Moreover, niclosamide increases BBB integrity and decreases motoneuron loss, axonal damage and microgliosis in the spinal cord of hFUS-treated mice. These data suggest that a cheap and well-explored drug such as niclosamide can slow down the progression of the disease in ALS mice, making it a promising candidate to be repositioned for ALS.

## 129. Characterization of the Early Cognitive, Emotional, Motor, and Behavioral Features of a Mouse Model of Parkinson’s Disease

Francesca Balsamo ^1^, Maria Meringolo ^2^, Martina Montanari ^3^, Paola Bonsi ^2^ and Francesca Gelfo ^1^

^1^  Santa Lucia Foundation, IRCCS; Guglielmo Marconi University, Department of Human Sciences, Rome, Italy 

^2^  Santa Lucia Foundation, IRCCS, Rome, Italy 

^3^  Santa Lucia Foundation, IRCCS; Tor Vergata University, Department of Systems Neuroscience, Rome, Italy

Parkinson’s disease (PD) is a chronic and progressive neurodegenerative disorder mainly characterized by resting tremor, rigidity, bradykinesia, and postural instability. PD is a genetically heterogeneous disorder. Among the monogenic forms, the PTEN-induced putative kinase 1 (PINK1) mutation is the second most frequent cause of early-onset PD, being associated to a pathological mechanism involving the slow progressive loss of physiological functions. Although PD is considered primarily a motor disorder, growing evidence suggests the presence of a wide spectrum of non-motor symptoms appearing from early stages of the disease.

Cognitive impairment is one of the most common and important non-motor features of PD. Indeed, several animal and human studies demonstrated the presence of cognitive and behavioral disorders in patients with PD, which may affect different domains, such as attention, visuospatial and executive functions, learning, and memory, and neuropsychiatric symptoms, such as anxiety and depression.

The present study, funded by the Italian Ministry of Health (RF-2019-12370182), was aimed at investigating a PINK1 mouse model of PD to evaluate the presence of motor, cognitive, emotional, and behavioral symptoms at an early stage of the disease and characterize them. We compared two-month-old PINK1 knock-out mice with PINK1 wild-type controls. All mice were submitted to a behavioral assessment battery consisting of: Novel Object Recognition Test and Y-Maze Spontaneous Alternation Test (cognitive functions); Elevated Plus Maze Test, Forced Swim Test, and Splash Test (emotional behaviors); Rotarod Test (locomotor capabilities). Preliminary data suggested the presence of alterations in specific aspects of cognitive and emotional components, which appeared not accompanied by impairments in the motor behavior of knock-out mice. These results support the potential of the PINK1 PD model as a basis in studying early functional symptoms to design effective and tuned treatments.

## 130. CCT5 Variants Associated with Sensory and Motor Neuropathies: An In Silico Study

Federica Scalia ^1,2^, Giosuè Lo Bosco ^1,3^, Letizia Paladino ^1,2^, Alessandra Maria Vitale ^1,2^, Everly Conway de Macario ^4^, Alberto J. L. Macario ^1,4^, Fabio Bucchieri ^2^, Francesco Cappello ^1,2^ and Fabrizio Lo Celso ^5,6^

^1^  Euro-Mediterranean Institute of Science and Technology (IEMEST), 90139 Palermo, Italy

^2^  Department of Biomedicine, Neuroscience and Advanced Diagnostics (BIND), University of Palermo, 90127 Palermo, Italy

^3^  Department of Mathematics and Computer Science, University of Palermo, 90123 Palermo, Italy

^4^  Department of Microbiology and Immunology, School of Medicine, University of Maryland at Baltimore-Institute of Marine and Environmental Technology I, Baltimore, MD, USA 

^5^  Ionic Liquids Laboratory, Institute of Structure of Matter, Italian National Research Council (ISM-CNR), 00133 Rome, Italy

^6^  Department of Physics and Chemistry, Emilio Segrè, University of Palermo, 90128 Palermo, Italy

Hereditary neurochaperonopathies are a group of heterogenous disorders of the nervous and neuromuscular system associated with mutations in genes encoding for molecular chaperones and chaperonins. Different genetic modifications which occur in a gene encoding for chaperones or chaperonins can lead to distinct phenotypes. Nowadays, poor information about the etiologic-pathogenic role of these molecules is available making difficult the identification of the pathological grade, e.g., how, and how much, the skeletal muscle tissue is involved, or the prognosis. The chaperonin containing TCP1 complex (CCT) is a hetero-oligomeric complex constitutively expressed by all human cytotypes. It is made up of two overlapped rings, each consisting of eight subunits (named from CCT1 to CCT8), and it is able to fold about the 15% of cytosolic proteins. Two point mutations in the gene encoding for the CCT subunit 5 (CCT5), p.(His147Arg) and p.(Leu224Val), are associated with sensory and motor neuropathies, respectively. In the present work, we discuss the clinical differences between the patients affected and show, through in silico analysis, the conformational changes and the differences in physicochemical features between CCT5 p.(His147Arg) and CCT5 p.(Leu224Val) variant, and when they are compared to the wild type subunit. The apical domain of both variants appears mainly but differently affected. The hydrogen bonds distribution and the electrostatic potential of the mutated subunits differ widely compared to the wild type molecule. We suggest that the heterogeneity observed in gene mutation, phenotype, disease onset, and progression may be the mirror of the differently modified allosteric contribution of the CCT5 variants within the CCT complex. Due to the phenotypic heterogeneity of these disorders, they are hardly identified and undiagnosed. For this reason, we recommend the investigation of molecular chaperone gene variant when neuromyopathies is prevalent.

## Data Availability

The data that support the findings, upon reasonable request.

